# DNA methylation: a potential mediator between air pollution and metabolic syndrome

**DOI:** 10.1186/s13148-022-01301-y

**Published:** 2022-06-30

**Authors:** Parinaz Poursafa, Zoha Kamali, Eliza Fraszczyk, H. Marike Boezen, Ahmad Vaez, Harold Snieder

**Affiliations:** 1 Department of Epidemiology, University of Groningen, University Medical Center Groningen, Groningen, the Netherlands; 2 Department of Bioinformatics, Isfahan University of Medical Sciences, Isfahan, Iran

**Keywords:** Epigenetics, DNA methylation, Metabolic syndrome, Air pollution

## Abstract

Given the global increase in air pollution and its crucial role in human health, as well as the steep rise in prevalence of metabolic syndrome (MetS), a better understanding of the underlying mechanisms by which environmental pollution may influence MetS is imperative. Exposure to air pollution is known to impact DNA methylation, which in turn may affect human health. This paper comprehensively reviews the evidence for the hypothesis that the effect of air pollution on the MetS is mediated by DNA methylation in blood. First, we present a summary of the impact of air pollution on metabolic dysregulation, including the components of MetS, i.e., disorders in blood glucose, lipid profile, blood pressure, and obesity. Then, we provide evidence on the relation between air pollution and endothelial dysfunction as one possible mechanism underlying the relation between air pollution and MetS. Subsequently, we review the evidence that air pollution (PM, ozone, NO_2_ and PAHs) influences DNA methylation. Finally, we summarize association studies between DNA methylation and MetS. Integration of current evidence supports our hypothesis that methylation may partly mediate the effect of air pollution on MetS.

**Supplementary Information:**

The online version contains supplementary material available at 10.1186/s13148-022-01301-y.

## Background

Air pollution is a well-known health problem worldwide leading to increased morbidity and mortality [1]. Air pollution has been associated with several human diseases including metabolic disorders [1]. While the association between air pollution and metabolic syndrome (MetS) is well established, the exact underlying mechanism(s) explaining this relation remains to be elucidated. Here, we review the associations between air pollution and MetS-related traits, between air pollution and DNA methylation, and between DNA methylation and MetS (Additional file 1: Table S1). Finally, we integrate the evidence and propose that DNA methylation potentially mediates the effect of air pollution on MetS.

### Measurement of air pollution

Six popular air pollutants are known as ‘criteria air pollutants’ including ozone (O_3_), particulate matter (PM), carbon monoxide (CO), sulfur dioxide (SO_2_), lead, and nitrogen dioxide (NO_2_) (Table 1). The air quality index (AQI) is based on daily measurement of all criteria pollutants except lead. Most methods for measurement of gaseous air pollutants use in situ continuous monitors for hourly averaged concentrations. PMs are typically measured using combined sampling systems over a 24-h period with defined inlets, pumps, sampler surfaces, etc. (Additional file 1: Table S2) [2]. Polycyclic aromatic hydrocarbons (PAHs) are another group of chemicals widely distributed in the atmosphere that also constitute an important source of air pollution (Table 1). PAHs are ubiquitous environmental pollutants that are formed during the incomplete burning of coal, oil, gas, and tobacco. PAHs generated from these sources can bind to or form small particles in the air [3].

**Table 1:** Major sources of ambient air pollutants

Pollutant	Major sources
Ozone (O_3_)	Formed from nitrogen oxide species and volatile organic compounds by human activities (largely the combustion of fossil fuel)
Particulate matter (PM)	Naturally occurring, originates from volcanoes, dust storms, forest and grassland fires, living vegetation, and sea spray. Human activities, such as the burning of fossil fuels in vehicles, power plants, and various industrial processes also generate significant amounts of aerosols
Carbon monoxide (CO)	Is a product of fuel combustion such as natural gas, coal, or wood. Vehicular exhaust contributes to the majority of carbon monoxide in the atmosphere
Sulfur dioxide (SO_2_)	Is produced by volcanoes and in industrial processes. Combustion of coal and petroleum generates sulfur dioxide, as they often contain sulfur compounds
Lead	Ore and metals processing and piston engine aircraft operating on leaded aviation fuel. Other sources include waste incinerators, utilities, and lead–acid battery manufacturers. The highest lead concentration in air is usually found near lead smelters
Nitrogen dioxide (NO_2_)	Nitrogen dioxide is expelled from high-temperature combustion and is also produced during thunderstorms by electric discharge
Polycyclic aromatic hydrocarbons (PAHs)	Are formed during the incomplete burning of coal, oil, gas, and tobacco

Data retrieved from Wikipedia and United States Environmental Protection Agency Web site (www.epa.gov)

The International Agency for Research on Cancer (IARC) Working Group on the Evaluation of Carcinogenic Risk to Humans has provided a complete list of methods for the measurement of air pollutants [4]. There are a number of challenges and concerns related to exposure assessment for ambient air pollution in general and actual PM exposure specifically, especially in regions where there is little air quality monitoring. In large-scale epidemiological studies of PM and health, ambient fixed-location monitors are used to assign exposure. Then, the associations with different health outcomes are estimated using single-pollutant regression models. While this approach provides a general framework for estimating associations between air pollution and health, ambient monitors are often sparse and some of them do not provide daily measurement. Also, there might be some instrument or sampling errors. In order to enrich the limited data, integrative techniques such as the Data Integration Model for Air Quality (DIMAQ) have been developed, which provide estimates of exposures to PM_2.5_ at a high resolution. This method incorporates different data sources including ground measurements, satellite remote sensing, population estimates, topography, and information on local monitoring networks and measures of specific air pollution indices from chemical transport models. Dedicated methods for estimating exposure–risk relationships such as the integrated exposure–response function (IER) have been developed, which estimates the relative risk for a disease caused by air pollution exposure from PM_2.5_ [5, 6].

### Air pollution as a global health problem

**Fig. 1:**
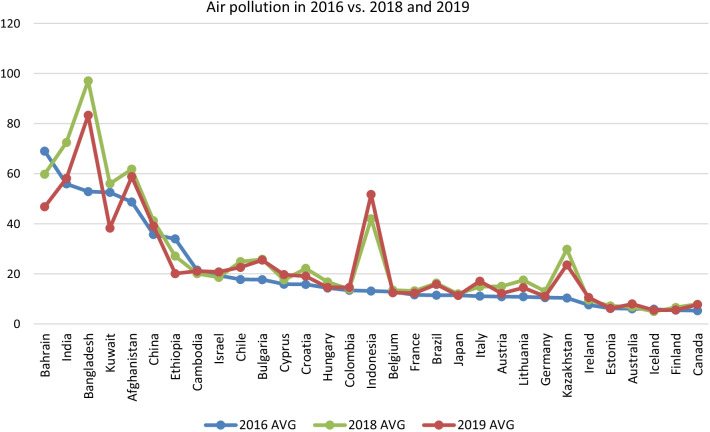
Average air pollution (PM_2.5_) across 31 countries where data were available for all three time spots of 2016, 2018, and 2019. WHO GHO repository (https://www.who.int/data/gho/data/indicators/indicator-details/GHO/concentrations-of-fine-particulate-matter-(pm2-5)) and IQair Web site (https://www.iqair.com) are used to retrieve average air quality indices (AVG). *P* values of one-tailed paired *T* tests for 2016 vs. 2018, and 2019 measures were 0.0028 and 0.067, respectively

Air pollution has become a global health problem. The World Health Organization Global Burden of Disease (GBD) Comparative Risk Assessment study estimates the burden of disease attributable to air pollution in terms of deaths and disability-adjusted life years (DALY). The GBD Study from 2015 [7] reports ambient PM_2.5_ (particulate matter with a diameter ≤ 2.5 μm) as the fifth-ranking mortality risk factor in 2015, which amounts to about 4.2 million deaths, and 103.1 million DALYs in 2015 and it found an increasing trend of deaths attributable to ambient PM_2.5_ from 1990 to 2015. This could be because of rising levels of pollution and increasing number of deaths from non-communicable disease (NCDs) in the largest low-income and middle-income countries with growing populations. Except the two recent years being impacted by COVID-19 lockdown, increasing air pollution since 2016 has been reported in many countries [8–10] (Fig. 1). Moreover, WHO reports that 91% of the global population live in places exceeding air quality guidelines. Mortality and morbidity are mostly related to cardiovascular and respiratory outcomes [7–11]. To further investigate AP attributable mortality, we retrieved the AP attributable death rate per cause from WHO Global Health Observatory (data were available for 2016), alongside with their air quality using the PM_2.5_ index (in the same year, i.e., 2016, from the same database). AP attributable death rates for the top five countries with the highest PM_2.5_ index are given in Table 2. GBD Study 2015 also ranks ambient ozone as the 34th risk factor for global deaths and 42nd risk factor for DALYs which amounts to causing 254,000 deaths globally and a loss of 4.1 million DALYs in 2015.

**Table 2:** Ambient air pollution attributable death rate by cause (per 100,000 population, age-standardized) for top 5 polluted regions in 2016 (based on PM_2.5_ concentration in ug/m^3^), where WHO data were available for both PM_2.5_ and outcomes

Location	PM_2.5_	COPD	Trachea, bronchus, lung cancers	Lower respiratory infections	Stroke	IHD	Total
Niger	93.18	10.96	0.22	58.26	20.59	34.95	125
Nepal	81.57	28.88	4.39	19.67	17.41	42.01	112.4
Qatar	80.8	3.13	3.39	4.05	4.12	31.7	46.4
Mauritania	74.13	6.73	0.28	35.95	13.55	31.63	88.15
Egypt	72.31	10.2	2.3	11.25	19.38	61.9	105

Data are retrieved from Global Health Observatory (GHO)—WHO, 2016. PM_2.5_: particulate matter with a diameter ≤ 2.5 μm; COPD: chronic obstructive pulmonary disease; IHD: ischemic heart disease

## Air pollution and metabolic syndrome

MetS is characterized by the clustering of cardiometabolic risk factors. There are different definitions of MetS most of which are quite similar apart from some differences in the cutoffs of the components. The shared feature of all definitions is that MetS is considered as the coexistence of at least three out of five components including abdominal obesity, disorders in fasting blood glucose, triglycerides, blood pressure, and HDL cholesterol [12, 13]. MetS is a known predisposing factor for a number of chronic, non-communicable diseases of which type 2 diabetes and cardiovascular diseases are the most important [14]. As such MetS is an important public health concern. Several predisposing factors are shown to modify the risk of MetS, including (but not limited to) genetic factors, diet, physical activity, smoking, education, age, sex, race, ethnicity, socioeconomic status, and urban-versus-rural residence [15]. Hence, like any other complex trait, MetS is a consequence of both genetic and environmental factors such as air pollution, and their complex interaction [16]. Several lines of evidence indicate that exposure to air pollutants has a crucial role in the development and progress of MetS both in adults and in children [17–19]. One potential consideration in cross-sectional association studies of air pollution and MetS is the possible confounding effect of socioeconomic status. Like MetS, air pollution exposure may also relate in part to socioeconomic status. However, studies on this relationship are not conclusive [20, 21]. There is some evidence of a nonlinear relationship between air pollution and MetS, where socioeconomic status seems to intensify the association, but does not explain it entirely [22, 23]. However, longitudinal studies in which air pollution exposure precedes MetS occurrence without any significant individual changes in socioeconomic status during follow up strengthen the hypothesis [24, 25]. A narrative review in firefighters showed association of air pollutants and MetS in World Trade Center (WTC)-exposed Fire Department of New York. This study found that exposure to particulate matter provokes pulmonary and systemic inflammation. Systemic inflammation leads to the development of MetS and cardiopulmonary disease, such as chronic obstructive pulmonary disease and cardiovascular disease. Individuals with these conditions are more susceptible to the inflammatory effects of PM exposure, which can aggravate their conditions [26].

Some cross-sectional studies in adults documented that exposure to ambient air pollutants is independently associated with risk of MetS and related chronic diseases [27, 28]. The observed associations between air pollution exposure and MetS were sensitive to MetS definitions. Regarding the MetS components, strongest associations were observed with impaired fasting glycemia, and positive but weaker associations with hypertension and waist-circumference-based obesity. Cardiometabolic effects of air pollution may be majorly driven by impairment of glucose homeostasis and, to a lesser extent, visceral adiposity [28]. In a nationwide study on 1,413 children with a mean (SD) age of 14.81 (2.48) years, we showed that those living in areas with higher air pollution had higher levels of MetS components including increased systolic blood pressure, fasting blood glucose, and triglycerides, as well as lower levels of HDL-C. The results remained significant even after adjustment for confounding factors including age, sex, and anthropometric measures, as well as for dietary and physical activity habits [29]. In a previous study, we have shown that exposure to air pollutants increases the risk of underlying MetS components [17] which is in line with longitudinal studies [15]. Additionally, we have recently shown for the first time that independent of weight status, exposure of children to PAHs is significantly associated with two components of MetS, namely triglycerides and fasting blood glucose. This study can partly explain, why not all obese persons have a higher risk for all MetS components, and why some normal-weight individuals do show these risk factors. Hence, this study provided confirmatory evidence of the role of exposure to air pollutants in the development of MetS and its components [30]. In this review, we will take a closer look at the association of air pollution with MetS and will explore possible mechanisms explaining this association.

## Putative mediating mechanisms of air pollution exposure in the pathogenesis of MetS

Endothelial dysfunction, a systemic pathological state of the inner lining of blood vessels, is one of the contributors of the complex pathophysiology of MetS [16, 30]. The condition is defined as an imbalance between vasodilating and vasoconstricting substances acting on the endothelium. Endothelial dysfunction can be measured by its serum markers such as endothelin-1, vascular adhesion molecule, and intercellular adhesion molecule. It is also assessed by measuring flow-mediated dilation of the brachial artery by ultrasound [31].

We and others have shown that endothelial dysfunction is associated with air pollution [32–34]. Our study showed that healthy children and adolescents with higher exposure to air pollutants, notably PM, had higher levels of markers of endothelial dysfunction and a possible pro-coagulant state [32]. In another study on prepubescent boys, we found that living in air-polluted areas was significantly associated with attenuated endothelium-dependent brachial artery dilation, i.e., a known marker of endothelial dysfunction [35]. In both the above-mentioned studies, we noticed that the association of air pollutants with markers of endothelial dysfunction was independent of weight status or lifestyle of participants.

In a population-based cohort of 233 mother–neonate pairs, we found that exposure to ambient air pollution during pregnancy was associated with cord blood concentrations of surrogate markers of endothelial dysfunction including endothelin-1, vascular adhesion molecule, and intercellular adhesion molecule [36]. Collectively, these studies suggest that endothelial dysfunction may be a mediating mechanism between air pollution and MetS.

Another possible mediator of air pollution effects on MetS might be the production of reactive oxygen species (ROS). These agents can affect the methylation–demethylation circuit in the nucleus, by direct modifications of the methylated CpG sites or by altering expression of involved enzymes, which in turn can lead to systemic (mostly hypomethylation) and locus-specific epigenetic changes in DNA [1]. The interplay of the air pollution-induced ROS reagents with inflammation [34, 37, 38] can lead to a variety of pathologic conditions including MetS components such as dyslipidemia and glucose intolerance [37].

## Epigenomics—DNA methylation

Epigenetics is a field that investigates alterations in the DNA molecule that do not change the DNA sequence, but that can lead to differences in gene expression levels and hence variations in health outcomes [39–41]. Epigenetic processes mainly include histone modifications and DNA methylation. DNA methylation in blood is the most widely investigated epigenetic factor [42] on which we will focus in this review. DNA methylation is a chemical modification of DNA through the addition of a methyl group to the DNA molecule, which mainly happens at cytosine–guanine dinucleotide (CpG) sites [43, 44]. Modification of DNA methylation at the promoter region of the gene will typically lead to changes in gene expression. That is why the study of alterations in DNA methylation is important in understanding complex mechanisms underlying multifactorial diseases and traits [45, 46].

DNA methylation analyses of the candidate genes are hypothesis-based investigations of the association between DNA methylation of a gene and a particular trait or disease. Although this is an appropriate design and requires smaller sample sizes than genome-wide studies, there are criticisms about the possibility of false positives and hence, poor replication of the results of such studies (also called type I error). Thanks to reductions in costs and availability of microarray technology, genome-wide investigations of DNA methylation markers are now possible. The hypothesis-free experimental design of epigenome-wide association study (EWAS) has been applied during the past few years to unravel epigenomic (i.e., DNA methylation) alterations underlying complex traits and diseases. The first genome-wide methylation array was the Illumina Infinium HumanMethylation27K BeadChip [47]. However, most EWAS studies so far have applied the Illumina Infinium HumanMethylation450K BeadChip array for their DNA methylation profiling. The latter measures the methylation levels of over 485,000 CpG sites per genome. The most recent methylation array covers over 850,000 methylation sites per sample and, given sufficient sample sizes to avoid false-negative results (type II error), promises a new wave of successful EWAS studies with better resolution of the results [48].

## Modification of DNA methylation by air pollution

DNA methylation is influenced by both environmental and genetic factors. It has been documented that DNA methylation alterations are resulting from a wide range of different environmental exposures such as smoking, physical activity, stress, diet, and toxins [49–55]. Thus, the epigenome is increasingly being proposed as a vital link between environmental exposures and gene expression modifications. A growing body of evidence now shows that air pollution influences DNA methylation [56–64].

Rossnerova et al. compared the methylation profiles in 200 blood samples of children from two regions with different levels of air pollution. Air pollution measurement was performed by the Czech Hydrometeorological Institute in a highly polluted region and a control region in the Czech Republic. Using the Human Methylation 27 K BeadChip with 27,578 CpG sites, they found ~ 36% (9,916) of CpG sites had significantly different methylation levels. A total of 58 out of 9,916 CpG sites had > 10% lower methylation levels in children from polluted area, associated with higher gene expression in comparison with the control region in the children from the polluted region compared to the children from the control region [65].

Below, we review the available evidence for the association of a number of specific air pollutants with DNA methylation, namely PM, O_3_, NO_2_, and PAH.

### Particulate matter and DNA methylation

Particulate matter (PM) constitutes a complex mixture of very small particles and liquid droplets that get into the air. These small particles, regardless of their chemical composition, can cause serious health effects as they can pass biological barriers. Airborne PM may induce epigenetic changes. Several studies have linked short- and mid-term PM exposure to global and gene-specific DNA methylation [66–72]. Some studies found that exposure to PM_2.5_ may affect DNA methylation of certain candidate genes related to coagulation and inflammation [73–78]. Likewise, a randomized, double-blind crossover trial showed that short-term exposure to PM_2.5_ was associated with rapid global DNA hypomethylation [68]. A recent EWAS on 2,956 participants from three cohorts of European ancestry studied the association between DNA methylation and cumulative PM_2.5_ exposure averaged over 2 days up to 4 weeks using the Illumina 450 k BeadChip [79]. Applying a stringent genome-wide Bonferroni significance level (*p* ≤ 7.5E−8), revealed 12 CpG sites (*ACVR2B-AS1*, *ACYP2*, *C1orf212*, *F2*, *MN1*, *MSGN1*, *NEURL4*, *NSMAF*, *NXN*, *SERBP1*, *TSPYL6*, and *ZMIZ1*). Applying a more lenient false discovery rate significance level increased the number of associated CpG sites to more than 1,800. Although the results still need replication in an independent study population, this study provides evidence of the effect of PM_2.5_ pollution on DNA methylation. Based on the identified CpG sites, this study suggests biological pathways that might mediate associations between PM_2.5_ and health outcomes such as glucose metabolism [79]. A later EWAS on 646 participants using the Illumina 450 k BeadChip array showed that long-term exposures to some PM_2.5_ species (Fe, Ni, V), mostly combustion emitted particles, were associated with alterations in DNA methylation in immune response genes. In this study, 20 Bonferroni significant (*P* value < 9.4 × 10–9) CpGs were found for Fe, 8 for Ni, and 1 for V [80].

In addition to PM_2.5_, several lines of evidence show PM_10_ (particulate matter ≤ 10 μm) is associated with DNA methylation of some candidate genes related to cardiovascular and respiratory diseases as well as inflammatory immune responses and oxidative stress [51, 61, 74, 81]. A recent EWAS provides evidence for association of PM_10_ exposure and altered DNA methylation of multiple loci [81]. Moreover, animal studies have also investigated the effects of particulate matter on DNA methylation [58, 72], but the focus of this review is mostly on human studies.

### Ozone and DNA methylation

Ozone (O_3_) is created by chemical reactions between oxides of nitrogen (NOx) and volatile organic compounds in the presence of sunlight. There is evidence that ozone air pollution is associated with global DNA methylation and interferon gamma (*IFN-γ*) hyper-methylation, as well as its protein level [82]. IFN-γ has an important role in immune responses to a foreign compound. Ozone exposures (2–4 weeks) were also negatively related to intercellular adhesion molecule 1 (ICAM-1) methylation [75].

Another study showed that acute exposure to ambient ozone was associated with higher blood pressure and with increased serum levels of angiotensin-converting enzyme (ACE) and endothelin-1 (ET-1) as biomarkers of blood pressure. Results of this study supported an instant hypomethylation response of the *ACE* and *EDN1* genes due to ozone exposure, but there was no significant mediation of DNA methylation in the effects of ozone on blood pressure [83].

### NO_2_ and DNA methylation

Nitrogen dioxide (NO_2_) is formed and released into the air from burning of fuel, for example from car emissions, and is related to a wide range of adverse health effects [84]. A recent study shows that short-term exposure to NO_2_ is associated with DNA methylation of two respiratory function related genes, i.e., arginase (*ARG2*) hypermethylation and inducible nitric oxide synthase (*NOS2A*) hypomethylation [85].

A recent large-scale EWAS on 1508 newborns from four European and North American birth cohort studies investigated the association between maternal NO_2_ exposure during pregnancy and DNA methylation in newborns using the Illumina 450 k BeadChip. Three CpG sites in mitochondria-related genes (*LONP1*, *HIBADH*, and *SLC25A28*) became significant after correcting for multiple testing (FDR < 0.05) [86]. In another more recent large-scale EWAS on 1,017 subjects from the Lifelines cohort study using the Illumina 450 k BeadChip, significant associations were found between long-term NO_2_ exposure and DNA methylation for seven CpG sites (Bonferroni-corrected threshold *p* < 1.19 × 10^−7^) or for 4980 CpG sites (FDR < 0.05). Furthermore, two Bonferroni-corrected significant CpG sites significantly mediated the association between NO_2_ and lung function [87].

A recent EWAS investigating whether NO_2_ during pregnancy is associated with differences in placental DNA methylation levels showed that methylation level of 2 CpGs located in the *ADORA2B* gene, whose expression is associated with hypoxia and pre-eclampsia, were significantly associated with NO_2_ exposure during the pregnancy (FDR < 0.05) [88]. These two studies show that maternal exposure to air pollution can lead to modification of DNA methylation patterns—and potential adverse health outcomes—in the offspring.

Another EWAS in a Korean chronic obstructive pulmonary disease cohort investigated association with long-term ambient air pollution exposure and found 45 CpGs related to NO_2_ (FDR < 0.05). Enriched networks based on these results were related to outcomes associated with air pollution such as cardiovascular and respiratory diseases as well as inflammatory and immune responses [81].

### PAHs and DNA methylation

PAHs are highly lipid soluble and could have a role in obesity and development of MetS and its components [30, 89]. These chemicals can form or bind to small particles in the air. Exposure to PAHs might have epigenetic effects so that DNA methylation is considered to provide a novel marker of the environmental impact of PAH exposure on gene function [90]. Several lines of evidence showed an association of PAHs and DNA methylation in both global methylation and candidate genes studies [90–94]. Moreover, a recent study using the 450 k array reported the association of PAHs with an epigenetic predictor of accelerated aging [95].

## Methylation and its effect on MetS

It is believed that chronic diseases are complex consequences of long-term exposures to risk factors before clinical onset of the diseases. According to the Developmental Origins of Health and Disease (DOHaD) hypothesis, intrauterine or postnatal adaptations to adverse environmental exposures might cause several physiologic or metabolic alterations affecting health later in life. This hypothesis proposes an association of suboptimal intrauterine development with risk of chronic disorders, including MetS, in adult life [64, 96–98]. The mechanisms through which fetal programming as well as early-life exposures may affect physiologic or metabolic status in later life is still to be elucidated, but epigenetic variation may offer a good explanation [99–102]. Epigenetic variation, either early or late in life, may play an important role in the complex interplay between genetic susceptibility and environmental exposures which can eventually result in metabolic alterations. In a comparison between normal infants and those with intrauterine growth restriction (IUGR), Williams et al. reported a global shift toward hypermethylation in IUGR infants [103]. In a recent meta-analysis of EWASs totaling 8825 neonates from 24 birth cohorts in the PACE (Pregnancy And Childhood Epigenetics) Consortium, we found that DNA methylation in neonatal blood is genome-wide significantly associated with birthweight at 914 sites, with a difference in birthweight ranging from −183 to 178 g per 10% increase in methylation [102].

A growing body of evidence supports the role of methylation changes in metabolic dysregulation including MetS [66, 104]. A review on the subject concluded that modifications in DNA methylation may contribute to MetS susceptibility [42]. Epigenetics could have a role in affecting obesity and cardiometabolic diseases, by activating or silencing the relevant genes. Scientific evidence has suggested that *LINE-1* methylation is associated with obesity-related diseases, as well as insulin resistance, type 2 diabetes mellitus, and cardiovascular disease [105]. A recent epigenome-wide study in African-American adults with high prevalence of MetS showed that MetS was consistently associated with increased methylation in the *ABCG1* gene [106]. Other epigenome-wide investigations showed methylation sites associated with components of MetS, including central obesity, insulin responsiveness, and type 2 diabetes, as well as lipid profiles in different populations [107–109].

Elevated blood pressure is another component of MetS. An early pilot study conducted by us already showed difference in a single blood leukocyte DNA methylation site between hypertensives and controls [70]. In a more recent study, the CHARGE (Cohorts for Heart and Aging Research in Genomic Epidemiology) consortium conducted a meta-analysis of EWASs investigating cross-sectional associations with systolic and diastolic blood pressure and found 13 CpGs replicated after Bonferroni correction. In a later study, they found and cross-validated 34 CpG sites for blood pressure using two large datasets of over 4000 and 17,000 individuals. Six of the identified CpG sites also showed significant association (FDR < 0.05) with gene expression (*PHGDH*, *ABCG1*, *LMNA*, *RBPMS2*, and *SLC1A5*). Further investigation of these CpG sites in a meta-analysis of twin cohorts suggested that the majority of correlation between DNA methylation and blood pressure can be explained by shared environmental factors. Findings of these studies showed that heritable DNA methylation may play a role in regulating blood pressure [110, 111].

Recently, we performed a systematic review and replication to test the association between DNA methylation and glycemic traits including type 2 diabetes. We showed that a number of EWAS signals of type 2 diabetes in the literature can be significantly replicated providing confidence in the association between DNA methylation and type 2 diabetes [112]. Moreover, a number of recent EWASs of obesity-related traits provide convincing evidence for association with DNA methylation at numerous CpG sites [113–116]. However, the direction of association has been a matter of debate. Although Wahl et al. report changes in DNA methylation at 187 genetic loci to be associated with BMI, their analyses indicate that DNA methylation changes are more likely the effect of obesity rather than the cause [115].

In animal studies, increasing the diversity of environmental interventions in the study of metabolic health will provide a more complete picture of the interactions between environment, epigenetic and genetic background, and metabolic health. Both global and locus-specific differential DNA methylation have been observed in rodent models of metabolic syndrome [117].

## DNA methylation may mediate the effect of air pollution on MetS

MetS is one of the major challenges worldwide. The association between MetS and air pollution is of great concern. Epigenetic modifications, including DNA methylation, have been identified as one mechanism by which the environment interacts with the genome with alterations in DNA methylation potentially contributing to the development of metabolic diseases [118, 119].

A systematic review of studies in children concluded that exposure to environmental pollutants may have an important role in the origin of various disorders including MetS. Environmental pollutants trigger epigenetic imbalances, which may lead to increasing risk of MetS [50]. The epigenome is increasingly being proposed as a vital link between environmental exposures and gene expression modifications [120]. Epigenetic markers can be modified by environmental factors and may constitute an underlying biological mechanism affecting an individual's predisposition to almost all MetS components [121].

A recent, large epigenome-wide meta-analysis across 13 cohorts of the PACE consortium showed the effect of maternal exposure to tobacco smoke on newborn DNA methylation [31]. In an earlier study, we further showed that DNA methylation may mediate the effect of maternal smoking on the birth weight of the neonate. DNA methylation of only a few CpG sites explained 12–19% of the lower birth weight of the neonate of smoking mothers [122]. The mediating effect of DNA methylation between air pollution and cardiovascular phenotypes such as carotid intima–media thickness and blood pressure has been reported [123]. Besides, there is evidence that the effect of ambient air pollutants on blood glucose might be mediated by DNA methylation [69] and that there is a mediating effect of DNA methylation on other human complex traits such as inflammation and thrombosis [75] and neurodevelopmental disorders [124].

**Fig. 2:**
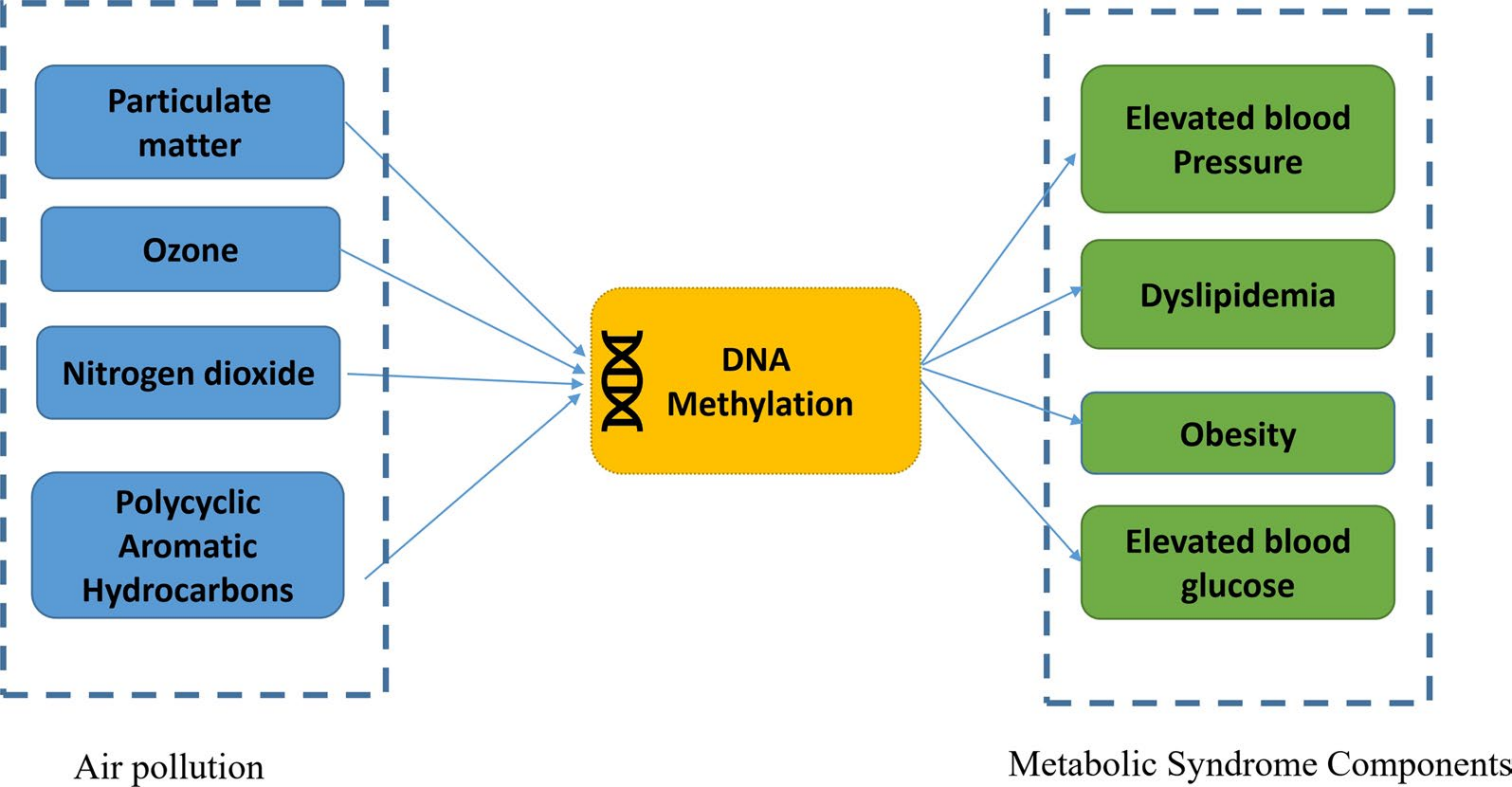
Hypothetical paths that might link criteria air pollutants exposures we discussed here to metabolic syndrome components through DNA methylation

Based on the combined evidence presented, we propose that alterations in DNA methylation may mediate adverse effects of environmental pollutants on MetS and its components (Fig. 2). We also summarized the overlapping genes reported in independent epigenetic studies of air pollution and MetS components in Additional file 1: Table S3. The direction of effects seems to be supportive of our main hypothesis, as for most of the sites ($\sim$98%) consistent direction of association of DNA methylation with air pollution and MetS is observed. Thus, DNA methylation could play an important role as an underlying explanatory mechanism. A few locus-specific example mechanisms from Additional file 1: Table S3 are discussed below:

Air pollution, including NO_2_ and PM, affects DNA methylation at the *NXN* gene, at which DNA methylation is also associated with obesity, glucose level, and blood pressure. This gene encodes nucleoredoxin, a regulator of Wnt signaling pathway and in utero embryonic development [125, 126]. Whether the mediating role of epigenetics occurs through effects on the embryonic environment and metabolic dysregulation proceeds with embryonic development is to be elucidated by further longitudinal studies. NO_2_ and PM also affect DNA methylation at the protein kinase C zeta gene (*PRKCZ*), at which differential methylation is also associated with obesity and fasting glucose levels. The involvement of this protein in the insulin signaling pathway [127] may describe another mechanism by which air pollution affects metabolic dysregulation, through epigenetic modifications. Another differentially methylated gene in relation to NO_2_ exposure is zinc finger MIZ domain-containing protein 1 (*ZMIZ1*), which is involved in androgen receptor signaling. This pathway has been studied in the context of sex differences in lipid profile and blood pressure [128–130]. Epigenetic regulation of this pathway may explain another mechanism by which air pollution affects metabolic complications.

Our study highlights the need and potential for large-scale genome-wide consortium meta-analysis on human samples with air pollution measures, DNA methylation levels, and MetS components being measured on the same samples preferably in the context of a prospective cohort design. Alternatively, there are large amounts of summary-level results available from very large-scale EWASs and air pollution measures and MetS components. One may think of integrating summary-level data from independent EWAS, gene expression studies, and epidemiologic studies to test the mediating effect of DNA methylation on the link between air pollution and MetS. Such analyses are based on summary-level results from large-scale epidemiological studies and hence may provide sufficient power to detect subtle mediating effects. However, methodological issues should be carefully considered [75, 120, 124]. Obviously, statistical significance is not equivalent to causality. An increasingly popular approach, Mendelian Randomization, uses genetically informed instrumental variables (IVs) to test whether the association of two traits is due to real causation. Mendelian Randomization may be extended to the field of epigenomics to dig into the causality of the associations [131]. While the statistical links between air pollution and MetS-related disorders, between air pollution and DNA methylation, and between DNA methylation and MetS have separately been established, here we integrated the evidence suggesting that the link between air pollution and Mets may be partly explained by DNA methylation. This sheds light on the design and conduct of future epidemiological and laboratory studies aiming to prevent MetS and its tragic consequences. This review provides evidence for the mediating role of DNA methylation explaining the effects of air pollution on the development of MetS. Further studies are necessary to provide the clinical impact of these associations.

## Epigenomics—beyond DNA methylation

Beyond DNA methylation, epigenetic regulation also includes histone modifications [132]. Upon the type and site of modification, this will lead to a decrease or increase in their nearby gene’s expression [133]. There is evidence of air pollution-induced histone modifications [134, 135], some of which are also observed in metabolic syndrome versus health [133, 136]. Studying these epigenetic marks together with their interplay with non-protein coding RNA transcripts (ncRNAs) will elucidate mediatory mechanisms from air pollution to metabolic syndrome [137]. Additional file 1: Table S3 is also suggestive of such interplay, where many ncRNA genes are among targets of DNA methylation in air pollution and MetS. Further research is required to elucidate the possible mediatory role of these mechanisms in linking air pollution to MetS, which is beyond the scope of this article.

## Limitations of the current state of the science

The available evidence on environment–methylation–disease patterns is mostly derived from blood methylation investigations. This choice of preferred tissue may be reasonable as typical exposure routes for air pollution are from inhalation, ingestion, and dermal exposure. However, one of the limitations of epigenetic investigations is the unavailability of data from disease/exposure-relevant tissues. On the other side, the tissue-specific pathogenesis of metabolic syndrome remains debated. When clarified and with increased availability of tissue-specific epigenetic data, deeper investigations of tissue-specific effects will be worthwhile.

## Future perspectives

Since there is no known level of air pollution exposure that is risk free, strategies to lessen daily exposures can always be effective. Global awareness of air pollution is increasing, and it is necessary to provide recommendations for reducing air pollution levels as well as human exposure to it. Public policy has a key role in reducing air pollution, and exposures can be mitigated by providing education to the community and support people to limit their exposure to harmful levels of air pollution. Recommendations in this regard include using filtering face masks, shifting from car and public transport to active transport, i.e., cycling or walking, maintaining vehicle ventilation, and avoiding rapid accelerations and decelerations. Moreover, household air pollution should be considered. Household exposure could be minimized by using clean fuels, enhancing household ventilation, and using efficient cookstoves [138–140].

In terms of epigenetic research, longitudinal measurements of DNA methylation alongside with different health outcomes could provide beneficial and informative resources [141]. Comparative analysis of methylation changes due to air pollution and/or other lifestyle habits can be of help. Moreover, it is essential to improve our understanding of the molecular mechanisms which mediate the effect of the environmental exposure on NCD prevalence and incidence. To ascertain the mediating molecular mechanisms from air pollution to MetS, a first step would be to investigate whether methylation alterations, in methylation regions that overlap between air pollution and MetS, result in actual changes in gene expression and corresponding protein levels. Then, we need to examine if these alterations eventually yield MetS or its components. The best strategy would be to study all three data levels of air pollution, DNA methylation, and MetS (components) in a single population, though integrative studies on different data levels could also provide clues to potential mechanisms by which different kinds of air pollutants contribute to morbidity and mortality. At the intervention side, assuming ROS causes epigenetic changes due to air pollution exposure, antioxidants and carotenoids can potentially reduce the harmful effects of air pollution on global epigenetic changes which leads to MetS and other types of diseases. Vitamin B supplementation is also observed to attenuate air pollution-induced epigenetic changes [1]. Targeted interventions based on air pollution-induced epigenetic modifications require a more complete and robust knowledge of the impacted loci and subsequently involved mechanisms.

## Conclusions

Current evidence supports the possible mediation of air pollution effects on metabolic syndrome by epigenetic regulation. This warrants further research to investigate the causality of associations with DNA methylation and the biological mechanisms involved.

## Supplementary Information

**Additional file 1**. Supplementary Tables 1–3.

## Data Availability

All papers reviewed and described in this manuscript are available online in the relevant publications.

## Abbreviations

ACE: Angiotensin-converting enzyme
AQI: Air quality index
ARG2: Arginase
CHARGE: Cohorts for Heart and Aging Research in Genomic Epidemiology
CO: Carbon monoxide
CpG: Cytosine–guanine dinucleotide
DALY: Deaths and disability-adjusted life years
DOHaD: Developmental Origins of Health and Disease
ET-1: Endothelin-1
EWAS: Epigenome-wide association study
GBD: Global Burden of Disease
IARC: International Agency for Research on Cancer
ICAM-1: Intercellular adhesion molecule 1
IUGR: Intrauterine growth restriction
IVs: Instrumental variables
MetS: Metabolic syndrome
NCDs: Non-communicable disease
NO2: Nitrogen dioxide
NOS2A: Nitric oxide synthase
O3: Ozone
PACE: Pregnancy And Childhood Epigenetics
PAHs: Polycyclic aromatic hydrocarbons
PM: Particulate matter
PM_2.5_: Particulate matter with a diameter ≤ 2.5 μm
SO2: Sulfur dioxide
