# Supplementary material for: DNA methylation: a potential mediator between air pollution and metabolic syndrome
Source: Clin Epigenetics. 2022 Jun 30;14:82. doi: 10.1186/s13148-022-01301-y (PMC9245491; doi:10.1186/s13148-022-01301-y)
Supplement: Supplementary file 1 — Additional file 1. Supplementary Tables 1–3. [file 13148_2022_1301_MOESM1_ESM.pdf]

Supplementary material

DNA methylation: a potential mediator between air pollution and metabolic syndrome

Parinaz Poursafa, Zoha Kamali, Eliza Fraszczyk, H. Marike Boezen, Ahmad Vaez, Harold Snieder

Supplementary Table 1. Page 2

Supplementary Table 2. Page 9

Supplementary Table 3. Page 10

Supplementary Table 1. Overview of the literature cited.

	Study	Ethnicity or Country	Study design/Study population	Methods/Tissue	Main Findings
AP and MetS	Wallwork et al [15]	Mixed	Longitudinal study/587 elderly male participants	–	Men living in areas of worse air quality ($PM_{2.5}$ levels) showed increased risk of metabolic dysfunctions.
	Kelishadi et al [17]	East Asian	Cross-sectional study/374 children	–	Exposure to air pollutants increases the risk of underlying MetS components.
	Jiang et al [18]	Chinese	Longitudinal/371 participants	–	Long-term exposure to traffic-related air pollution may contribute to the development or exacerbation of cardio-metabolic disorders.
	Wei et al [19]	–	Murine study	–	Breathing highly polluted air resulted in weight gain and cardiorespiratory and metabolic dysfunction.
	Clementi et al [26]	USA	Review/Firefighters	–	Exposure to particulate matter provokes pulmonary and systemic inflammation.
	Eze et al [27]	Swiss	Cohort/3,769 Adults	–	Exposure to ambient air pollutants is independently associated with risk of MetS and related chronic diseases.
	Brook et al [28]	China	Cross-sectional/65 Adults	–	Exposure to ambient air pollutants ($PM_{2.5}$) is associated with higher blood pressure and insuline resistance.
	Poursafa et al [29]	Iran (IR)	Nationwide study/1,413 children	–	Living in areas with higher air pollution leads to higher levels of MetS components including increased systolic blood pressure, fasting blood glucose, and triglycerides, as well as lower levels of HDL-C.
	Poursafa et al [30]	Iran (IR)	Cross-sectional/186 children	–	Exposure of children to PAHs is significantly associated with triglycerides and fasting blood glucose.
	Scinicariello et al [89]	USA	Cross-sectional/3,189 children	–	Urinary PAHs were associated with higher BMI, waist circumference, and obesity in children.
	Cantone et al [51]	Italy	Candidate gene/186 obese subjects	Bisulfite pyrosequencing/Blood	Daily PM_{10} exposure has an inverse association with DNA methylation of inflammatory genes in healthy overweight/obese subjects.

	Study	Ethnicity or Country	Study design/Study population	Methods/Tissue	Main Findings
AP and DNA methylation	Bind et al [56]	US Veterans	Cohort/704 elderly men	Bisulfite pyrosequencing/Blood	Traffic-related pollutants showed associations with fibrinogen, C-reactive protein, ICAM-1, and VCAM-1. DNA methylation status modified the effect, indicating a mediatory role for epigenetic states in conveying susceptibility to air pollution.
	Bind et al [57]	US Veterans	Longitudinal/777 elderly men	Bisulfite pyrosequencing/Blood	Air pollution (including $PM_{2.5}$) exposure was associated with a left shift in the lower tails of the *IFN-γ* and *ICAM-1* methylation distributions.
	Chi et al [60]	Mixed	Cross-sectional/1,207 participants	llumina Infinium HumanMethylation450 BeadChip/Blood monocytes	Long-term $PM_{2.5}$ exposure was significantly associated with five candidate CpGs, three of which mRNA expression of nearby genes (*ANKHD1*, *LGALS2*, and *ANKRD11*) were also significantly associated with $PM_{2.5}$ exposure.
	Plusquin et al [61]	UK	EWAS/1,235 individuals	Illumina InfiniumVR HumanMethylation 450K BeadChip/Cord blood	PM_{10} exposure is associated with locus-sepecific DNA methylation.
	Sayols-Baixeras et al [62]	Spain	EWAS/630 individuals	nfinium HumanMethylation450 BeadChip/Blood	Air pollution was significantly associated with 81 unique CpGs, the top two which were located in an intergenic region on chromosome 1 and in the *LRRC45* and *PXK* genes.
	Gondaliaa et al [63]	Mixed	EWAS/8,397 individuals	llumina 450K Infinium Methylation BeadChip/Blood	PM was significantly associated with DNA methylation at three CpG sites: *MATN4*, *ARPP21*, and *CFTR*.
	Rossnerova et al [65]	Czech Republic	Cross-sectional/200 children	Human Methylation 27K BeadChip/Blood	~36% of CpG sites had significantly different methylation levels. A total of 58 CpGs had >10% lower methylation levels in children from polluted area.
	Bellavia et al [67]	Canada	Cross-over trial/15 participants	Bisulfite PCR-Pyrosequencing/Blood	PM_{10} was significantly associated with lower methylation at *Alu* and *TLR4*, which were in turn associated with higher blood pressure.
	Chen et al [68]	China	Randomized, double-blind crossover trial/35 students	Bisulfite PCR and pyrosequencing/Blood	Short-term exposure to $PM_{2.5}$ was associated with rapid global DNA hypomethylation.

	Study	Ethnicity or Country	Study design/Study population	Methods/Tissue	Main Findings
AP and DNA methylation	Peng et al [69]	US Veterans	Longitudinal cohort/551 participants	Bisulfite PCR and pyrosequencing/Blood	Short- and medium-term $PM_{2.5}$ were associated with higher fasting blood glucose. Mediation analysis indicated that part of this association was mediated by *ICAM-1* promoter methylation.
	Saenen et al [71]	Belgium	Birth cohort/361 individuals	Bisulfite-PCR-pyrosequencing/Placenta	$PM_{2.5}$ exposure was significantly associated with lower placental *LEP* methylation.
	Salam et al [73]	Hispanic and non-Hispanic whites	Cohort/940 participants	Bisulfite PCR and pyrosequencing/Buccal cells	Short term $PM_{2.5}$ exposure affects inducible nitric oxide synthase (iNOS) methylation.
	Tarantini et al [74]	Italy	Cross-sectional/63 steel workers	Bisulfite-pyrosequencing/Blood	Particulate air pollutants were negatively correlated with DNA methylation at *NOS3* and *EDN1*. Mediation analysis then confirmed their hypomethylation as intermediate mechanism for PM-related coagulation effects.
	Bind et al [75]	US Veterans	Longitudinal/777 elderly men	Bisulfite pyrosequencing/Blood	Increase in air pollutants concentrations was significantly associated with *F3*, *ICAM-1*, and *TLR-2* hypomethylation, and *IFN-γ* and *IL- 6* hypermethylation. Ozone exposures (2-4 weeks) were negatively related to *ICAM-1* methylation and in turn to its protein level.
	Chen et al [76]	China	Longitudinal/30 retired COPD patients	Bisulfite-PCR and pyrosequencing assay/Buccal Samples	$PM_{2.5}$ exposure and was associated with hypomethylation of *NOS2A*.
	Shi et al [77]	–	Murine study and in situ investigation	Illumina HumanMethylation450K BeadChip/Human bronchial epithelial cells	$PM_{2.5}$ induced genome wide methylome and transcriptome alterations that could be involved in pulmonary toxicity and pathological process of respiratory disease.
	Tantoh et al [78]	Taiwanese	Cohort/948 participants	Illumina Infinium MethylationEPIC BeadChip/Blood	$PM_{2.5}$ and smoking were independently associated with hypomethylation of *AHRR* and *F2LR3*.

	Study	Ethnicity or Country	Study design/Study population	Methods/Tissue	Main Findings
AP and DNA methylation	Panni et al [79]	European	EWAS/2,956 participants from 3 cohorts	Illumina 450k BeadChip/Blood	Effect of $PM_{2.5}$ pollution on DNA methylation at 12 CpG sites (*ACVR2B-AS1*, *ACYP2*, *C1orf212*, *F2*, *MN1*, *MSGN1*, *NEURL4*, *NSMAF*, *NXN*, *SERBP1*, *TSPYL6*, and *ZMIZ1*).
	Dai et al [80]	Caucasians	EWAS/646 participants	Illumina 450k BeadChip/Blood	Long-term exposures to some $PM_{2.5}$ species (Fe, Ni, V), mostly combustion emitted particles, were associated with alterations in DNA methylation in immune response genes.
	Lee et al [81]	Korean	EWAS/100 individuals	Illumina 450k BeadChip/Blood	Long-term NO_2 exposure showed significant associations (FDR<0.05) with 45 CpGs, which were enriched in cardiovascular and respiratory diseases as well as inflammatory and immune responses.
	De Prins [82]	Belgium	Cross-sectional/48 adults	HPLC	Ambient air pollution is associated with lower global DNA methylation.
	Xia et al [83]	China	Longitudinal/43 students	Bisulfite-PCR and pyrosequencing assay/Blood	Acute exposure to ambient ozone was associated with higher blood pressure and with increased serum levels of angiotensin-converting enzyme (ACE) and endothelin-1 (ET-1) as biomarkers of blood pressure.
	Jiang et al [85]	China	Longitudinal/40 students	Bisulfite-PCR and pyrosequencing assay/Buccal Samples	Short term exposure to NO_2 is associated with DNA methylation of two respiratory function related genes, i.e. arginase (*ARG2*) hypermethylation and inducible nitric oxide synthase (*NOS2A*) hypomethylation.
	Gruzieva et al [86]	European and North American	EWAS/1,508 newborns from four birth cohort studies	Illumina 450k BeadChip/Blood	NO_2 exposure during pragnancy showed significant associations (FDR<0.05) with DNA methylation in three CpG sites (*LONP1*, *HIBADH*, and *SLC25A28*) of the newborns. NO_2 exposure also showed effects on *CAT* and *TPO* methylation as well as on their expression levels.
	Lichtenfels et al [87]	European	EWAS/1,017 subjects from the Lifelines cohort study	Illumina 450k BeadChip/Blood	Long-term NO_2 exposure showed significant associations with DNA methylation at seven CpG sites, two of which significantly mediated the association between NO_2 and lung function.
	Abraham et al [88]	Caucasian	EWAS/668 individuals	Illumina 450k BeadChip/Placenta	NO_2 exposure during the pregnancy showed significant associations (FDR<0.05) with placental DNA methylation of 2 CpGs located in the *ADORA2B* gene, whose expression is associated with hypoxia and pre-eclampsia.

	Study	Ethnicity or Country	Study design/Study population	Methods/Tissue	Main Findings
AP and DNA methylation	Alegría-Torres et al [90]	México	Cross-sectional/39 individuals	Bisulfite-PCR and pyrosequencing assay/Blood	PAHs were negatively associated with DNA methylation of theinterleukin 12,p53, and TNF-α gene promoters, as well as Alu sequences.
	Alvarado-Cruz et al [91]	México	Cross-sectional/108 individuals	Bisulfite-PCR and pyrosequencing assay/Blood	PM_{10} exposure is associated with hypermethylation of DNA-repair genes, and *LINE1*.
	Herbstman et al [92]	USA	Longitudinal/164 participants	Methylamp Global DNA Methylation Quantification Kit/Cord blood	Prenatal PAH exposure was associated with lower global methylation in umbilical cord white blood cells.
	Kim et al [93]	Korean	Cross-sectional/53 women	Bisulfite treatment and methylaion-specific PCR/Visceral adipose tissue	Lipophilic PAHs might contribute to the pathogenesis of insulin resistance through methylation-mediated suppression of the *IRS2* gene.
	Lin et al [94]	China	Cross-sectional/120 individuals	Illumina 450k BeadChip/Neural tissues	Mean methylation intensity of *PAX3* gene in fetal neural tissues was positively correlated with concentrations of PAH in maternal serum.
	Li et al [95]	Chinese	Cross-sectional/989 individuals	Illumina 450k BeadChip/Blood	PAHs showed a significant association with an epigenetic predictor of accelerated aging.
DNA methylation & MetS	Baccarelli et al [66]	US Veterans	Longitudinal/712 individuals	Bisulfite-PCR and pyrosequencing assay/Blood	Individuals with lower *LINE-1* methylation were at higher risk for incident ischemic heart disease and stroke, and for total mortality.
	Wang et al [70]	African-American	EWAS/112 individuals	HumanMethylation27 BeadChip from Illumina	Differential methylation at *SULF1* gene was observed and validated in hypertensive versus normotensive individuals.
	Godfrey et al [101]	Caucasian	Cross-sectional/239 children	Sequenom MassARRAY/Umbilical cord	Retinoid X receptor-a (*RXRA*) and endothelial nitric oxide synthase (*eNOS*) methylation had independent associations with childhood fat mass.

	Study	Ethnicity or Country	Study design/Study population	Methods/Tissue	Main Findings
DNA methylation and MetS	Küpers et al [102]	Mixed	EWAS/8,825 neonates from 24 birth cohorts	Meta-analysis	DNA methylation in neonatal blood showed genome-wide significant association with birthweight at 914 sites, with a range of -183 to 178g difference in birthweight per 10% increase in methylation.
	Luttmer et al [104]	Netherlands	Cross-sectional/738 individuals	Stable isotope dilution measurement of DNA 5-mc content by positive electrospray LC–MS/MS/Blood	Individuals with the metabolic syndrome had relative DNA hypomethylation compared to participants without the syndrome
	Akinyemiju et al [106]	African-American	EWAS/614 individuals	Illumina HumanMethylation450K Bead Chip/Blood	MetS showed consistent association with increased methylation in the *ABCG1* gene.
	Das et al [107]	European	EWAS/846 participants	Illumina HumanMethylation450K Bead Chip/Blood	Methylation at *CPT1A* was significantly associated with MetS. This finding was also replicated in African ancestry participants.
	Hidalgo et al [108]	USA	EWAS/837 participants	Illumina HumanMethylation450K Bead Chip/Blood	DNA methylation of *ABCG1* gene is associated with fasting insulin.
	Kulkarni et al [109]	Mexican-American	EWAS/850 individuals	Illumina HumanMethylation450K Bead Chip/Blood	DNA methylation at CpG sites mapping to three well-characterized genes (*TXNIP*, *ABCG1* and *SAMD12*) independently explained 7.8% of the heritability of type 2 diabetes.
	Richard et al [110]	European, African American, and Hispanic	EWAS/17,010 individuals	Illumina HumanMethylation450K Bead Chip/Blood	31 CpGs showed significant association with blood pressure, 13 of which were independently replicated. Six of the identified CpG sites also showed significant association with gene expression (*TSPAN2*, *SLC7A11*, *UNC93B1*, *CPT1A*, *PTMS*, and *LPCAT3*).

	Study	Ethnicity or Country	Study design/Study population	Methods/Tissue	Main Findings
DNA methylation and MetS	Huang et al [111]	European and African	EWAS/4,820 individuals	Meta-analysis	34 CpGs showed significant association with blood pressure. Six of the identified CpG sites also showed significant association (FDR < 0.05) with gene expression (*PHGDH*, *ABCG1*, *LMNA*, *RBPMS2*, and *SLC1A5*). Further investigation of these CpG sites in a meta-analysis of twin cohorts suggested that the majority of correlation between DNA methylation and blood pressure can be explained by shared environmental factors.
	Walaszczyk et al [112]	Netherlands	EWAS/200 individuals	Illumina 450k BeadChip/Blood	Of the 25 previously reported CpGs for type 2 diabetes, five CpGs (in *ABCG1*, *LOXL2*, *TXNIP*, *SLC1A5* and *SREBF1*) were replicated after adjustment for multiple comparisons.
	Demerath et al [113]	African-American	EWAS/4,474 individuals	Illumina 450k BeadChip/Blood	Adiposity traits are associated with DNA methylation at numerous CpG sites that replicate across studies despite variation in tissue type, ethnicity and analytic approaches.
	Ali et al [114]	European	EWAS/1,244 individuals	Illumina 450k BeadChip/Blood	DNA methylation at 3 loci (*SOCS3*, *ZNF771*, and *LIMD2*), was associated with body mass index, central obesity, fat depots, insulin responsiveness, and plasma lipids. Among these methylation at *SOCS3* was replicated and also showed gene expression association with MetS.
	Wahl et al [115]	European and Indian Asian	EWAS/5,387 individuals	Illumina 450k BeadChip/Blood	Body mass index, a key measure of adiposity, is associated with widespread changes in DNA methylation at 187 genetic loci.
	Wang et al [116]	African-American	EWAS/700 individuals	Illumina 450k BeadChip/Blood	76 obesity-related CpG sites were identified, 54 of which were validated. Sixteen CpG sites were associated with expression of 17 genes in cis, of which 5 genes displayed differential expression between obese cases and lean controls.

AP: air pollution, MetS: metabolic syndrome, EWAS: epigenome-wide association study, PM: particulate matter, PAHs: polycyclic aromatic hydrocarbons, PCR: polymerase chain reaction.
To generate this list, we searched PubMed database with appropriate keywords:
AP: "air pollution", PM, "particulate matter", "Ozone", "NO2", "PAHs", "Polycyclic aromatic hydrocarbons"
DNA methylation: "DNA methylation", EWAS
MetS: "metabolic syndrome", Obesity, "blood pressure", glucose, triglyceride, HDL
The search was done in late 2020.

Supplementary Table 2. Measurement methods of five major air pollutants used for the calculation of the air quality index (AQI) [2].

**Air pollutant**	**Measurement Method**	**Description**
Ozone (O_3)	- UV Photometric	Represents a variation of the ultraviolet (UV) photometric method, known as the "scrubberless" UV (SL-UV) method that specifies removal of O_3 from the sampled air.
	- Chemiluminescence	Utilizes Nitric Oxide to measure ozone in the atmosphere where the reaction between ambient ozone and NO produce light proportional to the ozone concentration.
Particulate Matter (PM)	Beta attenuation monitoring (BAM)	Absorption of beta radiation by solid particles extracted from air flow.
Carbon Monoxide (CO)	Nondispersive infrared (NDIR) technique	The NDIR technique is an automated and continuous method that is based on the specific absorption of infrared radiation by the CO molecule.
Sulfur Dioxide (SO_2)	Ultraviolet fluorescence	SO_2 molecules absorb UV light and become excited at one wavelength, then decay to a lower energy state emitting UV light at a different wavelength.
Nitrogen Dioxide (NO_2)	Chemiluminescence	Measurement of light produced by the gas-phase titration of nitric oxide and ozone. $NO + O_3 \rightarrow NO_2 + O_2$ + light emission.

Supplementary Table 3. The intersect of DNA methylation sites associated with air pollution (including PM, Ozone, NO_2, and PAHs) with those associated with MetS components (Obesity, blood pressure, glucose levels, triglycerides, or HDL-cholesterol). Data was retrieved from the MRC-IEU EWAS Catalog (http://www.ewascatalog.org), accessed 01/10/2022, and filtered based on a conservative p-value < $5x10^{-8}$.

Gene	Trait1	minP	Direction	PMID	Trait2	minP	Direction	PMID
LOC283050	Nitrogen dioxide exposure	4.70E-10	–	29410382	Maternal overweight/obesity	1.70E-10	–	29016858
RASA3	Air pollution exposure, Nitrogen dioxide exposure	4.80E-10	–	26731791, 29410382	Maternal overweight/obesity, fasting glucose, Triglycerides, Triglycerides to total lipids ratio in large LDL, Triglycerides to total lipids ratio in small HDL, Triglycerides to total lipids ratio in very large HDL	1.00E-10	–	29016858, 31197173
INPP5A	Air pollution exposure, Nitrogen dioxide exposure	4.80E-10	–	26731791, 29410382	Maternal overweight/obesity, fasting glucose, Ratio of diacylglycerol to triglycerides	4.00E-10	–	29016858, 31197173
CHD5	Nitrogen dioxide exposure	4.80E-10	–	29410382	Maternal overweight/obesity, 1-hour glucose, Ratio of diacylglycerol to triglycerides	1.60E-09	–	29016858, 33151971
SLC6A1	Nitrogen dioxide exposure	4.80E-10	–	29410382	Maternal overweight/obesity	4.10E-08	–	29016858
BCAR1	Nitrogen dioxide exposure	5.00E-10	–	29410382	Maternal overweight/obesity	6.10E-10	–	29016858

Gene	Trait1	minP	Direction	PMID	Trait2	minP	Direction	PMID
KLHL29	Air pollution exposure, Nitrogen dioxide exposure	5.00E-10	–	26731791, 29410382	Maternal overweight/obesity	1.90E-09	–	29016858
GPR84	Nitrogen dioxide exposure	5.00E-10	–	29410382	Maternal overweight/obesity	2.20E-09	–	29016858
AIRE	Nitrogen dioxide exposure	5.00E-10	–	29410382	Maternal overweight/obesity	3.30E-09	–	29016858
ANKRD2	Nitrogen dioxide exposure	5.00E-10	–	29410382	Maternal overweight/obesity	4.00E-08	–	29016858
ITGB2	Nitrogen dioxide exposure	5.10E-10	+	29410382	Maternal overweight/obesity	2.00E-11	+	29016858
GABBR1	Air pollution exposure, Nitrogen dioxide exposure	5.10E-10	–	26731791, 29410382	Maternal overweight/obesity, Triglycerides in large HDL	3.90E-11	–	29016858
ZFYVE28	Air pollution exposure, Nitrogen dioxide exposure	5.10E-10	–	26731791, 29410382	Maternal overweight/obesity, fasting glucose	4.20E-10	–	29016858, 31197173
RTKN	Nitrogen dioxide exposure	5.10E-10	–	29410382	Maternal overweight/obesity	1.20E-08	–	29016858
LCE6A	Nitrogen dioxide exposure	5.10E-10	–	29410382	Maternal overweight/obesity	1.20E-08	–	29016858
SH3BP2	Nitrogen dioxide exposure	5.10E-10	–	29410382	Maternal overweight/obesity	1.40E-08	–	29016858
GPR133	Nitrogen dioxide exposure	5.10E-10	–	29410382	Maternal overweight/obesity, Triglycerides in VLDL	2.30E-08	–	29016858
C4orf44	Nitrogen dioxide exposure	5.20E-10	–	29410382	Maternal overweight/obesity	1.60E-09	–	29016858

Gene	Trait1	minP	Direction	PMID	Trait2	minP	Direction	PMID
HIVEP3	Nitrogen dioxide exposure	5.20E-10	–	29410382	Maternal overweight/obesity, Diastolic Blood Pressure, Systolic Blood Pressure, fasting glucose, 1-hour glucose	3.00E-09	–	29016858, 29198723, 31197173, 33151971
VPS37C	Nitrogen dioxide exposure	5.20E-10	–	29410382	Maternal overweight/obesity	1.10E-08	–	29016858
ERAL1	Nitrogen dioxide exposure	5.30E-10	–	29410382	Maternal overweight/obesity	9.60E-12	–	29016858
RNF213	Nitrogen dioxide exposure	5.30E-10	–	29410382	Maternal overweight/obesity	5.60E-10	–	29016858
DLL4	Nitrogen dioxide exposure	5.30E-10	–	29410382	Maternal overweight/obesity	2.10E-08	–	29016858
NOS3	Nitrogen dioxide exposure	5.40E-10	–	29410382	Maternal overweight/obesity	3.70E-10	–	29016858
SMYD3	Air pollution exposure, Nitrogen dioxide exposure	5.40E-10	–	26731791, 29410382	Maternal overweight/obesity	6.30E-10	–	29016858
NOTCH4	Nitrogen dioxide exposure, Particulate matter <2.5um (PM2.5)	5.40E-10	–	29410382, 31148503	Maternal overweight/obesity, Triglycerides to total lipids ratio in very large HDL	7.70E-10	–	29016858
ZBTB7C	Nitrogen dioxide exposure	5.40E-10	–	29410382	Maternal overweight/obesity	8.00E-10	–	29016858

Gene	Trait1	minP	Direction	PMID	Trait2	minP	Direction	PMID
TSNARE1	Air pollution exposure, Nitrogen dioxide exposure	5.50E-10	–	26731791, 29410382	Maternal overweight/obesity, 1-hour glucose, Triglycerides to total lipids ratio in chylomicrons and XXL-VLDL, Triglycerides to total lipids ratio in very large HDL	8.80E-10	–	29016858, 33151971
SH3BP4	Nitrogen dioxide exposure	5.50E-10	–	29410382	Maternal overweight/obesity	3.50E-09	–	29016858
GALNTL2	Nitrogen dioxide exposure	5.50E-10	–	29410382	Maternal overweight/obesity	3.90E-09	–	29016858
GPC1	Nitrogen dioxide exposure	5.50E-10	–	29410382	Maternal overweight/obesity	8.90E-09	–	29016858
TSPAN9	Nitrogen dioxide exposure	5.50E-10	–	29410382	Maternal overweight/obesity	3.70E-08	–	29016858
CCDC21;SH3BGRL3	Nitrogen dioxide exposure	5.60E-10	–	29410382	Maternal overweight/obesity	2.00E-10	–	29016858
DNAJA4	Nitrogen dioxide exposure	5.60E-10	–	29410382	Maternal overweight/obesity	9.90E-10	–	29016858
SPERT	Nitrogen dioxide exposure	5.60E-10	–	29410382	Maternal overweight/obesity	3.10E-09	–	29016858
P2RX3	Nitrogen dioxide exposure	5.60E-10	–	29410382	Maternal overweight/obesity	3.40E-08	–	29016858
ABR	Air pollution exposure, Nitrogen dioxide exposure	5.70E-10	–	26731791, 29410382	Maternal overweight/obesity	8.70E-12	–	29016858

Gene	Trait1	minP	Direction	PMID	Trait2	minP	Direction	PMID
PRDM16	Air pollution exposure, Nitrogen dioxide exposure	5.70E-10	–	26731791, 29410382	Maternal overweight/obesity, Diastolic Blood Pressure, Systolic Blood Pressure, fasting glucose, Triglycerides to total lipids ratio in small VLDL	2.40E-11	–	29016858, 29198723, 31197173
KIAA1026	Air pollution exposure, Nitrogen dioxide exposure	5.70E-10	–	26731791, 29410382	Maternal overweight/obesity, fasting glucose	1.00E-10	–	29016858, 31197173
SH3PXD2A	Air pollution exposure, Nitrogen dioxide exposure	5.70E-10	–	26731791, 29410382	Maternal overweight/obesity	5.70E-10	–	29016858
TRIM26	Air pollution exposure, Nitrogen dioxide exposure	5.70E-10	–	26731791, 29410382	Maternal overweight/obesity, Triglycerides, Ratio of triglycerides to phosphoglycerides	9.30E-10	–	29016858, 31910897
NOTCH1	Nitrogen dioxide exposure	5.70E-10	–	29410382	Maternal overweight/obesity	8.40E-09	–	29016858
RILP	Nitrogen dioxide exposure	5.80E-10	–	29410382	Maternal overweight/obesity, Serum total triglycerides, Triglycerides in HDL, Triglycerides in medium HDL, Triglycerides in VLDL	9.80E-10	–	29016858
SLC8A1	Nitrogen dioxide exposure	5.80E-10	–	29410382	Maternal overweight/obesity	2.30E-09	–	29016858

Gene	Trait1	minP	Direction	PMID	Trait2	minP	Direction	PMID
ABHD6	Nitrogen dioxide exposure	5.80E-10	–	29410382	Maternal overweight/obesity	1.30E-08	–	29016858
PTPRN2	Air pollution exposure, Nitrogen dioxide exposure	5.90E-10	–	26731791, 29410382	Maternal overweight/obesity	2.00E-12	–	29016858
WNT2B	Air pollution exposure, Nitrogen dioxide exposure	5.90E-10	–	26731791, 29410382	Maternal overweight/obesity	4.60E-10	–	29016858
TSSC1	Air pollution exposure, Nitrogen dioxide exposure	5.90E-10	–	26731791, 29410382	Maternal overweight/obesity, fasting glucose	5.90E-10	–	29016858, 31197173
ROR1	Nitrogen dioxide exposure	5.90E-10	–	29410382	Maternal overweight/obesity	1.30E-08	–	29016858
HEPACAM	Nitrogen dioxide exposure	5.90E-10	–	29410382	Maternal overweight/obesity	2.10E-08	–	29016858
RASGRF2	Air pollution exposure, Nitrogen dioxide exposure	5.90E-10	–	26731791, 29410382	Maternal overweight/obesity	2.90E-08	–	29016858
CABP1	Nitrogen dioxide exposure	6.00E-10	–	29410382	Maternal overweight/obesity	2.20E-10	–	29016858
DAPK1	Nitrogen dioxide exposure	6.00E-10	–	29410382	Maternal overweight/obesity, Triglycerides to total lipids ratio in very large HDL	2.80E-09	–	29016858
COL11A2	Air pollution exposure, Nitrogen dioxide exposure	6.00E-10	–	26731791, 29410382	Maternal overweight/obesity	3.40E-09	–	29016858
FABP6	Nitrogen dioxide exposure	6.00E-10	–	29410382	Maternal overweight/obesity	2.60E-08	–	29016858
TMEM38A	Nitrogen dioxide exposure	6.10E-10	–	29410382	Maternal overweight/obesity	2.10E-08	–	29016858

Gene	Trait1	minP	Direction	PMID	Trait2	minP	Direction	PMID
KIRREL3	Nitrogen dioxide exposure	6.30E-10	–	29410382	Maternal overweight/obesity	1.30E-10	–	29016858
SEMA6A	Air pollution exposure, Nitrogen dioxide exposure	6.30E-10	–	26731791, 29410382	Maternal overweight/obesity, 1-hour glucose	5.90E-10	–	29016858, 33151971
SPTBN1	Air pollution exposure, Nitrogen dioxide exposure	6.30E-10	–	26731791, 29410382	Maternal overweight/obesity, Triglycerides to total lipids ratio in medium VLDL	1.80E-09	–	29016858
EPHA2	Nitrogen dioxide exposure	6.40E-10	–	29410382	Maternal overweight/obesity, Triglycerides in chylomicrons and extremely large VLDL	1.60E-13	–	29016858
CACNA2D3	Nitrogen dioxide exposure	6.50E-10	–	29410382	Maternal overweight/obesity	3.40E-09	–	29016858
ART1	Nitrogen dioxide exposure	6.50E-10	–	29410382	Maternal overweight/obesity	4.20E-08	–	29016858
LDB2	Nitrogen dioxide exposure	6.60E-10	–	29410382	Maternal overweight/obesity	6.30E-12	–	29016858
MCF2L	Air pollution exposure, Nitrogen dioxide exposure	6.60E-10	–	26731791, 29410382	Maternal overweight/obesity	3.10E-10	–	29016858
RD3	Nitrogen dioxide exposure	6.60E-10	–	29410382	Maternal overweight/obesity	2.90E-09	–	29016858
CHRNA7	Nitrogen dioxide exposure	6.60E-10	–	29410382	Maternal overweight/obesity, 1-hour glucose	3.20E-09	–	29016858, 33151971

Gene	Trait1	minP	Direction	PMID	Trait2	minP	Direction	PMID
CDC42BPB	Nitrogen dioxide exposure	6.60E-10	–	29410382	Maternal overweight/obesity, Systolic Blood Pressure, 1-hour glucose, Triglycerides in chylomicrons and extremely large VLDL	4.60E-09	–	29016858, 32520614, 33151971
CLCN1	Nitrogen dioxide exposure	6.70E-10	–	29410382	Maternal overweight/obesity	2.80E-10	–	29016858
ABCA4	Nitrogen dioxide exposure	6.80E-10	–	29410382	Maternal overweight/obesity, fasting glucose	2.30E-11	–	29016858, 31197173
C6orf25	Nitrogen dioxide exposure	6.80E-10	–	29410382	Maternal overweight/obesity	4.80E-10	–	29016858
LPCAT1	Nitrogen dioxide exposure	6.80E-10	–	29410382	Maternal overweight/obesity	9.40E-10	–	29016858
CASP9	Nitrogen dioxide exposure	6.80E-10	–	29410382	Maternal overweight/obesity	8.20E-09	–	29016858
ZAP70	Nitrogen dioxide exposure	6.80E-10	–	29410382	Maternal overweight/obesity, fasting glucose	3.50E-08	–	29016858, 31197173
FRMD1	Nitrogen dioxide exposure	6.90E-10	–	29410382	Maternal overweight/obesity	5.30E-10	–	29016858
COL6A3	Nitrogen dioxide exposure	7.00E-10	–	29410382	Maternal overweight/obesity	1.40E-10	–	29016858
KCNAB2	Air pollution exposure, Nitrogen dioxide exposure	7.00E-10	–	26731791, 29410382	Maternal overweight/obesity	1.90E-09	–	29016858
DEGS1	Nitrogen dioxide exposure	7.00E-10	+	29410382	Maternal overweight/obesity	5.80E-09	+	29016858

Gene	Trait1	minP	Direction	PMID	Trait2	minP	Direction	PMID
PLEKHG3	Nitrogen dioxide exposure	7.00E-10	–	29410382	Maternal overweight/obesity, fasting glucose	8.90E-09	–	29016858, 31197173
MEGF8	Nitrogen dioxide exposure	7.00E-10	–	29410382	Maternal overweight/obesity	1.20E-08	–	29016858
CTRB2	Nitrogen dioxide exposure	7.10E-10	–	29410382	Maternal overweight/obesity	2.10E-09	–	29016858
PDZRN3	Air pollution exposure, Nitrogen dioxide exposure	7.10E-10	–	26731791, 29410382	Maternal overweight/obesity	8.40E-09	–	29016858
CAPN5	Nitrogen dioxide exposure	7.10E-10	–	29410382	Maternal overweight/obesity	3.40E-08	–	29016858
PSORS1C1	Nitrogen dioxide exposure	7.20E-10	–	29410382	Maternal overweight/obesity	2.00E-09	–	29016858
PDGFRB	Nitrogen dioxide exposure	7.20E-10	–	29410382	Maternal overweight/obesity	5.60E-09	–	29016858
NTN1	Nitrogen dioxide exposure	7.20E-10	–	29410382	Maternal overweight/obesity	9.00E-09	–	29016858
B4GALNT3	Nitrogen dioxide exposure	7.20E-10	–	29410382	Maternal overweight/obesity	1.60E-08	–	29016858
TRIM29	Nitrogen dioxide exposure	7.20E-10	–	29410382	Maternal overweight/obesity	3.70E-08	–	29016858
ADAM11	Nitrogen dioxide exposure	7.20E-10	–	29410382	Maternal overweight/obesity	4.50E-08	–	29016858
MICAL2	Nitrogen dioxide exposure	7.30E-10	–	29410382	Maternal overweight/obesity	7.50E-11	–	29016858
SHANK2	Nitrogen dioxide exposure	7.30E-10	–	29410382	Maternal overweight/obesity	7.20E-10	–	29016858
RPH3A	Air pollution exposure, Nitrogen dioxide exposure	7.30E-10	–	26731791, 29410382	Maternal overweight/obesity	2.70E-09	–	29016858

Gene	Trait1	minP	Direction	PMID	Trait2	minP	Direction	PMID
DIP2C	Nitrogen dioxide exposure	7.40E-10	–	29410382	Maternal overweight/obesity, Triglycerides, Glucose, Triglycerides in medium HDL, Triglycerides to total lipids ratio in medium VLDL	3.20E-12	–	29016858, 31910897
RAPGEF4	Nitrogen dioxide exposure	7.40E-10	–	29410382	Maternal overweight/obesity	1.10E-09	–	29016858
BAHCC1	Nitrogen dioxide exposure	7.40E-10	+	29410382	Maternal overweight/obesity, fasting glucose	1.40E-09	–	29016858, 31197173
PPT2;PRRT1	Nitrogen dioxide exposure	7.50E-10	–	29410382	Maternal overweight/obesity	4.30E-11	–	29016858
ZFPM1	Air pollution exposure, Nitrogen dioxide exposure	7.50E-10	–	26731791, 29410382	Maternal overweight/obesity	9.40E-10	–	29016858
RPS6KA2	Nitrogen dioxide exposure	7.50E-10	–	29410382	Serum triglycerides, Maternal overweight/obesity, Triglycerides to total lipids ratio in large HDL	2.10E-09	–	28213390, 29016858
ALDOC	Nitrogen dioxide exposure	7.60E-10	–	29410382	Maternal overweight/obesity	3.20E-10	–	29016858
TEX101	Nitrogen dioxide exposure	7.60E-10	–	29410382	Maternal overweight/obesity	6.20E-10	–	29016858
TRPM8	Nitrogen dioxide exposure	7.60E-10	–	29410382	Maternal overweight/obesity	1.50E-08	–	29016858
MFSD10	Nitrogen dioxide exposure	7.60E-10	+	29410382	Maternal overweight/obesity	3.10E-08	+	29016858

Gene	Trait1	minP	Direction	PMID	Trait2	minP	Direction	PMID
KLHL30	Nitrogen dioxide exposure	7.70E-10	–	29410382	Maternal overweight/obesity	7.50E-10	–	29016858
CORO1A	Nitrogen dioxide exposure	7.70E-10	+	29410382	Maternal overweight/obesity, Triglycerides in small LDL, Triglycerides to total lipids ratio in small LDL	7.80E-10	+	29016858
NPTX2	Air pollution exposure, Nitrogen dioxide exposure	7.70E-10	–	26731791, 29410382	Maternal overweight/obesity	1.90E-09	–	29016858
MAD1L1	Air pollution exposure, Nitrogen dioxide exposure, Particulate matter air pollution	7.80E-10	–	26731791, 29410382, 32484729	Maternal overweight/obesity, fasting glucose, 1-hour glucose, 2-hour glucose, Ratio of diacylglycerol to triglycerides, Triglycerides in large HDL, Triglycerides in small LDL	1.40E-11	–	29016858, 31197173, 33151971
AGRN	Air pollution exposure, Nitrogen dioxide exposure	7.80E-10	–	26731791, 29410382	Maternal overweight/obesity	1.70E-11	–	29016858
KIAA0802	Nitrogen dioxide exposure	7.80E-10	–	29410382	Maternal overweight/obesity	1.60E-09	–	29016858
PDE2A	Air pollution exposure, Nitrogen dioxide exposure	7.80E-10	–	26731791, 29410382	Maternal overweight/obesity, 1-hour glucose	2.80E-09	–	29016858, 33151971

Gene	Trait1	minP	Direction	PMID	Trait2	minP	Direction	PMID
FAM53A	Nitrogen dioxide exposure	7.80E-10	–	29410382	Maternal overweight/obesity, Ratio of triglycerides to phosphoglycerides, Triglycerides to total lipids ratio in large HDL, Triglycerides to total lipids ratio in medium HDL, Triglycerides to total lipids ratio in small HDL, Triglycerides to total lipids ratio in very large HDL	4.70E-09	–	29016858
APOA4	Air pollution exposure, Nitrogen dioxide exposure	7.80E-10	–	26731791, 29410382	Maternal overweight/obesity	8.00E-09	–	29016858
HLA-DQB2	Nitrogen dioxide exposure	7.80E-10	–	29410382	Maternal overweight/obesity	3.00E-08	–	29016858
FYB	Nitrogen dioxide exposure	7.90E-10	+	29410382	Maternal overweight/obesity	9.60E-13	+	29016858
FZD3	Nitrogen dioxide exposure	7.90E-10	–	29410382	Maternal overweight/obesity	5.80E-09	–	29016858
CD80	Nitrogen dioxide exposure	7.90E-10	–	29410382	Maternal overweight/obesity	1.60E-08	–	29016858
PSORS1C1;PSORS1C2	Nitrogen dioxide exposure	7.90E-10	–	29410382	Maternal overweight/obesity	1.80E-08	–	29016858
ANKRD9	Nitrogen dioxide exposure	7.90E-10	–	29410382	Maternal overweight/obesity	4.00E-08	–	29016858
NXNL1	Nitrogen dioxide exposure	8.00E-10	–	29410382	Maternal overweight/obesity	1.60E-08	–	29016858

Gene	Trait1	minP	Direction	PMID	Trait2	minP	Direction	PMID
INF2	Nitrogen dioxide exposure	8.10E-10	–	29410382	Maternal overweight/obesity	5.00E-12	–	29016858
CALML6	Nitrogen dioxide exposure	8.10E-10	–	29410382	Maternal overweight/obesity	5.90E-11	–	29016858
PRSS36	Air pollution exposure, Nitrogen dioxide exposure	8.10E-10	–	26731791, 29410382	Maternal overweight/obesity	4.20E-09	–	29016858
MGST2	Nitrogen dioxide exposure	8.10E-10	–	29410382	Maternal overweight/obesity	7.30E-09	–	29016858
ENPP7	Nitrogen dioxide exposure	8.10E-10	–	29410382	Maternal overweight/obesity	7.90E-09	–	29016858
MIR548I2	Nitrogen dioxide exposure	8.10E-10	–	29410382	Maternal overweight/obesity	2.20E-08	–	29016858
KRBA2	Nitrogen dioxide exposure	8.20E-10	–	29410382	Maternal overweight/obesity	2.20E-11	–	29016858
NUAK1	Nitrogen dioxide exposure	8.20E-10	–	29410382	Maternal overweight/obesity	3.40E-09	–	29016858
APBA2	Nitrogen dioxide exposure	8.20E-10	–	29410382	Maternal overweight/obesity	3.80E-09	–	29016858
CDK5	Nitrogen dioxide exposure	8.20E-10	–	29410382	Maternal overweight/obesity	3.50E-08	–	29016858
SMYD1	Nitrogen dioxide exposure	8.30E-10	–	29410382	Maternal overweight/obesity	4.40E-10	–	29016858
SLC6A19	Nitrogen dioxide exposure	8.30E-10	–	29410382	Maternal overweight/obesity	9.80E-10	–	29016858
GNAO1	Nitrogen dioxide exposure	8.30E-10	–	29410382	Maternal overweight/obesity	1.30E-08	–	29016858
GPT	Nitrogen dioxide exposure	8.30E-10	–	29410382	Maternal overweight/obesity	1.30E-08	–	29016858
IL17REL	Nitrogen dioxide exposure	8.30E-10	–	29410382	Maternal overweight/obesity	1.40E-08	–	29016858

Gene	Trait1	minP	Direction	PMID	Trait2	minP	Direction	PMID
C11orf16	Nitrogen dioxide exposure	8.30E-10	–	29410382	Maternal overweight/obesity	1.80E-08	–	29016858
GLI2	Nitrogen dioxide exposure	8.30E-10	–	29410382	Maternal overweight/obesity	2.80E-08	–	29016858
SLC4A7	Nitrogen dioxide exposure	8.60E-10	–	29410382	Maternal overweight/obesity	1.90E-13	–	29016858
PFKP	Nitrogen dioxide exposure	8.60E-10	–	29410382	Maternal overweight/obesity, Triglycerides in small HDL	6.60E-09	–	29016858
SNX29	Nitrogen dioxide exposure	8.60E-10	–	29410382	Maternal overweight/obesity, Triglycerides	9.30E-09	–	29016858, 31910897
SORBS2	Nitrogen dioxide exposure	8.60E-10	–	29410382	Maternal overweight/obesity	4.20E-08	–	29016858
ALDH3A1	Nitrogen dioxide exposure	8.70E-10	–	29410382	Maternal overweight/obesity	7.40E-09	–	29016858
C1orf177	Nitrogen dioxide exposure	8.70E-10	–	29410382	Maternal overweight/obesity, fasting glucose	1.80E-08	–	29016858, 31197173
CCHCR1	Nitrogen dioxide exposure	8.70E-10	–	29410382	Maternal overweight/obesity	2.50E-08	–	29016858
OBSCN	Nitrogen dioxide exposure	8.80E-10	–	29410382	Maternal overweight/obesity, Triglycerides in very large HDL	1.70E-09	–	29016858
SDK2	Nitrogen dioxide exposure	8.90E-10	–	29410382	Maternal overweight/obesity	3.50E-11	–	29016858
ABCA3	Air pollution exposure, Nitrogen dioxide exposure	8.90E-10	–	26731791, 29410382	Maternal overweight/obesity	3.60E-09	–	29016858

Gene	Trait1	minP	Direction	PMID	Trait2	minP	Direction	PMID
CACNA2D4	Nitrogen dioxide exposure	8.90E-10	–	29410382	Maternal overweight/obesity	9.40E-09	–	29016858
BCL11A	Air pollution exposure, Prenatal NO2 exposure, Nitrogen dioxide exposure	9.00E-10	–	26731791, 27448387, 29410382	Maternal overweight/obesity	2.60E-09	–	29016858
FFAR1	Nitrogen dioxide exposure	9.10E-10	–	29410382	Maternal overweight/obesity	2.70E-10	–	29016858
ADAMTS2	Air pollution exposure, Nitrogen dioxide exposure	9.10E-10	–	26731791, 29410382	Maternal overweight/obesity	7.90E-09	–	29016858
MSLNL	Nitrogen dioxide exposure	9.10E-10	–	29410382	Maternal overweight/obesity	2.20E-08	–	29016858
NECAB3;C20orf134	Nitrogen dioxide exposure	9.20E-10	–	29410382	Maternal overweight/obesity	6.40E-10	–	29016858
GPR152	Nitrogen dioxide exposure	9.20E-10	–	29410382	Maternal overweight/obesity	1.50E-08	–	29016858
KIF3C	Nitrogen dioxide exposure	9.30E-10	–	29410382	Maternal overweight/obesity	4.50E-10	–	29016858
EFCAB4B	Nitrogen dioxide exposure	9.30E-10	–	29410382	Maternal overweight/obesity	1.30E-08	–	29016858
RAB11FIP3	Air pollution exposure, Nitrogen dioxide exposure	9.30E-10	–	26731791, 29410382	Maternal overweight/obesity, fasting glucose, Diastolic blood pressure	2.40E-08	–	29016858, 31197173
SCN4B	Nitrogen dioxide exposure	9.40E-10	–	29410382	Maternal overweight/obesity, Ratio of triglycerides to phosphoglycerides,	4.20E-08	–	29016858

Gene	Trait1	minP	Direction	PMID	Trait2	minP	Direction	PMID
					Triglycerides in small VLDL			
MAG	Nitrogen dioxide exposure	9.50E-10	–	29410382	Maternal overweight/obesity	1.40E-09	–	29016858
PIK3C2B	Nitrogen dioxide exposure	9.50E-10	–	29410382	Maternal overweight/obesity	2.70E-08	–	29016858
PKLR	Nitrogen dioxide exposure	9.50E-10	–	29410382	Maternal overweight/obesity	4.40E-08	–	29016858
TNN	Nitrogen dioxide exposure	9.60E-10	–	29410382	Maternal overweight/obesity	2.80E-08	–	29016858
ZSCAN10	Air pollution exposure, Nitrogen dioxide exposure	9.70E-10	–	26731791, 29410382	Maternal overweight/obesity	4.90E-08	–	29016858
KRT13	Nitrogen dioxide exposure	9.80E-10	–	29410382	Maternal overweight/obesity, 1-hour glucose	1.50E-11	–	29016858, 33151971
IL17RC	Nitrogen dioxide exposure	9.80E-10	–	29410382	Maternal overweight/obesity	2.60E-09	–	29016858
OBSL1	Air pollution exposure, Nitrogen dioxide exposure	9.90E-10	–	26731791, 29410382	Maternal overweight/obesity	4.60E-09	–	29016858
RAPSN	Nitrogen dioxide exposure	1.00E-09	–	29410382	Maternal overweight/obesity	1.00E-10	–	29016858
WBSCR17	Nitrogen dioxide exposure	1.00E-09	–	29410382	Maternal overweight/obesity	1.20E-09	–	29016858
LOC389333	Nitrogen dioxide exposure	1.00E-09	–	29410382	Maternal overweight/obesity	1.60E-09	–	29016858

Gene	Trait1	minP	Direction	PMID	Trait2	minP	Direction	PMID
CHD3;SCARNA21	Nitrogen dioxide exposure	1.00E-09	–	29410382	Maternal overweight/obesity	2.50E-09	–	29016858
RTEL1	Nitrogen dioxide exposure	1.00E-09	–	29410382	Maternal overweight/obesity	2.90E-09	–	29016858
DLG2	Nitrogen dioxide exposure	1.00E-09	–	29410382	Maternal overweight/obesity	4.30E-09	–	29016858
TRAM2	Nitrogen dioxide exposure	1.00E-09	–	29410382	Maternal overweight/obesity, Ratio of diacylglycerol to triglycerides	6.60E-09	–	29016858
FGFR1	Nitrogen dioxide exposure	1.00E-09	–	29410382	Maternal overweight/obesity, Diastolic blood pressure	9.90E-09	–	29016858
KDM2B	Nitrogen dioxide exposure	1.00E-09	–	29410382	Maternal overweight/obesity, Serum total triglycerides, Triglycerides, Triglycerides in medium VLDL, Triglycerides in small VLDL, Triglycerides in VLDL	1.20E-08	–	29016858
EHMT2	Air pollution exposure, Nitrogen dioxide exposure	1.00E-09	–	26731791, 29410382	Maternal overweight/obesity	1.60E-08	–	29016858
MOCS1	Nitrogen dioxide exposure	1.00E-09	–	29410382	Maternal overweight/obesity	1.70E-08	–	29016858
ACRBP	Nitrogen dioxide exposure	1.00E-09	–	29410382	Maternal overweight/obesity	1.90E-08	–	29016858

Gene	Trait1	minP	Direction	PMID	Trait2	minP	Direction	PMID
SLCO3A1	Nitrogen dioxide exposure	1.10E-09	–	29410382	Maternal overweight/obesity, fasting glucose, Triglycerides in small VLDL	6.20E-13	–	29016858, 31197173
EPB49	Nitrogen dioxide exposure	1.10E-09	–	29410382	Maternal overweight/obesity	5.20E-12	–	29016858
MRVI1	Nitrogen dioxide exposure	1.10E-09	–	29410382	Maternal overweight/obesity	9.70E-11	–	29016858
GPR37L1	Nitrogen dioxide exposure	1.10E-09	–	29410382	Maternal overweight/obesity	1.40E-10	–	29016858
MYT1L	Air pollution exposure, Nitrogen dioxide exposure, Particulate matter air pollution	1.10E-09	–	26731791, 29410382, 32484729	Maternal overweight/obesity	8.50E-10	–	29016858
KCNJ6	Nitrogen dioxide exposure	1.10E-09	–	29410382	Maternal overweight/obesity	2.30E-09	–	29016858
HIP1R	Nitrogen dioxide exposure	1.10E-09	–	29410382	Maternal overweight/obesity	2.40E-09	–	29016858
HRNBP3	Nitrogen dioxide exposure, Particulate matter air pollution	1.10E-09	–	29410382, 32484729	Maternal overweight/obesity	2.60E-09	–	29016858
CACNA1C	Air pollution exposure, Nitrogen dioxide exposure	1.10E-09	–	26731791, 29410382	Maternal overweight/obesity, Triglycerides	3.10E-09	–	29016858, 31910897
C10orf116	Nitrogen dioxide exposure	1.10E-09	–	29410382	Maternal overweight/obesity	6.20E-09	–	29016858
CAPN13	Nitrogen dioxide exposure	1.10E-09	–	29410382	Maternal overweight/obesity	7.20E-09	–	29016858
CDGAP	Nitrogen dioxide exposure	1.10E-09	–	29410382	Maternal overweight/obesity	8.70E-09	–	29016858

Gene	Trait1	minP	Direction	PMID	Trait2	minP	Direction	PMID
PTPRS	Nitrogen dioxide exposure	1.10E-09	–	29410382	Maternal overweight/obesity	9.50E-09	–	29016858
PLXNA2	Nitrogen dioxide exposure	1.10E-09	–	29410382	Maternal overweight/obesity, Glucose	1.50E-08	–	29016858
CAMSAP1	Nitrogen dioxide exposure	1.10E-09	–	29410382	Maternal overweight/obesity	2.30E-08	–	29016858
STOX2	Nitrogen dioxide exposure	1.10E-09	–	29410382	Maternal overweight/obesity	2.30E-08	–	29016858
POU6F1	Nitrogen dioxide exposure	1.10E-09	–	29410382	Maternal overweight/obesity	2.90E-08	–	29016858
SERINC2	Air pollution exposure, Nitrogen dioxide exposure	1.10E-09	+	26731791, 29410382	Maternal overweight/obesity	4.30E-08	+	29016858
SEPT9	Nitrogen dioxide exposure	1.20E-09	–	29410382	Maternal overweight/obesity	5.80E-11	–	29016858
IGFN1	Nitrogen dioxide exposure	1.20E-09	–	29410382	Maternal overweight/obesity	6.50E-11	–	29016858
C6orf10	Nitrogen dioxide exposure	1.20E-09	–	29410382	Maternal overweight/obesity	1.60E-10	–	29016858
TNXB	Air pollution exposure, Nitrogen dioxide exposure	1.20E-09	–	26731791, 29410382	Maternal overweight/obesity, Glucose, Triglycerides to total lipids ratio in chylomicrons and XXL-VLDL	2.40E-10	–	29016858
TAP1	Nitrogen dioxide exposure	1.20E-09	–	29410382	Maternal overweight/obesity	4.40E-10	–	29016858
PPYR1	Nitrogen dioxide exposure	1.20E-09	–	29410382	Maternal overweight/obesity	8.20E-10	–	29016858

Gene	Trait1	minP	Direction	PMID	Trait2	minP	Direction	PMID
FAM83A	Nitrogen dioxide exposure	1.20E-09	–	29410382	Maternal overweight/obesity, Triglycerides to total lipids ratio in large LDL, Triglycerides to total lipids ratio in medium LDL, Triglycerides to total lipids ratio in small LDL	1.00E-09	–	29016858
FLJ45079	Nitrogen dioxide exposure	1.20E-09	–	29410382	Maternal overweight/obesity	2.20E-09	–	29016858
SRC	Nitrogen dioxide exposure	1.20E-09	–	29410382	Maternal overweight/obesity	2.30E-09	–	29016858
HOMER2	Nitrogen dioxide exposure	1.20E-09	–	29410382	Maternal overweight/obesity, Triglycerides in HDL, Triglycerides in IDL, Triglycerides in medium HDL, Triglycerides in small HDL, Triglycerides in small LDL	2.80E-09	–	29016858
ASB2	Nitrogen dioxide exposure	1.20E-09	–	29410382	Maternal overweight/obesity	5.10E-09	–	29016858
C10orf90	Nitrogen dioxide exposure	1.20E-09	–	29410382	Maternal overweight/obesity	7.50E-09	–	29016858
C1orf175	Air pollution exposure, Nitrogen dioxide exposure	1.20E-09	–	26731791, 29410382	Maternal overweight/obesity	1.20E-08	–	29016858

Gene	Trait1	minP	Direction	PMID	Trait2	minP	Direction	PMID
RICH2	Air pollution exposure, Nitrogen dioxide exposure	1.20E-09	–	26731791, 29410382	Maternal overweight/obesity	1.50E-08	–	29016858
SIPA1L2	Nitrogen dioxide exposure	1.20E-09	–	29410382	Maternal overweight/obesity, Triglycerides in very small VLDL	1.80E-08	–	29016858
THSD4	Nitrogen dioxide exposure	1.20E-09	–	29410382	Maternal overweight/obesity	2.50E-08	–	29016858
GALNT9;LOC100130238	Nitrogen dioxide exposure	1.20E-09	–	29410382	Maternal overweight/obesity, Glucose	2.70E-08	–	29016858
FAM176A	Nitrogen dioxide exposure	1.20E-09	–	29410382	Maternal overweight/obesity	3.30E-08	–	29016858
MDFI	Nitrogen dioxide exposure	1.20E-09	–	29410382	Maternal overweight/obesity	3.50E-08	–	29016858
D2HGDH	Nitrogen dioxide exposure	1.20E-09	–	29410382	Maternal overweight/obesity, Ratio of diacylglycerol to triglycerides	3.90E-08	–	29016858
C1orf210	Nitrogen dioxide exposure	1.20E-09	–	29410382	Maternal overweight/obesity	4.10E-08	–	29016858
NLRP10	Nitrogen dioxide exposure	1.20E-09	–	29410382	Maternal overweight/obesity	4.90E-08	–	29016858
AQP1	Nitrogen dioxide exposure	1.30E-09	–	29410382	Maternal overweight/obesity	4.30E-11	–	29016858
MAML3	Nitrogen dioxide exposure	1.30E-09	–	29410382	Maternal overweight/obesity, 1-hour glucose, Triglycerides to total lipids ratio in	4.80E-11	–	29016858, 33151971

Gene	Trait1	minP	Direction	PMID	Trait2	minP	Direction	PMID
					chylomicrons and XXL-VLDL			
EIF4G1	Nitrogen dioxide exposure	1.30E-09	–	29410382	Maternal overweight/obesity	5.10E-11	–	29016858
ATP10A	Air pollution exposure, Nitrogen dioxide exposure	1.30E-09	–	26731791, 29410382	Maternal overweight/obesity	1.80E-10	–	29016858
MRAS	Nitrogen dioxide exposure	1.30E-09	–	29410382	Maternal overweight/obesity	3.00E-10	–	29016858
GRM6	Nitrogen dioxide exposure	1.30E-09	–	29410382	Maternal overweight/obesity	6.70E-10	–	29016858
CDK18	Air pollution exposure, Nitrogen dioxide exposure	1.30E-09	–	26731791, 29410382	Maternal overweight/obesity	7.50E-10	–	29016858
GNG7	Air pollution exposure, Nitrogen dioxide exposure	1.30E-09	–	26731791, 29410382	Maternal overweight/obesity, Triglycerides in large HDL, Triglycerides to total lipids ratio in very large VLDL	9.00E-10	–	29016858
NCRNA00171	Nitrogen dioxide exposure	1.30E-09	–	29410382	Maternal overweight/obesity	1.40E-09	–	29016858
ZNF593	Nitrogen dioxide exposure	1.30E-09	–	29410382	Maternal overweight/obesity	1.80E-09	–	29016858
TACC2	Air pollution exposure, Nitrogen dioxide exposure	1.30E-09	–	26731791, 29410382	Maternal overweight/obesity	2.10E-09	–	29016858

Gene	Trait1	minP	Direction	PMID	Trait2	minP	Direction	PMID
UST	Nitrogen dioxide exposure	1.30E-09	–	29410382	Maternal overweight/obesity	3.40E-09	–	29016858
LY6G6C	Nitrogen dioxide exposure	1.30E-09	–	29410382	Maternal overweight/obesity	4.80E-09	–	29016858
ARHGEF3	Nitrogen dioxide exposure	1.30E-09	–	29410382	Maternal overweight/obesity	5.70E-09	–	29016858
RNASE7	Nitrogen dioxide exposure	1.30E-09	–	29410382	Maternal overweight/obesity	6.10E-09	–	29016858
MX1	Nitrogen dioxide exposure	1.30E-09	–	29410382	Maternal overweight/obesity	9.80E-09	–	29016858
DOK7	Nitrogen dioxide exposure	1.30E-09	–	29410382	Maternal overweight/obesity, Triglycerides to total lipids ratio in very large HDL	1.30E-08	–	29016858
C14orf181	Nitrogen dioxide exposure	1.30E-09	–	29410382	Maternal overweight/obesity	1.90E-08	–	29016858
NEURL	Nitrogen dioxide exposure	1.30E-09	–	29410382	Maternal overweight/obesity	2.30E-08	–	29016858
ST3GAL6	Nitrogen dioxide exposure	1.30E-09	–	29410382	Maternal overweight/obesity, 1-hour glucose	2.50E-08	–	29016858, 33151971
SMAD7	Nitrogen dioxide exposure	1.30E-09	–	29410382	Maternal overweight/obesity, fasting glucose	3.00E-08	–	29016858, 31197173
PRKAR2A	Nitrogen dioxide exposure	1.30E-09	–	29410382	Maternal overweight/obesity	3.10E-08	+	29016858
RBP3	Nitrogen dioxide exposure	1.30E-09	–	29410382	Maternal overweight/obesity	4.10E-08	–	29016858
RHPN1	Nitrogen dioxide exposure	1.30E-09	–	29410382	Maternal overweight/obesity, Ratio of	4.30E-08	–	29016858

Gene	Trait1	minP	Direction	PMID	Trait2	minP	Direction	PMID
					diacylglycerol to triglycerides			
PDE6B	Nitrogen dioxide exposure	1.30E-09	–	29410382	Maternal overweight/obesity	4.30E-08	–	29016858
SLIT3	Nitrogen dioxide exposure	1.40E-09	–	29410382	Maternal overweight/obesity, Triglycerides in chylomicrons and extremely large VLDL	2.20E-11	–	29016858
MFSD2B	Nitrogen dioxide exposure	1.40E-09	–	29410382	Maternal overweight/obesity	6.30E-11	–	29016858
TRIM2	Nitrogen dioxide exposure	1.40E-09	–	29410382	Maternal overweight/obesity	4.20E-10	–	29016858
FBXW12	Nitrogen dioxide exposure	1.40E-09	–	29410382	Maternal overweight/obesity	4.50E-10	–	29016858
PLCH2	Nitrogen dioxide exposure	1.40E-09	–	29410382	Maternal overweight/obesity, Glucose	1.00E-09	–	29016858
ST14	Nitrogen dioxide exposure	1.40E-09	–	29410382	Maternal overweight/obesity, Triglycerides to total lipids ratio in IDL, Triglycerides to total lipids ratio in small VLDL	1.90E-09	–	29016858
FLJ10357	Nitrogen dioxide exposure	1.40E-09	–	29410382	Maternal overweight/obesity	1.90E-09	–	29016858
NTM	Air pollution exposure, Nitrogen dioxide exposure	1.40E-09	–	26731791, 29410382	Maternal overweight/obesity, fasting glucose	2.00E-09	–	29016858, 31197173

Gene	Trait1	minP	Direction	PMID	Trait2	minP	Direction	PMID
MGMT	Nitrogen dioxide exposure	1.40E-09	–	29410382	Maternal overweight/obesity, fasting glucose	2.30E-09	–	29016858, 31197173
TUB	Nitrogen dioxide exposure	1.40E-09	–	29410382	Maternal overweight/obesity	3.10E-09	–	29016858
ADSSL1	Nitrogen dioxide exposure	1.40E-09	–	29410382	Maternal overweight/obesity	3.90E-09	–	29016858
THY1	Nitrogen dioxide exposure	1.40E-09	–	29410382	Maternal overweight/obesity	4.90E-09	–	29016858
LYNX1	Nitrogen dioxide exposure	1.40E-09	–	29410382	Maternal overweight/obesity	6.30E-09	–	29016858
TRANK1	Nitrogen dioxide exposure	1.40E-09	–	29410382	Maternal overweight/obesity, 1-hour glucose	7.20E-09	–	29016858, 33151971
LOC400931; MIRLET7B	Nitrogen dioxide exposure	1.40E-09	–	29410382	Maternal overweight/obesity	7.30E-09	–	29016858
LOC282997; PDCD4	Nitrogen dioxide exposure	1.40E-09	–	29410382	Maternal overweight/obesity	8.30E-09	–	29016858
CALHM3	Nitrogen dioxide exposure	1.40E-09	–	29410382	Maternal overweight/obesity	1.60E-08	–	29016858
FAM167A	Air pollution exposure, Nitrogen dioxide exposure	1.40E-09	–	26731791, 29410382	Maternal overweight/obesity	3.70E-08	–	29016858
NGF	Nitrogen dioxide exposure	1.50E-09	–	29410382	Maternal overweight/obesity	7.00E-10	–	29016858
FLJ23834	Nitrogen dioxide exposure	1.50E-09	–	29410382	Maternal overweight/obesity	2.50E-09	–	29016858
MEGF6	Nitrogen dioxide exposure	1.50E-09	–	29410382	Maternal overweight/obesity	2.80E-09	–	29016858
ACTR5	Nitrogen dioxide exposure	1.50E-09	–	29410382	Maternal overweight/obesity	3.70E-09	–	29016858

Gene	Trait1	minP	Direction	PMID	Trait2	minP	Direction	PMID
PRKAG2	Air pollution exposure, Nitrogen dioxide exposure	1.50E-09	–	26731791, 29410382	Maternal overweight/obesity, Diastolic blood pressure	9.60E-09	–	29016858
SYT8	Nitrogen dioxide exposure	1.50E-09	–	29410382	Maternal overweight/obesity	1.60E-08	–	29016858
KCNMA1	Air pollution exposure, Nitrogen dioxide exposure	1.50E-09	–	26731791, 29410382	Maternal overweight/obesity, fasting glucose	1.90E-08	–	29016858, 31197173
TSPO2	Nitrogen dioxide exposure	1.50E-09	–	29410382	Maternal overweight/obesity	2.00E-08	–	29016858
ZNF783	Nitrogen dioxide exposure	1.50E-09	–	29410382	Maternal overweight/obesity	3.60E-08	–	29016858
STC2	Nitrogen dioxide exposure	1.50E-09	–	29410382	Maternal overweight/obesity	4.90E-08	+	29016858
ITIH4	Nitrogen dioxide exposure	1.60E-09	–	29410382	Maternal overweight/obesity	1.70E-13	–	29016858
XYLT1	Air pollution exposure, Nitrogen dioxide exposure	1.60E-09	–	26731791, 29410382	Maternal overweight/obesity, 1-hour glucose, Glucose, HDL cholesterol, Triglycerides in very large HDL, Triglycerides to total lipids ratio in very large VLDL	3.90E-12	–	29016858, 33151971
C5orf20	Nitrogen dioxide exposure	1.60E-09	–	29410382	Maternal overweight/obesity	2.40E-11	–	29016858
TIRAP	Nitrogen dioxide exposure	1.60E-09	–	29410382	Maternal overweight/obesity	1.70E-10	–	29016858
TRAPPC9	Air pollution exposure, Nitrogen dioxide exposure	1.60E-09	–	26731791, 29410382	Maternal overweight/obesity, 1-hour glucose	5.60E-10	–	29016858, 33151971

Gene	Trait1	minP	Direction	PMID	Trait2	minP	Direction	PMID
THEM5	Nitrogen dioxide exposure	1.60E-09	–	29410382	Maternal overweight/obesity	1.10E-09	–	29016858
TNS1	Nitrogen dioxide exposure	1.60E-09	–	29410382	Maternal overweight/obesity	2.80E-09	–	29016858
TMEM169	Nitrogen dioxide exposure	1.60E-09	–	29410382	Maternal overweight/obesity	8.00E-09	–	29016858
TSTA3	Nitrogen dioxide exposure	1.60E-09	–	29410382	Maternal overweight/obesity	1.10E-08	–	29016858
KIF17	Nitrogen dioxide exposure	1.60E-09	–	29410382	Maternal overweight/obesity	1.10E-08	–	29016858
CCDC114	Nitrogen dioxide exposure	1.60E-09	–	29410382	Maternal overweight/obesity	3.20E-08	–	29016858
ZBTB46	Nitrogen dioxide exposure	1.60E-09	–	29410382	Maternal overweight/obesity, fasting glucose	3.50E-08	–	29016858, 31197173
ELFN1	Nitrogen dioxide exposure	1.60E-09	–	29410382	Maternal overweight/obesity	4.20E-08	–	29016858
GBAP1	Nitrogen dioxide exposure	1.70E-09	+	29410382	Maternal overweight/obesity	1.20E-10	+	29016858
NKD1	Nitrogen dioxide exposure	1.70E-09	–	29410382	Maternal overweight/obesity	7.60E-10	–	29016858
TPO	Nitrogen dioxide exposure	1.70E-09	–	29410382	Maternal overweight/obesity	4.00E-09	–	29016858
MYO1A	Nitrogen dioxide exposure	1.70E-09	–	29410382	Maternal overweight/obesity	6.90E-09	+	29016858
PDCD1	Nitrogen dioxide exposure	1.70E-09	–	29410382	Maternal overweight/obesity	7.90E-09	–	29016858

Gene	Trait1	minP	Direction	PMID	Trait2	minP	Direction	PMID
RYR2	Nitrogen dioxide exposure	1.70E-09	–	29410382	Maternal overweight/obesity, Triglycerides in medium HDL, Triglycerides in very small VLDL, Triglycerides to total lipids ratio in medium HDL	1.10E-08	–	29016858
HIPK2	Nitrogen dioxide exposure	1.70E-09	–	29410382	Maternal overweight/obesity	1.70E-08	+	29016858
DSCAML1	Air pollution exposure, Nitrogen dioxide exposure	1.70E-09	–	26731791, 29410382	Maternal overweight/obesity	2.40E-08	–	29016858
PLXNA4	Air pollution exposure, Nitrogen dioxide exposure, Particulate matter ≤10 um (PM10)	1.70E-09	–	26731791, 29410382, 31148503	Maternal overweight/obesity	3.20E-08	–	29016858
PANX3	Nitrogen dioxide exposure	1.80E-09	–	29410382	Maternal overweight/obesity	6.70E-10	–	29016858
AFF3	Nitrogen dioxide exposure	1.80E-09	–	29410382	Maternal overweight/obesity	7.70E-10	–	29016858
SCNN1A	Nitrogen dioxide exposure	1.80E-09	–	29410382	Maternal overweight/obesity, Diastolic Blood Pressure, Systolic Blood Pressure	9.40E-10	–	29016858, 29198723
CD276	Nitrogen dioxide exposure	1.80E-09	–	29410382	Maternal overweight/obesity	9.80E-10	–	29016858
CARS2	Air pollution exposure, Nitrogen dioxide exposure	1.80E-09	–	26731791, 29410382	Maternal overweight/obesity	1.40E-09	–	29016858

Gene	Trait1	minP	Direction	PMID	Trait2	minP	Direction	PMID
MAP2K5	Nitrogen dioxide exposure	1.80E-09	–	29410382	Maternal overweight/obesity, 1-hour glucose	2.30E-09	–	29016858, 33151971
SDK1	Air pollution exposure, Nitrogen dioxide exposure	1.80E-09	–	26731791, 29410382	Maternal overweight/obesity, Glucose, Triglycerides to total lipids ratio in IDL, Triglycerides to total lipids ratio in large LDL, Triglycerides to total lipids ratio in medium LDL, Triglycerides to total lipids ratio in medium VLDL, Triglycerides to total lipids ratio in small HDL	3.80E-09	–	29016858
PCNXL2	Nitrogen dioxide exposure	1.80E-09	–	29410382	Maternal overweight/obesity, Triglycerides	5.30E-09	–	29016858, 31910897
TDRD10	Nitrogen dioxide exposure	1.80E-09	–	29410382	Maternal overweight/obesity	7.60E-09	–	29016858
RERE	Nitrogen dioxide exposure	1.80E-09	–	29410382	Serum triglycerides, Maternal overweight/obesity, fasting glucose, Triglycerides	1.20E-08	–	28173150, 29016858, 31197173, 31910897
DHX16	Nitrogen dioxide exposure	1.80E-09	–	29410382	Maternal overweight/obesity	1.60E-08	–	29016858

Gene	Trait1	minP	Direction	PMID	Trait2	minP	Direction	PMID
ASB10	Nitrogen dioxide exposure	1.80E-09	–	29410382	Maternal overweight/obesity	2.40E-08	–	29016858
GGT1	Nitrogen dioxide exposure	1.80E-09	–	29410382	Maternal overweight/obesity	2.80E-08	–	29016858
MAP3K7IP1	Nitrogen dioxide exposure	1.80E-09	+	29410382	Maternal overweight/obesity, fasting glucose	2.90E-08	+	29016858, 31197173
AK3L1	Nitrogen dioxide exposure	1.80E-09	–	29410382	Maternal overweight/obesity	3.70E-08	–	29016858
JAKMIP3	Nitrogen dioxide exposure	1.80E-09	–	29410382	Maternal overweight/obesity	4.20E-08	–	29016858
ASB18	Nitrogen dioxide exposure	1.90E-09	–	29410382	Maternal overweight/obesity	1.40E-12	–	29016858
KIAA1688	Air pollution exposure, Nitrogen dioxide exposure	1.90E-09	–	26731791, 29410382	Maternal overweight/obesity	1.20E-11	–	29016858
ARHGAP18	Nitrogen dioxide exposure	1.90E-09	–	29410382	Maternal overweight/obesity, 1-hour glucose	2.80E-11	–	29016858, 33151971
PACRG;LOC 285796	Nitrogen dioxide exposure	1.90E-09	–	29410382	Maternal overweight/obesity	2.70E-10	–	29016858
ALDH1L1	Nitrogen dioxide exposure	1.90E-09	–	29410382	Maternal overweight/obesity, Triglycerides in chylomicrons and extremely large VLDL, Triglycerides in small VLDL, Triglycerides in very large VLDL	1.00E-09	–	29016858

Gene	Trait1	minP	Direction	PMID	Trait2	minP	Direction	PMID
FAM19A5	Air pollution exposure, Nitrogen dioxide exposure	1.90E-09	–	26731791, 29410382	Maternal overweight/obesity	5.20E-09	–	29016858
SLC2A14	Nitrogen dioxide exposure	1.90E-09	–	29410382	Maternal overweight/obesity	1.00E-08	–	29016858
DNAH10	Nitrogen dioxide exposure	1.90E-09	–	29410382	Maternal overweight/obesity, fasting glucose	4.70E-08	–	29016858, 31197173
SGSH	Nitrogen dioxide exposure	2.00E-09	–	29410382	Maternal overweight/obesity	2.30E-11	–	29016858
MIR451;MIR 144	Nitrogen dioxide exposure	2.00E-09	–	29410382	Maternal overweight/obesity	6.10E-10	–	29016858
KCNH4	Nitrogen dioxide exposure	2.00E-09	–	29410382	Maternal overweight/obesity	6.80E-10	–	29016858
PHYHIP	Nitrogen dioxide exposure	2.00E-09	–	29410382	Maternal overweight/obesity	8.50E-10	–	29016858
MUC5B	Nitrogen dioxide exposure	2.00E-09	–	29410382	Maternal overweight/obesity	1.00E-09	–	29016858
ADRA1B	Nitrogen dioxide exposure	2.00E-09	–	29410382	Maternal overweight/obesity	1.60E-09	–	29016858
PLXNB1	Nitrogen dioxide exposure	2.00E-09	–	29410382	Maternal overweight/obesity	3.90E-09	–	29016858
KCNQ1	Nitrogen dioxide exposure	2.00E-09	–	29410382	Maternal overweight/obesity	5.10E-09	–	29016858
FGF1	Nitrogen dioxide exposure	2.00E-09	–	29410382	Maternal overweight/obesity	1.90E-08	–	29016858
ITPR3	Air pollution exposure, Nitrogen dioxide exposure	2.00E-09	–	26731791, 29410382	Maternal overweight/obesity	3.20E-08	–	29016858
PIK3R5	Air pollution exposure, Nitrogen dioxide exposure	2.10E-09	–	26731791, 29410382	Maternal overweight/obesity	1.70E-12	–	29016858

Gene	Trait1	minP	Direction	PMID	Trait2	minP	Direction	PMID
ATP6V1B1	Nitrogen dioxide exposure	2.10E-09	–	29410382	Maternal overweight/obesity	7.40E-11	–	29016858
CCR2	Nitrogen dioxide exposure	2.10E-09	–	29410382	Maternal overweight/obesity	9.80E-11	–	29016858
ADARB2	Air pollution exposure, Nitrogen dioxide exposure	2.10E-09	–	26731791, 29410382	Maternal overweight/obesity	4.30E-10	–	29016858
PDE4DIP	Nitrogen dioxide exposure	2.10E-09	–	29410382	Maternal overweight/obesity, fasting glucose	5.20E-10	–	29016858, 31197173
BSN	Nitrogen dioxide exposure	2.10E-09	–	29410382	Maternal overweight/obesity, 1-hour glucose	1.40E-09	–	29016858, 33151971
CELSR1	Nitrogen dioxide exposure	2.10E-09	–	29410382	Maternal overweight/obesity	2.50E-09	–	29016858
JAKMIP1	Nitrogen dioxide exposure	2.10E-09	–	29410382	Maternal overweight/obesity	6.60E-09	–	29016858
LOC619207	Air pollution exposure, Nitrogen dioxide exposure	2.10E-09	–	26731791, 29410382	Maternal overweight/obesity	1.30E-08	–	29016858
NLRC3	Nitrogen dioxide exposure	2.10E-09	–	29410382	Maternal overweight/obesity	1.40E-08	–	29016858
C6orf186	Nitrogen dioxide exposure	2.20E-09	–	29410382	Maternal overweight/obesity	3.00E-11	–	29016858
ACTA2	Nitrogen dioxide exposure	2.20E-09	–	29410382	Maternal overweight/obesity	9.50E-10	–	29016858
DUSP27	Nitrogen dioxide exposure	2.20E-09	–	29410382	Maternal overweight/obesity	1.20E-08	–	29016858
BCCIP;UROS	Nitrogen dioxide exposure	2.20E-09	–	29410382	Maternal overweight/obesity	1.20E-08	–	29016858
TAS2R60	Nitrogen dioxide exposure	2.20E-09	–	29410382	Maternal overweight/obesity	3.30E-08	–	29016858

Gene	Trait1	minP	Direction	PMID	Trait2	minP	Direction	PMID
PPT2	Nitrogen dioxide exposure	2.30E-09	–	29410382	Maternal overweight/obesity	2.60E-12	–	29016858
MYH14	Nitrogen dioxide exposure	2.30E-09	–	29410382	Maternal overweight/obesity	3.00E-10	–	29016858
WWC1	Nitrogen dioxide exposure, Particulate matter <2.5um (PM2.5)	2.30E-09	–	29410382, 31208937	Maternal overweight/obesity	1.50E-09	–	29016858
PADI1	Nitrogen dioxide exposure	2.30E-09	–	29410382	Maternal overweight/obesity	1.70E-09	–	29016858
PITPNM2	Nitrogen dioxide exposure	2.30E-09	–	29410382	Maternal overweight/obesity, fasting glucose, 1-hour glucose	2.00E-09	–	29016858, 31197173, 33151971
ARHGEF10	Nitrogen dioxide exposure	2.30E-09	–	29410382	Maternal overweight/obesity, Triglycerides to total lipids ratio in medium LDL, Triglycerides to total lipids ratio in small LDL	7.70E-09	–	29016858
AATK	Air pollution exposure, Nitrogen dioxide exposure	2.30E-09	–	26731791, 29410382	Maternal overweight/obesity, Triglycerides to total lipids ratio in chylomicrons and XXL-VLDL	1.10E-08	–	29016858
RASGRF1	Nitrogen dioxide exposure, Particulate matter <2.5um (PM2.5)	2.30E-09	–	29410382	Maternal overweight/obesity	1.30E-08	–	29016858

Gene	Trait1	minP	Direction	PMID	Trait2	minP	Direction	PMID
ARNTL	Nitrogen dioxide exposure	2.30E-09	–	29410382	Maternal overweight/obesity	1.70E-08	–	29016858
ATP2B4	Nitrogen dioxide exposure	2.30E-09	–	29410382	Maternal overweight/obesity	1.80E-08	–	29016858
ZCCHC24	Nitrogen dioxide exposure	2.30E-09	–	29410382	Maternal overweight/obesity	3.70E-08	–	29016858
GPR45	Nitrogen dioxide exposure	2.30E-09	–	29410382	Maternal overweight/obesity	3.90E-08	–	29016858
SYNJ2	Nitrogen dioxide exposure	2.30E-09	–	29410382	Maternal overweight/obesity, fasting glucose	4.50E-08	–	29016858, 31197173
LOC154822	Nitrogen dioxide exposure	2.30E-09	–	29410382	Maternal overweight/obesity	4.80E-08	–	29016858
ARNT2	Nitrogen dioxide exposure	2.40E-09	–	29410382	Maternal overweight/obesity, Triglycerides in chylomicrons and extremely large VLDL	3.80E-10	–	29016858
MORN1	Nitrogen dioxide exposure	2.40E-09	–	29410382	Maternal overweight/obesity, Diastolic blood pressure, Triglycerides to total lipids ratio in medium HDL	2.70E-09	–	29016858, 32916427
C15orf50	Nitrogen dioxide exposure	2.40E-09	–	29410382	Maternal overweight/obesity	5.40E-09	–	29016858
NFATC1	Air pollution exposure, Nitrogen dioxide exposure, Particulate matter air pollution	2.40E-09	–	26731791, 29410382, 32484729	Maternal overweight/obesity	7.20E-09	–	29016858

Gene	Trait1	minP	Direction	PMID	Trait2	minP	Direction	PMID
AGAP1	Air pollution exposure, Nitrogen dioxide exposure	2.40E-09	–	26731791, 29410382	Maternal overweight/obesity	8.90E-09	–	29016858
NAGPA	Nitrogen dioxide exposure	2.40E-09	–	29410382	Maternal overweight/obesity	2.10E-08	–	29016858
NAV2	Nitrogen dioxide exposure	2.40E-09	–	29410382	Maternal overweight/obesity	3.80E-08	–	29016858
APOA5	Nitrogen dioxide exposure	2.50E-09	–	29410382	Triglycerides, Maternal overweight/obesity	9.40E-13	–	25583993, 29016858
PDE9A	Nitrogen dioxide exposure	2.50E-09	–	29410382	Maternal overweight/obesity, 1-hour glucose	7.10E-12	–	29016858, 33151971
CELSR3	Air pollution exposure, Nitrogen dioxide exposure	2.50E-09	–	26731791, 29410382	Maternal overweight/obesity	1.30E-10	–	29016858
SH3PXD2B	Nitrogen dioxide exposure	2.50E-09	–	29410382	Maternal overweight/obesity	1.30E-09	–	29016858
LMCD1	Nitrogen dioxide exposure	2.50E-09	–	29410382	Maternal overweight/obesity	4.60E-09	–	29016858
CADPS2	Nitrogen dioxide exposure	2.50E-09	–	29410382	Maternal overweight/obesity	1.00E-08	–	29016858
F7	Nitrogen dioxide exposure	2.50E-09	–	29410382	Maternal overweight/obesity	1.20E-08	–	29016858
PRR5;PRR5-ARHGAP8	Nitrogen dioxide exposure	2.50E-09	–	29410382	Maternal overweight/obesity	2.80E-08	–	29016858
GMIP	Nitrogen dioxide exposure	2.50E-09	–	29410382	Maternal overweight/obesity	2.90E-08	–	29016858

Gene	Trait1	minP	Direction	PMID	Trait2	minP	Direction	PMID
PHGDH	Nitrogen dioxide exposure	2.60E-09	–	29410382	Serum triglycerides, Maternal overweight/obesity, Diastolic Blood Pressure, Systolic Blood Pressure, fasting glucose, Serum total triglycerides, Triglycerides, Triglycerides in HDL, Triglycerides in large VLDL, Triglycerides in medium HDL, Triglycerides in medium VLDL, Triglycerides in small HDL, Triglycerides in small VLDL, Triglycerides in VLDL, Triglycerides to total lipids ratio in medium HDL, Triglycerides to total lipids ratio in small VLDL	2.70E-34	–	28213390, 29016858, 29198723, 31197173, 32520614
HBBP1	Nitrogen dioxide exposure	2.60E-09	–	29410382	Maternal overweight/obesity	7.40E-12	–	29016858
NPR1	Nitrogen dioxide exposure	2.60E-09	–	29410382	Maternal overweight/obesity	3.00E-11	–	29016858
ALKBH1;C14orf156	Nitrogen dioxide exposure	2.60E-09	–	29410382	Maternal overweight/obesity	2.20E-10	–	29016858

Gene	Trait1	minP	Direction	PMID	Trait2	minP	Direction	PMID
SNED1	Air pollution exposure, Nitrogen dioxide exposure	2.60E-09	–	26731791, 29410382	Maternal overweight/obesity	1.20E-09	–	29016858
DNAH17	Air pollution exposure, Nitrogen dioxide exposure	2.60E-09	–	26731791, 29410382	Maternal overweight/obesity	2.40E-09	–	29016858
FAM107A	Air pollution exposure, Nitrogen dioxide exposure	2.60E-09	–	26731791, 29410382	Maternal overweight/obesity	4.90E-09	–	29016858
PRPH2	Nitrogen dioxide exposure	2.60E-09	–	29410382	Maternal overweight/obesity	8.60E-09	–	29016858
ADAMTSL1	Nitrogen dioxide exposure	2.60E-09	–	29410382	Maternal overweight/obesity	2.20E-08	–	29016858
SLC39A11	Nitrogen dioxide exposure	2.60E-09	–	29410382	Maternal overweight/obesity	3.40E-08	–	29016858
DNAH2	Nitrogen dioxide exposure	2.60E-09	–	29410382	Maternal overweight/obesity, 1-hour glucose, Triglycerides to total lipids ratio in small VLDL	4.00E-08	–	29016858, 33151971
GUCA2A	Nitrogen dioxide exposure	2.60E-09	–	29410382	Maternal overweight/obesity	4.50E-08	–	29016858
LRRC8D	Nitrogen dioxide exposure	2.70E-09	–	29410382	Maternal overweight/obesity, Diastolic Blood Pressure, Systolic Blood Pressure	3.30E-10	–	29016858, 29198723
SH3RF3	Nitrogen dioxide exposure	2.70E-09	–	29410382	Maternal overweight/obesity	3.50E-10	–	29016858
GPR89A	Nitrogen dioxide exposure	2.70E-09	–	29410382	Maternal overweight/obesity	1.10E-08	–	29016858

Gene	Trait1	minP	Direction	PMID	Trait2	minP	Direction	PMID
TFIP11	Nitrogen dioxide exposure	2.70E-09	–	29410382	Maternal overweight/obesity	1.20E-08	–	29016858
CDH23	Nitrogen dioxide exposure	2.70E-09	–	29410382	Maternal overweight/obesity, Systolic blood pressure	1.70E-08	–	29016858
ALK	Nitrogen dioxide exposure	2.70E-09	–	29410382	Maternal overweight/obesity	1.90E-08	–	29016858
TTBK1	Air pollution exposure, Nitrogen dioxide exposure	2.70E-09	–	26731791, 29410382	Maternal overweight/obesity	2.80E-08	–	29016858
RAI14	Nitrogen dioxide exposure	2.70E-09	–	29410382	Maternal overweight/obesity	3.70E-08	–	29016858
ST7	Nitrogen dioxide exposure	2.70E-09	–	29410382	Maternal overweight/obesity	4.90E-08	–	29016858
BDH1	Nitrogen dioxide exposure	2.80E-09	–	29410382	Maternal overweight/obesity	1.60E-12	–	29016858
CACNA1H	Nitrogen dioxide exposure	2.80E-09	–	29410382	Maternal overweight/obesity, Triglycerides to total lipids ratio in small HDL, Triglycerides to total lipids ratio in small LDL, Triglycerides to total lipids ratio in very small VLDL	7.60E-11	–	29016858, 32916427
CA12	Nitrogen dioxide exposure	2.80E-09	–	29410382	Maternal overweight/obesity	1.30E-10	–	29016858
SOCS7	Nitrogen dioxide exposure	2.80E-09	–	29410382	Maternal overweight/obesity, Triglycerides to	2.50E-10	–	29016858

Gene	Trait1	minP	Direction	PMID	Trait2	minP	Direction	PMID
					total lipids ratio in very large VLDL			
MTUS2	Nitrogen dioxide exposure	2.80E-09	–	29410382	Maternal overweight/obesity	1.10E-09	–	29016858, 32916427
CSMD1	Nitrogen dioxide exposure	2.80E-09	–	29410382	Maternal overweight/obesity	3.20E-09	–	29016858
MSH5	Nitrogen dioxide exposure	2.80E-09	–	29410382	Maternal overweight/obesity	7.40E-09	–	29016858
CAMTA1	Nitrogen dioxide exposure	2.80E-09	–	29410382	Maternal overweight/obesity, 1-hour glucose	9.70E-09	–	29016858, 32916427, 33151971
ETV3L	Nitrogen dioxide exposure	2.80E-09	–	29410382	Maternal overweight/obesity	2.90E-08	–	29016858
LOC100133669	Air pollution exposure, Nitrogen dioxide exposure	2.80E-09	–	26731791, 29410382	Maternal overweight/obesity	3.10E-08	–	29016858
PRCD	Nitrogen dioxide exposure	2.90E-09	–	29410382	Maternal overweight/obesity	6.00E-11	–	29016858
NXN	Air pollution exposure, Nitrogen dioxide exposure, Particulate matter air pollution	2.90E-09	–	26731791, 29410382, 32484729	Maternal overweight/obesity, 1-hour glucose, Systolic blood pressure	1.10E-08	–	29016858, 33151971
EXT2	Nitrogen dioxide exposure	2.90E-09	–	29410382	Maternal overweight/obesity	1.90E-08	–	29016858
RASGEF1C	Nitrogen dioxide exposure	2.90E-09	–	29410382	Maternal overweight/obesity	2.60E-08	–	29016858

Gene	Trait1	minP	Direction	PMID	Trait2	minP	Direction	PMID
HDDC3;UNC45A	Nitrogen dioxide exposure	2.90E-09	–	29410382	Maternal overweight/obesity	3.10E-08	–	29016858
TAP2	Nitrogen dioxide exposure	3.00E-09	–	29410382	Maternal overweight/obesity	2.30E-11	–	29016858
ANKDD1A	Nitrogen dioxide exposure	3.00E-09	–	29410382	Maternal overweight/obesity	1.80E-10	–	29016858
TNK2	Nitrogen dioxide exposure	3.00E-09	–	29410382	Maternal overweight/obesity	4.30E-10	–	29016858
EML1	Nitrogen dioxide exposure	3.00E-09	–	29410382	Maternal overweight/obesity	1.50E-09	–	29016858
ODZ4	Nitrogen dioxide exposure	3.00E-09	–	29410382	Maternal overweight/obesity	5.40E-09	–	29016858
CD48	Nitrogen dioxide exposure	3.00E-09	+	29410382	Maternal overweight/obesity	1.20E-08	+	29016858
CLEC4A	Nitrogen dioxide exposure	3.00E-09	–	29410382	Maternal overweight/obesity	1.30E-08	–	29016858
SCOC	Nitrogen dioxide exposure	3.00E-09	–	29410382	Maternal overweight/obesity	1.90E-08	–	29016858
CABIN1	Nitrogen dioxide exposure	3.00E-09	–	29410382	Maternal overweight/obesity	2.50E-08	–	29016858
BCL2	Nitrogen dioxide exposure	3.00E-09	+	29410382	Maternal overweight/obesity	2.80E-08	+	29016858
PZP	Nitrogen dioxide exposure	3.10E-09	–	29410382	Maternal overweight/obesity	6.60E-11	–	29016858
RGS6	Nitrogen dioxide exposure	3.10E-09	–	29410382	Maternal overweight/obesity, 1-hour glucose	6.70E-10	–	29016858, 33151971
C4orf29	Nitrogen dioxide exposure	3.10E-09	–	29410382	Maternal overweight/obesity	7.10E-10	–	29016858
CHTF18	Nitrogen dioxide exposure	3.10E-09	–	29410382	Maternal overweight/obesity	8.80E-10	–	29016858

Gene	Trait1	minP	Direction	PMID	Trait2	minP	Direction	PMID
CLSTN1	Nitrogen dioxide exposure	3.10E-09	–	29410382	Maternal overweight/obesity	1.00E-09	–	29016858
SSTR3	Nitrogen dioxide exposure	3.10E-09	–	29410382	Maternal overweight/obesity	4.10E-09	–	29016858
CISH	Nitrogen dioxide exposure	3.10E-09	–	29410382	Maternal overweight/obesity, Triglycerides in large VLDL, Triglycerides in medium HDL, Triglycerides to total lipids ratio in small VLDL	6.60E-09	–	29016858
CLIP2	Nitrogen dioxide exposure	3.10E-09	–	29410382	Maternal overweight/obesity	9.20E-09	–	29016858
NUPR1	Nitrogen dioxide exposure	3.10E-09	–	29410382	Maternal overweight/obesity	1.30E-08	–	29016858
SYNGAP1	Nitrogen dioxide exposure	3.10E-09	–	29410382	Maternal overweight/obesity	1.90E-08	–	29016858
FLNB	Nitrogen dioxide exposure	3.10E-09	–	29410382	Maternal overweight/obesity, 1-hour glucose	2.20E-08	–	29016858, 33151971
ZNF837	Nitrogen dioxide exposure	3.20E-09	–	29410382	Maternal overweight/obesity	1.90E-10	–	29016858
C7orf54;SND1	Nitrogen dioxide exposure	3.20E-09	–	29410382	Maternal overweight/obesity	3.10E-09	–	29016858
ARHGAP10	Nitrogen dioxide exposure	3.20E-09	–	29410382	Maternal overweight/obesity	4.40E-09	–	29016858
PRKCA	Nitrogen dioxide exposure	3.20E-09	–	29410382	Maternal overweight/obesity	4.50E-09	–	29016858
CPNE9	Nitrogen dioxide exposure	3.20E-09	–	29410382	Maternal overweight/obesity	2.20E-08	–	29016858

Gene	Trait1	minP	Direction	PMID	Trait2	minP	Direction	PMID
RLN3	Nitrogen dioxide exposure	3.20E-09	–	29410382	Maternal overweight/obesity	4.00E-08	–	29016858
WSCD1	Nitrogen dioxide exposure	3.30E-09	–	29410382	Maternal overweight/obesity	1.50E-11	–	29016858
PACS2	Nitrogen dioxide exposure	3.30E-09	–	29410382	Maternal overweight/obesity, Ratio of triglycerides to phosphoglycerides, Systolic blood pressure	2.20E-10	–	29016858
TRIM10	Nitrogen dioxide exposure	3.30E-09	–	29410382	Maternal overweight/obesity	8.20E-10	–	29016858
TBC1D16	Air pollution exposure, Nitrogen dioxide exposure	3.30E-09	–	26731791, 29410382	Maternal overweight/obesity, Diastolic blood pressure	3.70E-09	–	29016858
CDRT4	Nitrogen dioxide exposure	3.30E-09	–	29410382	Maternal overweight/obesity	9.30E-09	–	29016858
LPPR2	Nitrogen dioxide exposure	3.30E-09	–	29410382	Maternal overweight/obesity, 1-hour glucose	1.00E-08	–	29016858, 33151971
TCF23	Nitrogen dioxide exposure	3.30E-09	–	29410382	Maternal overweight/obesity	1.80E-08	–	29016858
C14orf68	Nitrogen dioxide exposure	3.30E-09	–	29410382	Maternal overweight/obesity	2.90E-08	–	29016858
A2LD1	Nitrogen dioxide exposure	3.30E-09	–	29410382	Maternal overweight/obesity	4.50E-08	–	29016858
QSOX1	Nitrogen dioxide exposure	3.40E-09	–	29410382	Maternal overweight/obesity	1.40E-10	–	29016858
UBXN6	Nitrogen dioxide exposure	3.40E-09	–	29410382	Maternal overweight/obesity	6.30E-10	–	29016858

Gene	Trait1	minP	Direction	PMID	Trait2	minP	Direction	PMID
ROR2	Nitrogen dioxide exposure	3.40E-09	–	29410382	Maternal overweight/obesity	1.40E-09	–	29016858
JUB	Nitrogen dioxide exposure	3.40E-09	–	29410382	Maternal overweight/obesity	4.30E-09	–	29016858, 32916427
CHIT1	Nitrogen dioxide exposure	3.40E-09	–	29410382	Maternal overweight/obesity	3.00E-08	–	29016858
TP53I11	Nitrogen dioxide exposure	3.40E-09	–	29410382	Maternal overweight/obesity, Triglycerides	4.50E-08	–	29016858, 31910897
DGCR9	Nitrogen dioxide exposure	3.50E-09	–	29410382	Maternal overweight/obesity	1.10E-11	–	29016858
LMF1	Air pollution exposure, Nitrogen dioxide exposure	3.50E-09	–	26731791, 29410382	Maternal overweight/obesity, Triglycerides in small LDL	5.40E-10	–	29016858
HDAC11	Nitrogen dioxide exposure	3.50E-09	–	29410382	Maternal overweight/obesity	1.50E-09	–	29016858
CIITA	Nitrogen dioxide exposure	3.50E-09	–	29410382	Maternal overweight/obesity	4.10E-09	–	29016858
GPR39	Nitrogen dioxide exposure	3.50E-09	–	29410382	Maternal overweight/obesity	8.60E-09	–	29016858
RHOBTB2	Nitrogen dioxide exposure	3.50E-09	–	29410382	Maternal overweight/obesity	1.00E-08	–	29016858
AKNAD1	Nitrogen dioxide exposure	3.50E-09	–	29410382	Maternal overweight/obesity	4.40E-08	–	29016858
C6orf168	Air pollution exposure, Nitrogen dioxide exposure	3.60E-09	–	26731791, 29410382	Maternal overweight/obesity	5.80E-11	–	29016858
IGFALS	Nitrogen dioxide exposure	3.60E-09	–	29410382	Maternal overweight/obesity	3.90E-09	–	29016858

Gene	Trait1	minP	Direction	PMID	Trait2	minP	Direction	PMID
ZNF20	Nitrogen dioxide exposure	3.60E-09	–	29410382	Maternal overweight/obesity	4.60E-09	–	29016858
VPS18	Nitrogen dioxide exposure	3.60E-09	–	29410382	Maternal overweight/obesity, Triglycerides to total lipids ratio in chylomicrons and XXL-VLDL	1.60E-08	–	29016858
MAPT;STH	Nitrogen dioxide exposure	3.60E-09	–	29410382	Maternal overweight/obesity	3.50E-08	–	29016858
CYP3A7	Nitrogen dioxide exposure	3.70E-09	–	29410382	Maternal overweight/obesity	4.20E-13	–	29016858
JPH3	Nitrogen dioxide exposure	3.70E-09	–	29410382	Maternal overweight/obesity	2.00E-10	–	29016858
FAM179A	Nitrogen dioxide exposure	3.70E-09	–	29410382	Maternal overweight/obesity	4.50E-10	–	29016858
C7orf50	Air pollution exposure, Nitrogen dioxide exposure, Particulate matter ≤10 um (PM10)	3.70E-09	–	26731791, 29410382, 31148503	Maternal overweight/obesity, HDL cholesterol	6.70E-09	–	29016858
ZNF792	Nitrogen dioxide exposure	3.70E-09	–	29410382	Maternal overweight/obesity	1.00E-08	–	29016858
OR2S2	Nitrogen dioxide exposure	3.70E-09	–	29410382	Maternal overweight/obesity	1.40E-08	–	29016858
BEND6	Nitrogen dioxide exposure	3.70E-09	–	29410382	Maternal overweight/obesity	2.40E-08	–	29016858
ZFHX3	Nitrogen dioxide exposure	3.80E-09	–	29410382	Maternal overweight/obesity	2.00E-11	–	29016858
SH3TC2	Nitrogen dioxide exposure	3.80E-09	–	29410382	Maternal overweight/obesity	5.80E-11	–	29016858

Gene	Trait1	minP	Direction	PMID	Trait2	minP	Direction	PMID
SLC22A18;SLC22A18AS	Nitrogen dioxide exposure	3.80E-09	–	29410382	Maternal overweight/obesity, fasting glucose	3.10E-10	–	29016858, 31197173
OR6P1	Nitrogen dioxide exposure	3.80E-09	–	29410382	Maternal overweight/obesity	1.40E-09	–	29016858
C1orf127	Nitrogen dioxide exposure	3.80E-09	–	29410382	Maternal overweight/obesity, Glucose	3.40E-08	–	29016858
CDH4	Air pollution exposure, Nitrogen dioxide exposure	3.80E-09	–	26731791, 29410382	Maternal overweight/obesity	4.70E-08	–	29016858
WNT9A	Nitrogen dioxide exposure	3.80E-09	–	29410382	Maternal overweight/obesity	4.70E-08	–	29016858
BAI2	Air pollution exposure, Nitrogen dioxide exposure	3.90E-09	–	26731791, 29410382	Maternal overweight/obesity	7.50E-11	–	29016858
RFTN1	Nitrogen dioxide exposure	3.90E-09	–	29410382	Maternal overweight/obesity	2.40E-10	–	29016858
DIRC3	Nitrogen dioxide exposure	3.90E-09	–	29410382	Maternal overweight/obesity	5.40E-10	–	29016858
CRHR1	Nitrogen dioxide exposure	3.90E-09	–	29410382	Maternal overweight/obesity	1.10E-09	–	29016858
COL4A1	Nitrogen dioxide exposure	3.90E-09	–	29410382	Maternal overweight/obesity	2.90E-09	–	29016858
CUX2	Nitrogen dioxide exposure	3.90E-09	–	29410382	Maternal overweight/obesity	3.30E-09	–	29016858
KLK13	Nitrogen dioxide exposure	3.90E-09	–	29410382	Maternal overweight/obesity	8.80E-09	–	29016858
DLG5	Nitrogen dioxide exposure	3.90E-09	–	29410382	Maternal overweight/obesity	1.30E-08	–	29016858

Gene	Trait1	minP	Direction	PMID	Trait2	minP	Direction	PMID
DLGAP2	Air pollution exposure, Nitrogen dioxide exposure	3.90E-09	–	26731791, 29410382	Maternal overweight/obesity	1.50E-08	–	29016858
HS3ST4	Nitrogen dioxide exposure	3.90E-09	–	29410382	Maternal overweight/obesity	4.40E-08	–	29016858
PSMB9	Nitrogen dioxide exposure	4.00E-09	–	29410382	Maternal overweight/obesity	5.50E-09	–	29016858
KIRREL	Nitrogen dioxide exposure	4.00E-09	–	29410382	Maternal overweight/obesity	6.10E-09	–	29016858
ZNF831	Nitrogen dioxide exposure	4.00E-09	–	29410382	Maternal overweight/obesity	6.50E-09	–	29016858
RASL10B	Nitrogen dioxide exposure	4.00E-09	–	29410382	Maternal overweight/obesity	2.70E-08	–	29016858
FAM107B	Nitrogen dioxide exposure	4.00E-09	–	29410382	Maternal overweight/obesity	3.20E-08	–	29016858
TTC7A	Nitrogen dioxide exposure	4.10E-09	–	29410382	Maternal overweight/obesity	2.20E-10	–	29016858
RHBDF1	Nitrogen dioxide exposure	4.10E-09	–	29410382	Maternal overweight/obesity	4.70E-10	–	29016858
FOXJ1	Nitrogen dioxide exposure	4.10E-09	–	29410382	Maternal overweight/obesity	7.40E-09	–	29016858
TCF7L1	Nitrogen dioxide exposure	4.10E-09	–	29410382	Maternal overweight/obesity, Triglycerides in medium VLDL, Triglycerides in small VLDL, Triglycerides in very small VLDL	4.00E-08	–	29016858
VAV2	Nitrogen dioxide exposure	4.20E-09	–	29410382	Maternal overweight/obesity, 1-hour glucose	8.10E-13	–	29016858, 33151971

Gene	Trait1	minP	Direction	PMID	Trait2	minP	Direction	PMID
TP73	Nitrogen dioxide exposure	4.20E-09	–	29410382	Maternal overweight/obesity	4.70E-09	–	29016858
EMP1	Nitrogen dioxide exposure	4.20E-09	–	29410382	Maternal overweight/obesity	7.50E-09	–	29016858
TRPM3	Nitrogen dioxide exposure	4.30E-09	–	29410382	Maternal overweight/obesity	1.40E-09	–	29016858
MMP15	Nitrogen dioxide exposure	4.30E-09	–	29410382	Maternal overweight/obesity	2.70E-09	–	29016858
TMEM119	Nitrogen dioxide exposure	4.30E-09	–	29410382	Maternal overweight/obesity	1.10E-08	–	29016858
CELA1	Nitrogen dioxide exposure	4.30E-09	–	29410382	Maternal overweight/obesity	1.60E-08	–	29016858
RADIL	Nitrogen dioxide exposure	4.30E-09	–	29410382	Maternal overweight/obesity	2.50E-08	–	29016858
UCN	Nitrogen dioxide exposure	4.30E-09	–	29410382	Maternal overweight/obesity	3.20E-08	–	29016858
MAP3K9	Nitrogen dioxide exposure	4.40E-09	–	29410382	Maternal overweight/obesity	3.50E-10	–	29016858
LOXL2	Nitrogen dioxide exposure	4.40E-09	–	29410382	Maternal overweight/obesity	4.40E-10	–	29016858
RPUSD1	Nitrogen dioxide exposure	4.40E-09	–	29410382	Maternal overweight/obesity	2.20E-09	–	29016858
IL1F7	Nitrogen dioxide exposure	4.40E-09	–	29410382	Maternal overweight/obesity, Triglycerides to total lipids ratio in large VLDL	2.50E-09	–	29016858
CCDC136	Nitrogen dioxide exposure	4.40E-09	–	29410382	Maternal overweight/obesity	1.30E-08	–	29016858
PLCB3	Nitrogen dioxide exposure	4.50E-09	–	29410382	Maternal overweight/obesity	4.10E-10	–	29016858
LACRT	Nitrogen dioxide exposure	4.50E-09	–	29410382	Maternal overweight/obesity	8.30E-09	–	29016858

Gene	Trait1	minP	Direction	PMID	Trait2	minP	Direction	PMID
ITLN1	Nitrogen dioxide exposure	4.50E-09	–	29410382	Maternal overweight/obesity	1.20E-08	–	29016858
KIF1A	Nitrogen dioxide exposure	4.50E-09	–	29410382	Maternal overweight/obesity	1.30E-08	–	29016858
RNF220	Air pollution exposure, Nitrogen dioxide exposure	4.50E-09	–	26731791, 29410382	Maternal overweight/obesity	1.50E-08	–	29016858
CCDC85C	Air pollution exposure, Nitrogen dioxide exposure	4.60E-09	–	26731791, 29410382	Maternal overweight/obesity, fasting glucose	1.40E-09	–	29016858, 31197173
ARHGAP26	Nitrogen dioxide exposure	4.60E-09	–	29410382	Maternal overweight/obesity, Triglycerides in small HDL, Triglycerides in small VLDL, Triglycerides in very small VLDL, Triglycerides to total lipids ratio in large LDL, Triglycerides to total lipids ratio in medium HDL, Triglycerides to total lipids ratio in small HDL	1.10E-08	–	29016858
SYT16	Nitrogen dioxide exposure	4.60E-09	–	29410382	Maternal overweight/obesity	1.80E-08	–	29016858
ZP1	Nitrogen dioxide exposure	4.60E-09	+	29410382	Maternal overweight/obesity	2.40E-08	+	29016858
PHACTR2	Nitrogen dioxide exposure	4.60E-09	–	29410382	Maternal overweight/obesity	4.50E-08	–	29016858

Gene	Trait1	minP	Direction	PMID	Trait2	minP	Direction	PMID
ATP1B1	Nitrogen dioxide exposure	4.70E-09	–	29410382	Maternal overweight/obesity	2.90E-12	–	29016858
PTPN21	Nitrogen dioxide exposure	4.70E-09	–	29410382	Maternal overweight/obesity	2.80E-10	–	29016858
RHBG	Nitrogen dioxide exposure	4.70E-09	–	29410382	Maternal overweight/obesity	9.00E-10	–	29016858
PHLDB1	Air pollution exposure, Nitrogen dioxide exposure	4.70E-09	–	26731791, 29410382	Maternal overweight/obesity	6.60E-09	–	29016858
CYB561D1	Nitrogen dioxide exposure	4.70E-09	–	29410382	Maternal overweight/obesity	1.30E-08	–	29016858
TG;SLA	Nitrogen dioxide exposure	4.70E-09	–	29410382	Maternal overweight/obesity	1.50E-08	–	29016858
STRA8	Nitrogen dioxide exposure	4.70E-09	–	29410382	Maternal overweight/obesity	1.90E-08	–	29016858
HCRTR1	Nitrogen dioxide exposure	4.70E-09	–	29410382	Maternal overweight/obesity, Triglycerides in very small VLDL	4.60E-08	–	29016858
FCHSD2	Nitrogen dioxide exposure	4.80E-09	–	29410382	Obesity	2.50E-09	–	32788176
SEMA4G	Nitrogen dioxide exposure	4.80E-09	–	29410382	Maternal overweight/obesity	3.70E-09	–	29016858
LOC100129066	Air pollution exposure, Nitrogen dioxide exposure	4.80E-09	–	26731791, 29410382	Maternal overweight/obesity	9.40E-09	–	29016858
DAGLA	Air pollution exposure, Nitrogen dioxide exposure	4.80E-09	–	26731791, 29410382	Maternal overweight/obesity, 1-hour glucose, Triglycerides to total lipids ratio in very large VLDL	1.40E-08	–	29016858, 33151971

Gene	Trait1	minP	Direction	PMID	Trait2	minP	Direction	PMID
AHRR	Nitrogen dioxide exposure	4.90E-09	–	29410382	Maternal overweight/obesity	3.90E-10	–	29016858
DPEP1	Nitrogen dioxide exposure	4.90E-09	–	29410382	Maternal overweight/obesity, Triglycerides to total lipids ratio in IDL, Triglycerides to total lipids ratio in small LDL, Triglycerides to total lipids ratio in small VLDL, Triglycerides to total lipids ratio in very small VLDL	7.60E-10	–	29016858
KANK3	Nitrogen dioxide exposure	4.90E-09	–	29410382	Maternal overweight/obesity	6.00E-09	–	29016858
HLA-DPB1	Nitrogen dioxide exposure	4.90E-09	–	29410382	Maternal overweight/obesity	2.10E-08	–	29016858
LOC285419	Nitrogen dioxide exposure	4.90E-09	–	29410382	Maternal overweight/obesity, Triglycerides in medium HDL	3.20E-08	–	29016858
COL27A1	Nitrogen dioxide exposure	4.90E-09	–	29410382	Maternal overweight/obesity	4.20E-08	–	29016858
EGFR	Nitrogen dioxide exposure	5.00E-09	–	29410382	Maternal overweight/obesity	5.20E-11	–	29016858
TERT	Air pollution exposure, Nitrogen dioxide exposure	5.00E-09	–	26731791, 29410382	Serum triglycerides, Maternal overweight/obesity	1.30E-10	–	28173150, 29016858
LRRC61	Nitrogen dioxide exposure	5.00E-09	–	29410382	Maternal overweight/obesity	1.80E-10	–	29016858

Gene	Trait1	minP	Direction	PMID	Trait2	minP	Direction	PMID
SORCS2	Nitrogen dioxide exposure	5.00E-09	–	29410382	Maternal overweight/obesity, fasting glucose	2.20E-10	–	29016858, 31197173
HECW1	Nitrogen dioxide exposure	5.00E-09	–	29410382	Maternal overweight/obesity	3.40E-09	–	29016858
RGS3	Nitrogen dioxide exposure	5.00E-09	–	29410382	Maternal overweight/obesity, fasting glucose	3.70E-09	–	29016858, 31197173
FAM46B	Air pollution exposure, Nitrogen dioxide exposure	5.00E-09	–	26731791, 29410382	Maternal overweight/obesity	2.00E-08	–	29016858
KCNN3	Nitrogen dioxide exposure	5.10E-09	–	29410382	Maternal overweight/obesity	7.40E-12	–	29016858
IL1F6	Nitrogen dioxide exposure	5.10E-09	–	29410382	Maternal overweight/obesity	3.70E-10	–	29016858
CACNA1D	Nitrogen dioxide exposure	5.10E-09	–	29410382	Maternal overweight/obesity	6.80E-10	–	29016858
SERGEF	Nitrogen dioxide exposure	5.10E-09	–	29410382	Maternal overweight/obesity, Triglycerides	1.40E-09	–	29016858, 31910897
GLIS1	Air pollution exposure, Nitrogen dioxide exposure	5.10E-09	–	26731791, 29410382	Maternal overweight/obesity	1.60E-09	–	29016858
FA2H	Nitrogen dioxide exposure	5.10E-09	–	29410382	Maternal overweight/obesity	3.50E-09	–	29016858
BICC1	Nitrogen dioxide exposure	5.10E-09	–	29410382	Maternal overweight/obesity, 1-hour glucose	1.00E-08	–	29016858, 33151971
CNR1	Nitrogen dioxide exposure	5.10E-09	–	29410382	Maternal overweight/obesity	1.60E-08	–	29016858
C5orf47	Nitrogen dioxide exposure	5.10E-09	–	29410382	Maternal overweight/obesity	1.70E-08	–	29016858

Gene	Trait1	minP	Direction	PMID	Trait2	minP	Direction	PMID
ALDH7A1	Nitrogen dioxide exposure	5.10E-09	–	29410382	Maternal overweight/obesity	2.20E-08	–	29016858
SIPA1L3	Nitrogen dioxide exposure	5.20E-09	–	29410382	Maternal overweight/obesity	1.50E-09	–	29016858
KRT3	Nitrogen dioxide exposure	5.20E-09	–	29410382	Maternal overweight/obesity	3.70E-08	–	29016858
DISC1;TSNAX-DISC1	Nitrogen dioxide exposure	5.30E-09	–	29410382	Maternal overweight/obesity	4.30E-09	–	29016858
SGPL1	Air pollution exposure, Nitrogen dioxide exposure	5.30E-09	–	26731791, 29410382	Maternal overweight/obesity	1.10E-08	–	29016858
KRTAP17-1	Nitrogen dioxide exposure	5.30E-09	–	29410382	Maternal overweight/obesity	2.30E-08	–	29016858
LDLRAP1	Nitrogen dioxide exposure	5.30E-09	+	29410382	fasting glucose, 1-hour glucose	4.30E-08	+	31197173, 33151971
MYH10	Nitrogen dioxide exposure	5.40E-09	–	29410382	Maternal overweight/obesity	9.80E-10	–	29016858
WNT4	Nitrogen dioxide exposure	5.40E-09	–	29410382	Maternal overweight/obesity	1.30E-09	–	29016858
KIAA1530	Air pollution exposure, Nitrogen dioxide exposure	5.40E-09	–	26731791, 29410382	Maternal overweight/obesity, Obesity, Glucose	6.30E-09	–	29016858, 32788176
EGFL8	Nitrogen dioxide exposure	5.50E-09	–	29410382	Maternal overweight/obesity	8.80E-10	–	29016858
TBXAS1	Nitrogen dioxide exposure	5.50E-09	–	29410382	Maternal overweight/obesity, Systolic blood pressure	2.00E-09	–	29016858
COL13A1	Air pollution exposure, Nitrogen dioxide exposure	5.50E-09	–	26731791, 29410382	Maternal overweight/obesity	4.10E-08	–	29016858

Gene	Trait1	minP	Direction	PMID	Trait2	minP	Direction	PMID
SUFU	Nitrogen dioxide exposure	5.60E-09	–	29410382	Maternal overweight/obesity	6.90E-09	–	29016858
ADAM2	Nitrogen dioxide exposure	5.60E-09	–	29410382	Maternal overweight/obesity	1.10E-08	–	29016858
PNLIPRP1	Nitrogen dioxide exposure	5.60E-09	–	29410382	Maternal overweight/obesity	1.20E-08	–	29016858
GEFT	Air pollution exposure, Nitrogen dioxide exposure	5.60E-09	–	26731791, 29410382	Maternal overweight/obesity	1.40E-08	–	29016858
ASAP2	Air pollution exposure, Nitrogen dioxide exposure	5.60E-09	–	26731791, 29410382	Maternal overweight/obesity, Triglycerides in very large HDL	4.00E-08	–	29016858, 32916427
ME3	Nitrogen dioxide exposure	5.60E-09	–	29410382	Maternal overweight/obesity, fasting glucose	4.00E-08	–	29016858, 31197173
PBX1	Nitrogen dioxide exposure	5.60E-09	–	29410382	Maternal overweight/obesity, 1-hour glucose, Diastolic blood pressure	4.80E-08	–	29016858, 33151971
MYO5C	Nitrogen dioxide exposure	5.70E-09	–	29410382	Maternal overweight/obesity, 1-hour glucose	3.00E-10	–	29016858, 33151971
FBXW11	Nitrogen dioxide exposure	5.70E-09	–	29410382	Maternal overweight/obesity, fasting glucose, 1-hour glucose	1.90E-09	–	29016858, 31197173, 33151971

Gene	Trait1	minP	Direction	PMID	Trait2	minP	Direction	PMID
GNAS	Nitrogen dioxide exposure	5.70E-09	+	29410382	Maternal overweight/obesity, Triglycerides to total lipids ratio in large VLDL	2.20E-09	–	29016858
DNMT3L	Nitrogen dioxide exposure	5.70E-09	–	29410382	Maternal overweight/obesity	2.60E-09	–	29016858
BAT5	Nitrogen dioxide exposure	5.70E-09	–	29410382	Maternal overweight/obesity, 1-hour glucose	1.40E-08	–	29016858, 33151971
HSPB7	Nitrogen dioxide exposure	5.80E-09	–	29410382	Maternal overweight/obesity	5.80E-09	–	29016858
PLEKHA7	Nitrogen dioxide exposure	5.80E-09	–	29410382	Maternal overweight/obesity	4.50E-08	–	29016858
MCC	Nitrogen dioxide exposure	5.90E-09	–	29410382	Maternal overweight/obesity, 1-hour glucose	1.20E-10	–	29016858, 33151971
ZBTB20	Nitrogen dioxide exposure	5.90E-09	–	29410382	Maternal overweight/obesity	1.70E-10	–	29016858
C6orf222	Nitrogen dioxide exposure	5.90E-09	–	29410382	Maternal overweight/obesity	3.10E-10	–	29016858
C20orf197	Nitrogen dioxide exposure	5.90E-09	+	29410382	Maternal overweight/obesity	3.80E-10	–	29016858
SPIRE2	Nitrogen dioxide exposure	5.90E-09	–	29410382	Maternal overweight/obesity	4.00E-09	–	29016858
EFNA2	Air pollution exposure, Nitrogen dioxide exposure	5.90E-09	–	26731791, 29410382	Maternal overweight/obesity	2.40E-08	–	29016858
KCNT1	Nitrogen dioxide exposure	6.00E-09	–	29410382	Maternal overweight/obesity	8.40E-11	–	29016858

Gene	Trait1	minP	Direction	PMID	Trait2	minP	Direction	PMID
ATXN7L1	Nitrogen dioxide exposure	6.00E-09	–	29410382	Maternal overweight/obesity, 1-hour glucose	1.30E-09	–	29016858, 33151971
SLC22A12	Nitrogen dioxide exposure	6.00E-09	–	29410382	Maternal overweight/obesity	3.10E-08	–	29016858
TFF3	Nitrogen dioxide exposure	6.10E-09	–	29410382	Maternal overweight/obesity	2.20E-10	–	29016858
XDH	Nitrogen dioxide exposure	6.10E-09	–	29410382	Maternal overweight/obesity	7.60E-09	–	29016858
CSF1R	Nitrogen dioxide exposure	6.10E-09	–	29410382	Maternal overweight/obesity	2.00E-08	–	29016858
SLC38A3	Nitrogen dioxide exposure	6.10E-09	–	29410382	Maternal overweight/obesity, Triglycerides to total lipids ratio in large HDL	2.70E-08	–	29016858
DGKI	Nitrogen dioxide exposure	6.20E-09	–	29410382	Maternal overweight/obesity	1.10E-09	–	29016858
AGRP	Nitrogen dioxide exposure, Particulate matter air pollution	6.20E-09	–	29410382, 32484729	Maternal overweight/obesity	1.90E-08	–	29016858
TSPAN18	Nitrogen dioxide exposure	6.30E-09	–	29410382	Maternal overweight/obesity	2.00E-09	–	29016858, 32916427
CCDC73	Nitrogen dioxide exposure	6.30E-09	–	29410382	Maternal overweight/obesity	1.50E-08	–	29016858
TINAGL1	Nitrogen dioxide exposure	6.30E-09	–	29410382	Maternal overweight/obesity	3.30E-08	–	29016858
PVT1	Air pollution exposure, Nitrogen dioxide exposure	6.30E-09	–	26731791, 29410382	Maternal overweight/obesity	4.70E-08	–	29016858

Gene	Trait1	minP	Direction	PMID	Trait2	minP	Direction	PMID
IGSF21	Nitrogen dioxide exposure	6.40E-09	–	29410382	Maternal overweight/obesity	1.30E-09	–	29016858
RUNX1T1	Nitrogen dioxide exposure	6.40E-09	–	29410382	Maternal overweight/obesity	5.80E-09	–	29016858
GRIK5	Nitrogen dioxide exposure	6.40E-09	–	29410382	Maternal overweight/obesity	5.90E-09	–	29016858
SCN11A	Nitrogen dioxide exposure	6.40E-09	–	29410382	Maternal overweight/obesity	7.50E-09	–	29016858
TRIB3	Nitrogen dioxide exposure	6.40E-09	–	29410382	Maternal overweight/obesity	1.60E-08	–	29016858
KEL	Nitrogen dioxide exposure	6.40E-09	–	29410382	Maternal overweight/obesity	1.60E-08	–	29016858
ACTRT2	Air pollution exposure, Nitrogen dioxide exposure	6.40E-09	–	26731791, 29410382	Maternal overweight/obesity	1.70E-08	–	29016858
CYP26B1	Air pollution exposure, Nitrogen dioxide exposure	6.50E-09	–	26731791, 29410382	Maternal overweight/obesity	4.80E-10	–	29016858
SH2D4B	Air pollution exposure, Nitrogen dioxide exposure	6.50E-09	–	26731791, 29410382	Maternal overweight/obesity	5.90E-09	–	29016858
C1orf200;PIK3CD	Nitrogen dioxide exposure	6.50E-09	+	29410382	Maternal overweight/obesity	1.40E-08	+	29016858
TNNT3	Nitrogen dioxide exposure	6.50E-09	–	29410382	Maternal overweight/obesity	3.80E-08	–	29016858
OR6V1	Nitrogen dioxide exposure	6.60E-09	–	29410382	Maternal overweight/obesity	6.60E-10	–	29016858
METTL11B	Nitrogen dioxide exposure	6.60E-09	–	29410382	Maternal overweight/obesity	1.20E-09	–	29016858

Gene	Trait1	minP	Direction	PMID	Trait2	minP	Direction	PMID
ATP11A	Air pollution exposure, Nitrogen dioxide exposure	6.60E-09	–	26731791, 29410382	Maternal overweight/obesity, 1-hour glucose, Triglycerides in medium LDL, Triglycerides to total lipids ratio in very large VLDL	2.20E-08	–	29016858, 33151971
SLCO2A1	Nitrogen dioxide exposure	6.60E-09	–	29410382	Maternal overweight/obesity	3.10E-08	–	29016858
DENND3	Air pollution exposure, Nitrogen dioxide exposure	6.70E-09	–	26731791, 29410382	Maternal overweight/obesity, Diastolic blood pressure, Triglycerides in large HDL, Triglycerides to total lipids ratio in very large VLDL	5.00E-10	–	29016858
RGL1	Nitrogen dioxide exposure	6.70E-09	–	29410382	Maternal overweight/obesity	1.70E-09	–	29016858
PRKCZ	Air pollution exposure, Nitrogen dioxide exposure, Particulate matter air pollution	6.70E-09	–	26731791, 29410382, 32484729	Maternal overweight/obesity, fasting glucose	4.10E-09	–	29016858, 31197173
MLC1	Nitrogen dioxide exposure	6.70E-09	–	29410382	Maternal overweight/obesity	2.30E-08	–	29016858
LRRK1	Nitrogen dioxide exposure	6.80E-09	–	29410382	Maternal overweight/obesity	4.60E-10	–	29016858
RUNDC2A	Nitrogen dioxide exposure	6.80E-09	–	29410382	Maternal overweight/obesity	1.40E-08	–	29016858

Gene	Trait1	minP	Direction	PMID	Trait2	minP	Direction	PMID
KIAA1199	Air pollution exposure, Nitrogen dioxide exposure	6.90E-09	–	26731791, 29410382	Maternal overweight/obesity, Triglycerides in very large VLDL	7.00E-11	–	29016858
NAA16	Nitrogen dioxide exposure	7.00E-09	+	29410382	Maternal overweight/obesity	4.50E-10	+	29016858
GOLGA3	Nitrogen dioxide exposure	7.00E-09	+	29410382	Maternal overweight/obesity	1.20E-09	+	29016858
AFAP1L2	Nitrogen dioxide exposure	7.00E-09	–	29410382	Maternal overweight/obesity	1.70E-09	–	29016858
PLEKHG5	Nitrogen dioxide exposure	7.00E-09	–	29410382	Maternal overweight/obesity	2.00E-09	–	29016858, 32916427
DNAI2	Nitrogen dioxide exposure	7.00E-09	–	29410382	Maternal overweight/obesity	1.80E-08	–	29016858
B3GAT1	Nitrogen dioxide exposure	7.00E-09	–	29410382	Maternal overweight/obesity, fasting glucose	2.00E-08	–	29016858, 31197173
EHD1	Air pollution exposure, Nitrogen dioxide exposure	7.10E-09	+	26731791, 29410382	Maternal overweight/obesity, Triglycerides in small HDL, Triglycerides in small VLDL, Triglycerides in very small VLDL, Triglycerides to total lipids ratio in medium HDL	1.90E-10	+	29016858
HABP2	Nitrogen dioxide exposure	7.10E-09	–	29410382	Maternal overweight/obesity	2.60E-09	–	29016858
UMODL1	Nitrogen dioxide exposure	7.20E-09	–	29410382	Maternal overweight/obesity	2.30E-10	–	29016858

Gene	Trait1	minP	Direction	PMID	Trait2	minP	Direction	PMID
MEGF6;MIR 551A	Nitrogen dioxide exposure	7.20E-09	–	29410382	Maternal overweight/obesity	2.90E-09	–	29016858
CD59	Nitrogen dioxide exposure	7.20E-09	–	29410382	Maternal overweight/obesity	8.60E-09	–	29016858
BDKRB1	Nitrogen dioxide exposure	7.30E-09	–	29410382	Maternal overweight/obesity	9.20E-10	–	29016858
COL11A1	Nitrogen dioxide exposure	7.30E-09	–	29410382	Maternal overweight/obesity	1.40E-09	–	29016858
MYO16	Nitrogen dioxide exposure	7.30E-09	–	29410382	Maternal overweight/obesity	1.40E-09	–	29016858
CACNA1E	Nitrogen dioxide exposure	7.30E-09	–	29410382	Maternal overweight/obesity	1.50E-08	–	29016858
CNTN2	Nitrogen dioxide exposure	7.30E-09	–	29410382	Maternal overweight/obesity	2.30E-08	–	29016858
FAM69A	Nitrogen dioxide exposure	7.40E-09	–	29410382	Maternal overweight/obesity	4.50E-10	–	29016858
NPHP4	Air pollution exposure, Nitrogen dioxide exposure	7.40E-09	–	26731791, 29410382	Maternal overweight/obesity	1.10E-08	–	29016858
PACRG	Nitrogen dioxide exposure	7.40E-09	–	29410382	Maternal overweight/obesity	3.00E-08	–	29016858
MARCH10	Nitrogen dioxide exposure	7.40E-09	–	29410382	Maternal overweight/obesity	3.50E-08	–	29016858
FGD6	Nitrogen dioxide exposure	7.50E-09	–	29410382	Maternal overweight/obesity	2.50E-08	–	29016858
TPD52L1	Nitrogen dioxide exposure	7.60E-09	–	29410382	Maternal overweight/obesity	2.10E-09	–	29016858
SLC23A3	Nitrogen dioxide exposure	7.60E-09	–	29410382	Maternal overweight/obesity	3.40E-09	–	29016858
CCDC142	Nitrogen dioxide exposure	7.60E-09	–	29410382	Maternal overweight/obesity	7.30E-09	–	29016858

Gene	Trait1	minP	Direction	PMID	Trait2	minP	Direction	PMID
UCHL1	Nitrogen dioxide exposure	7.60E-09	–	29410382	Maternal overweight/obesity	1.00E-08	–	29016858
AADAC	Nitrogen dioxide exposure	7.60E-09	–	29410382	Maternal overweight/obesity	3.60E-08	–	29016858
EDN2	Nitrogen dioxide exposure	7.70E-09	–	29410382	Maternal overweight/obesity	2.10E-10	–	29016858
B3GNTL1	Nitrogen dioxide exposure	7.70E-09	–	29410382	Maternal overweight/obesity, Triglycerides in chylomicrons and extremely large VLDL, Triglycerides in large VLDL	9.40E-09	–	29016858
CRIM1	Nitrogen dioxide exposure	7.80E-09	–	29410382	Maternal overweight/obesity	8.90E-11	–	29016858
PTPRG	Air pollution exposure, Nitrogen dioxide exposure	7.80E-09	–	26731791, 29410382	Maternal overweight/obesity	6.70E-09	–	29016858
SCD5	Nitrogen dioxide exposure	7.90E-09	–	29410382	Maternal overweight/obesity, Triglycerides to total lipids ratio in very small VLDL	8.00E-12	–	29016858
TLL2	Nitrogen dioxide exposure	7.90E-09	–	29410382	Maternal overweight/obesity	7.50E-09	–	29016858
LPP	Nitrogen dioxide exposure	8.00E-09	–	29410382	Maternal overweight/obesity	4.90E-10	–	29016858
PRUNE2	Nitrogen dioxide exposure	8.00E-09	–	29410382	Maternal overweight/obesity	2.30E-08	–	29016858
ENAH	Nitrogen dioxide exposure	8.00E-09	–	29410382	Maternal overweight/obesity, Triglycerides	2.60E-08	–	29016858, 31910897

Gene	Trait1	minP	Direction	PMID	Trait2	minP	Direction	PMID
TNNT2	Nitrogen dioxide exposure	8.00E-09	–	29410382	Maternal overweight/obesity	3.20E-08	–	29016858
CYP11A1	Nitrogen dioxide exposure	8.00E-09	–	29410382	Maternal overweight/obesity	4.30E-08	–	29016858
FAM20C	Nitrogen dioxide exposure	8.10E-09	–	29410382	Maternal overweight/obesity	5.20E-12	–	29016858
PQLC2	Nitrogen dioxide exposure	8.20E-09	–	29410382	Maternal overweight/obesity	1.30E-11	–	29016858
MAP3K10	Nitrogen dioxide exposure	8.20E-09	–	29410382	Maternal overweight/obesity	1.20E-08	–	29016858
PROKR2	Nitrogen dioxide exposure	8.20E-09	–	29410382	Maternal overweight/obesity, Triglycerides, Triglycerides in large VLDL	2.60E-08	–	29016858
RGMA	Nitrogen dioxide exposure	8.30E-09	–	29410382	Maternal overweight/obesity, Triglycerides to total lipids ratio in large LDL, Triglycerides to total lipids ratio in medium LDL, Triglycerides to total lipids ratio in small LDL	9.60E-10	–	29016858
EPOR	Nitrogen dioxide exposure	8.30E-09	–	29410382	Maternal overweight/obesity	2.10E-09	–	29016858
VAC14	Air pollution exposure, Nitrogen dioxide exposure	8.30E-09	–	26731791, 29410382	Maternal overweight/obesity	4.40E-09	–	29016858
C11orf66	Nitrogen dioxide exposure	8.30E-09	–	29410382	Maternal overweight/obesity	6.00E-09	–	29016858

Gene	Trait1	minP	Direction	PMID	Trait2	minP	Direction	PMID
BCL9	Nitrogen dioxide exposure	8.30E-09	–	29410382	1-hour glucose	1.00E-08	+	33151971
HCG26	Nitrogen dioxide exposure	8.40E-09	–	29410382	Maternal overweight/obesity	4.20E-08	–	29016858
ZSWIM4	Nitrogen dioxide exposure	8.50E-09	–	29410382	Maternal overweight/obesity	1.00E-08	–	29016858
KCNG4	Nitrogen dioxide exposure	8.50E-09	–	29410382	Maternal overweight/obesity	1.60E-08	–	29016858
CHST11	Nitrogen dioxide exposure	8.60E-09	+	29410382	Serum triglycerides, Maternal overweight/obesity	3.60E-10	+	28213390, 29016858
CMTM5	Nitrogen dioxide exposure	8.60E-09	–	29410382	Maternal overweight/obesity	2.00E-08	–	29016858
MFN2	Nitrogen dioxide exposure	8.60E-09	–	29410382	Maternal overweight/obesity	3.60E-08	–	29016858
TRNP1	Nitrogen dioxide exposure	8.60E-09	–	29410382	Maternal overweight/obesity	3.70E-08	–	29016858
C10orf54;CDH23	Nitrogen dioxide exposure	8.70E-09	+	29410382	Maternal overweight/obesity	1.90E-09	+	29016858
SLC5A9	Nitrogen dioxide exposure	8.70E-09	–	29410382	Maternal overweight/obesity	7.10E-09	–	29016858, 32916427
EXOC4	Nitrogen dioxide exposure	8.70E-09	–	29410382	Maternal overweight/obesity	1.70E-08	–	29016858
KIAA1274	Nitrogen dioxide exposure	8.70E-09	–	29410382	Maternal overweight/obesity, fasting glucose	2.90E-08	–	29016858, 31197173
ARRDC5	Nitrogen dioxide exposure	8.80E-09	–	29410382	Maternal overweight/obesity	1.10E-08	–	29016858
S100A16	Nitrogen dioxide exposure	8.80E-09	–	29410382	Maternal overweight/obesity	3.60E-08	–	29016858

Gene	Trait1	minP	Direction	PMID	Trait2	minP	Direction	PMID
PITPNM1	Nitrogen dioxide exposure	8.80E-09	–	29410382	Maternal overweight/obesity	4.30E-08	+	29016858
MUC2	Nitrogen dioxide exposure	8.80E-09	–	29410382	Maternal overweight/obesity	4.50E-08	–	29016858
MBNL2	Nitrogen dioxide exposure	8.90E-09	–	29410382	Maternal overweight/obesity	2.10E-09	–	29016858
LOC400752	Nitrogen dioxide exposure	8.90E-09	–	29410382	Maternal overweight/obesity	2.60E-08	–	29016858
INPP4B	Nitrogen dioxide exposure	9.00E-09	–	29410382	Maternal overweight/obesity	4.90E-09	–	29016858
CAMK2A	Nitrogen dioxide exposure	9.10E-09	–	29410382	Maternal overweight/obesity	2.00E-08	–	29016858
C20orf26	Nitrogen dioxide exposure	9.20E-09	–	29410382	Maternal overweight/obesity	1.30E-10	–	29016858
GALNT9	Nitrogen dioxide exposure	9.20E-09	–	29410382	Maternal overweight/obesity, Triglycerides to total lipids ratio in medium VLDL	2.20E-10	–	29016858
PPP2R2C	Nitrogen dioxide exposure	9.20E-09	–	29410382	Maternal overweight/obesity, 2-hour glucose	6.70E-09	–	29016858, 33151971
GRB7	Nitrogen dioxide exposure	9.20E-09	–	29410382	Maternal overweight/obesity	3.10E-08	–	29016858
IL4I1	Nitrogen dioxide exposure	9.20E-09	–	29410382	Maternal overweight/obesity	3.60E-08	–	29016858
DUOXA1	Nitrogen dioxide exposure	9.30E-09	–	29410382	Maternal overweight/obesity	1.40E-08	–	29016858
LOC286135	Nitrogen dioxide exposure	9.40E-09	–	29410382	Maternal overweight/obesity	4.50E-10	–	29016858
CETN1	Nitrogen dioxide exposure	9.40E-09	–	29410382	Maternal overweight/obesity	1.00E-09	–	29016858

Gene	Trait1	minP	Direction	PMID	Trait2	minP	Direction	PMID
PGLYRP2	Nitrogen dioxide exposure	9.40E-09	–	29410382	Maternal overweight/obesity	5.30E-09	–	29016858
ACCN4	Air pollution exposure, Nitrogen dioxide exposure	9.50E-09	–	26731791, 29410382	Maternal overweight/obesity	1.20E-10	–	29016858
CCNL2	Nitrogen dioxide exposure	9.50E-09	–	29410382	Maternal overweight/obesity	2.40E-09	–	29016858
KCNH2	Nitrogen dioxide exposure	9.60E-09	–	29410382	Maternal overweight/obesity	1.80E-12	–	29016858
PHC2	Nitrogen dioxide exposure	9.60E-09	–	29410382	Maternal overweight/obesity	1.10E-08	–	29016858
MAML2	Nitrogen dioxide exposure	9.60E-09	–	29410382	Maternal overweight/obesity, 1-hour glucose	2.30E-08	–	29016858, 33151971
KRT18	Nitrogen dioxide exposure	9.60E-09	–	29410382	Maternal overweight/obesity	2.60E-08	–	29016858
ST5	Air pollution exposure, Nitrogen dioxide exposure	9.80E-09	–	26731791, 29410382	Maternal overweight/obesity, Triglycerides in small HDL, Triglycerides to total lipids ratio in small HDL	4.30E-08	–	29016858
PTCRA	Nitrogen dioxide exposure	9.90E-09	–	29410382	Maternal overweight/obesity	2.90E-12	–	29016858
ATP2A3	Nitrogen dioxide exposure	9.90E-09	–	29410382	Maternal overweight/obesity	8.00E-11	–	29016858
PXDN	Nitrogen dioxide exposure	9.90E-09	–	29410382	Maternal overweight/obesity	1.40E-09	–	29016858
ALDH8A1	Air pollution exposure, Nitrogen dioxide exposure	1.00E-08	–	26731791, 29410382	Maternal overweight/obesity	7.10E-09	–	29016858

Gene	Trait1	minP	Direction	PMID	Trait2	minP	Direction	PMID
AQP6	Nitrogen dioxide exposure	1.00E-08	–	29410382	Maternal overweight/obesity	9.40E-09	–	29016858
RBM47	Air pollution exposure, Nitrogen dioxide exposure	1.00E-08	–	26731791, 29410382	Maternal overweight/obesity	2.60E-08	–	29016858
HLA-DOA	Nitrogen dioxide exposure	1.00E-08	–	29410382	Maternal overweight/obesity	3.40E-08	–	29016858
RAB11FIP4	Nitrogen dioxide exposure	1.00E-08	–	29410382	Maternal overweight/obesity	3.40E-08	–	29016858
MTNR1B	Nitrogen dioxide exposure	1.00E-08	–	29410382	Maternal overweight/obesity	4.00E-08	–	29016858
EPHB3	Nitrogen dioxide exposure	1.10E-08	–	29410382	Maternal overweight/obesity	2.20E-14	–	29016858
DAO	Nitrogen dioxide exposure	1.10E-08	–	29410382	Maternal overweight/obesity	4.20E-11	–	29016858
BRSK2	Nitrogen dioxide exposure	1.10E-08	–	29410382	Maternal overweight/obesity	7.50E-11	–	29016858
FLI1	Nitrogen dioxide exposure	1.10E-08	–	29410382	Maternal overweight/obesity, Diastolic Blood Pressure, Systolic Blood Pressure	6.20E-10	–	29016858, 29198723
FLT4	Nitrogen dioxide exposure	1.10E-08	–	29410382	Maternal overweight/obesity	1.70E-09	+	29016858
ABTB2	Nitrogen dioxide exposure	1.10E-08	–	29410382	Maternal overweight/obesity	1.90E-09	–	29016858
FLRT1;MACROD1	Nitrogen dioxide exposure	1.10E-08	–	29410382	Maternal overweight/obesity	2.40E-09	–	29016858
RYR1	Nitrogen dioxide exposure	1.10E-08	–	29410382	Maternal overweight/obesity, Triglycerides to total lipids ratio in large HDL	2.50E-09	–	29016858

Gene	Trait1	minP	Direction	PMID	Trait2	minP	Direction	PMID
DARC	Nitrogen dioxide exposure	1.10E-08	–	29410382	Maternal overweight/obesity	4.00E-09	–	29016858
C11orf41	Nitrogen dioxide exposure	1.10E-08	–	29410382	Maternal overweight/obesity	6.10E-09	–	29016858
PCDHGA4; PCDHGA11; PCDHGA9; PCDHGA1; PCDHGB1; PCDHGB8P; PCDHGB6; PCDHGB3; PCDHGB7; PCDHGA6; PCDHGA8; PCDHGA10; PCDHGA5; PCDHGB4; PCDHGA3; PCDHGA2; PCDHGB2; PCDHGA7; PCDHGB5	Nitrogen dioxide exposure	1.10E-08	–	29410382	Maternal overweight/obesity	9.80E-09	–	29016858
HEXDC	Air pollution exposure, Nitrogen dioxide exposure	1.10E-08	–	26731791, 29410382	Maternal overweight/obesity	1.30E-08	–	29016858
ZNF862	Nitrogen dioxide exposure	1.10E-08	–	29410382	Maternal overweight/obesity	1.30E-08	–	29016858
PSCA	Nitrogen dioxide exposure	1.10E-08	–	29410382	Maternal overweight/obesity	1.40E-08	–	29016858

Gene	Trait1	minP	Direction	PMID	Trait2	minP	Direction	PMID
FAM108C1	Nitrogen dioxide exposure	1.10E-08	–	29410382	Maternal overweight/obesity	1.40E-08	–	29016858
OR4D10	Nitrogen dioxide exposure	1.10E-08	–	29410382	Maternal overweight/obesity	1.80E-08	–	29016858
SRGAP2	Nitrogen dioxide exposure	1.10E-08	–	29410382	Maternal overweight/obesity	2.10E-08	–	29016858
FAM135A	Nitrogen dioxide exposure	1.10E-08	–	29410382	Maternal overweight/obesity	4.50E-08	–	29016858
ATP8B1	Nitrogen dioxide exposure	1.20E-08	–	29410382	Maternal overweight/obesity	5.80E-11	–	29016858
ADCY3	Nitrogen dioxide exposure	1.20E-08	–	29410382	Maternal overweight/obesity	7.10E-11	–	29016858
LRRN2	Nitrogen dioxide exposure	1.20E-08	–	29410382	Maternal overweight/obesity	1.60E-10	–	29016858
CSF2	Nitrogen dioxide exposure	1.20E-08	–	29410382	Maternal overweight/obesity, fasting glucose	2.70E-10	–	29016858, 31197173
C1orf203	Nitrogen dioxide exposure	1.20E-08	–	29410382	Maternal overweight/obesity	3.30E-10	–	29016858
CASZ1	Nitrogen dioxide exposure	1.20E-08	–	29410382	Maternal overweight/obesity, Triglycerides in very small VLDL	3.50E-10	–	29016858
LOC642587; MIR205	Nitrogen dioxide exposure	1.20E-08	–	29410382	Maternal overweight/obesity	5.40E-10	–	29016858
KIAA1609	Nitrogen dioxide exposure	1.20E-08	–	29410382	Maternal overweight/obesity	7.60E-10	–	29016858
MET	Nitrogen dioxide exposure	1.20E-08	–	29410382	Maternal overweight/obesity	1.40E-09	–	29016858
ZNF365	Nitrogen dioxide exposure	1.20E-08	–	29410382	Maternal overweight/obesity	2.30E-09	–	29016858

Gene	Trait1	minP	Direction	PMID	Trait2	minP	Direction	PMID
SLC39A4	Nitrogen dioxide exposure	1.20E-08	–	29410382	Maternal overweight/obesity	2.70E-09	–	29016858
OR4D11	Nitrogen dioxide exposure	1.20E-08	–	29410382	Maternal overweight/obesity	3.60E-09	–	29016858
A2BP1	Nitrogen dioxide exposure	1.20E-08	–	29410382	Maternal overweight/obesity, Triglycerides to total lipids ratio in large LDL	4.80E-09	–	29016858
AKT1S1	Nitrogen dioxide exposure	1.20E-08	–	29410382	Maternal overweight/obesity	5.70E-09	–	29016858
MGC26597	Nitrogen dioxide exposure	1.20E-08	–	29410382	Maternal overweight/obesity	6.20E-09	–	29016858
BEGAIN	Nitrogen dioxide exposure	1.20E-08	–	29410382	Maternal overweight/obesity	9.80E-09	–	29016858
OMP;CAPN5	Nitrogen dioxide exposure	1.20E-08	–	29410382	Maternal overweight/obesity	1.30E-08	–	29016858
COL5A1	Air pollution exposure, Nitrogen dioxide exposure	1.20E-08	–	26731791, 29410382	Maternal overweight/obesity	1.70E-08	–	29016858
TMEM211	Nitrogen dioxide exposure	1.20E-08	–	29410382	Maternal overweight/obesity	3.10E-08	–	29016858
GDNF	Nitrogen dioxide exposure	1.20E-08	–	29410382	Maternal overweight/obesity	3.40E-08	–	29016858
RAD51L1	Nitrogen dioxide exposure	1.30E-08	–	29410382	Maternal overweight/obesity	1.60E-10	–	29016858
TH	Nitrogen dioxide exposure	1.30E-08	–	29410382	Maternal overweight/obesity	1.80E-09	–	29016858
SEMA5B	Nitrogen dioxide exposure	1.30E-08	–	29410382	Maternal overweight/obesity	2.30E-09	–	29016858
C19orf21	Nitrogen dioxide exposure	1.30E-08	–	29410382	Maternal overweight/obesity	8.60E-09	–	29016858

Gene	Trait1	minP	Direction	PMID	Trait2	minP	Direction	PMID
SLC29A4	Nitrogen dioxide exposure	1.30E-08	–	29410382	Maternal overweight/obesity	8.90E-09	–	29016858
LIMCH1	Nitrogen dioxide exposure	1.30E-08	–	29410382	Maternal overweight/obesity	1.30E-08	–	29016858
ADAM8	Nitrogen dioxide exposure	1.30E-08	–	29410382	Maternal overweight/obesity, Diastolic Blood Pressure, Systolic Blood Pressure, fasting glucose	1.50E-08	–	29016858, 29198723, 31197173
ZNF385A	Nitrogen dioxide exposure	1.30E-08	–	29410382	Maternal overweight/obesity	1.50E-08	–	29016858
SHF	Nitrogen dioxide exposure	1.30E-08	–	29410382	Maternal overweight/obesity	2.20E-08	–	29016858
LOC407835	Nitrogen dioxide exposure	1.30E-08	–	29410382	Maternal overweight/obesity	2.30E-08	–	29016858
FXYD5	Nitrogen dioxide exposure	1.40E-08	–	29410382	Maternal overweight/obesity	9.40E-12	–	29016858
C1QC	Nitrogen dioxide exposure	1.40E-08	–	29410382	Maternal overweight/obesity	3.20E-10	–	29016858
SEMA3B	Air pollution exposure, Nitrogen dioxide exposure	1.40E-08	–	26731791, 29410382	Maternal overweight/obesity, Triglycerides to total lipids ratio in very large VLDL	3.90E-10	–	29016858
ASAM	Nitrogen dioxide exposure	1.40E-08	–	29410382	Maternal overweight/obesity	6.30E-10	–	29016858
PTMS	Nitrogen dioxide exposure	1.40E-08	–	29410382	Maternal overweight/obesity	2.80E-09	–	29016858

Gene	Trait1	minP	Direction	PMID	Trait2	minP	Direction	PMID
LRP5	Nitrogen dioxide exposure	1.40E-08	–	29410382	Maternal overweight/obesity, Triglycerides in HDL, Triglycerides to total lipids ratio in IDL, Triglycerides to total lipids ratio in large HDL, Triglycerides to total lipids ratio in very large HDL	1.50E-08	–	29016858
CAPZB	Nitrogen dioxide exposure, Particulate matter <2.5um (PM2.5)	1.40E-08	–	29410382	Maternal overweight/obesity, 1-hour glucose	1.80E-08	–	29016858, 33151971
CNIH3	Air pollution exposure, Nitrogen dioxide exposure	1.40E-08	–	26731791, 29410382	Maternal overweight/obesity	1.90E-08	–	29016858
UBASH3B	Nitrogen dioxide exposure	1.40E-08	–	29410382	Maternal overweight/obesity	2.20E-08	–	29016858
NCALD	Nitrogen dioxide exposure	1.40E-08	–	29410382	Maternal overweight/obesity	2.40E-08	–	29016858
YAP1	Nitrogen dioxide exposure	1.40E-08	–	29410382	Maternal overweight/obesity	3.10E-08	–	29016858
SEMA4F	Nitrogen dioxide exposure	1.40E-08	–	29410382	Maternal overweight/obesity	3.20E-08	–	29016858
NOSIP	Nitrogen dioxide exposure	1.40E-08	+	29410382	Maternal overweight/obesity, Triglycerides in large HDL	3.40E-08	+	29016858
TCF3	Air pollution exposure, Nitrogen dioxide exposure	1.40E-08	–	26731791, 29410382	Maternal overweight/obesity	4.10E-08	–	29016858

Gene	Trait1	minP	Direction	PMID	Trait2	minP	Direction	PMID
RBPMS	Nitrogen dioxide exposure	1.50E-08	–	29410382	Maternal obesity, Maternal overweight/obesity	9.10E-12	–	25855720, 29016858
ANKRD11	Nitrogen dioxide exposure	1.50E-08	–	29410382	Maternal overweight/obesity, Diastolic Blood Pressure, Systolic Blood Pressure, fasting glucose, 1-hour glucose, Ratio of triglycerides to phosphoglycerides, Triglycerides, Triglycerides in large VLDL	2.50E-10	–	29016858, 29198723, 31197173, 33151971
GP1BA	Nitrogen dioxide exposure	1.50E-08	–	29410382	Maternal overweight/obesity	3.90E-10	–	29016858
B3GNT3	Nitrogen dioxide exposure	1.50E-08	–	29410382	Maternal overweight/obesity, 1-hour glucose	2.40E-09	–	29016858, 33151971
TMEM86A	Nitrogen dioxide exposure	1.50E-08	–	29410382	Maternal overweight/obesity	3.60E-09	–	29016858
ZNF692	Nitrogen dioxide exposure	1.50E-08	–	29410382	Maternal overweight/obesity, Triglycerides in large HDL	5.90E-09	+	29016858
FAM189A1	Nitrogen dioxide exposure	1.50E-08	–	29410382	Maternal overweight/obesity	8.40E-09	–	29016858
WFDC10B; WFDC13	Nitrogen dioxide exposure	1.50E-08	–	29410382	Maternal overweight/obesity	1.00E-08	–	29016858

Gene	Trait1	minP	Direction	PMID	Trait2	minP	Direction	PMID
RPTOR	Air pollution exposure, Prenatal NO2 exposure, Nitrogen dioxide exposure	1.50E-08	–	26731791, 27448387, 29410382	Maternal overweight/obesity, 1-hour glucose, Triglycerides in IDL, Triglycerides in large LDL, Triglycerides to total lipids ratio in large HDL, Triglycerides to total lipids ratio in very large HDL	2.30E-08	–	29016858, 33151971
CPE;MIR1979	Nitrogen dioxide exposure	1.50E-08	–	29410382	Maternal overweight/obesity	2.40E-08	–	29016858
ARID3A	Nitrogen dioxide exposure	1.50E-08	–	29410382	Maternal overweight/obesity, 1-hour glucose	2.70E-08	–	29016858, 33151971
EPHX1	Nitrogen dioxide exposure	1.50E-08	–	29410382	Maternal overweight/obesity	2.90E-08	–	29016858
KCNJ16	Nitrogen dioxide exposure	1.50E-08	–	29410382	Maternal overweight/obesity	4.30E-08	–	29016858
LRRC31	Nitrogen dioxide exposure	1.50E-08	–	29410382	Maternal overweight/obesity	4.60E-08	–	29016858
DDR1	Nitrogen dioxide exposure	1.60E-08	–	29410382	Maternal overweight/obesity	1.50E-12	–	29016858
ZMIZ1	Air pollution exposure, Nitrogen dioxide exposure	1.60E-08	–	26731791, 29410382	Diastolic Blood Pressure, Systolic Blood Pressure, Triglycerides, Triglycerides in small HDL	2.00E-11	–	29198723

Gene	Trait1	minP	Direction	PMID	Trait2	minP	Direction	PMID
KIAA0146	Nitrogen dioxide exposure	1.60E-08	–	29410382	Maternal overweight/obesity, Ratio of triglycerides to phosphoglycerides, Triglycerides in large VLDL, Triglycerides in very large VLDL	4.40E-11	–	29016858
FSCN1	Nitrogen dioxide exposure	1.60E-08	–	29410382	Maternal overweight/obesity	5.10E-11	–	29016858
C3orf52;MIR 567	Nitrogen dioxide exposure	1.60E-08	–	29410382	Maternal overweight/obesity	1.70E-09	–	29016858
SEPN1	Nitrogen dioxide exposure	1.60E-08	–	29410382	Maternal overweight/obesity	1.70E-09	–	29016858
SMOC2	Nitrogen dioxide exposure	1.60E-08	–	29410382	Maternal overweight/obesity, Triglycerides in chylomicrons and extremely large VLDL	4.00E-09	–	29016858
MTMR11	Nitrogen dioxide exposure	1.60E-08	–	29410382	Maternal overweight/obesity	5.10E-09	–	29016858
PIK3CD	Nitrogen dioxide exposure	1.60E-08	–	29410382	Maternal overweight/obesity, Triglycerides in small HDL	1.10E-08	–	29016858
DOCK2	Nitrogen dioxide exposure	1.60E-08	–	29410382	Maternal overweight/obesity	1.40E-08	–	29016858
WDR27	Nitrogen dioxide exposure	1.60E-08	–	29410382	Maternal overweight/obesity	1.60E-08	–	29016858
LFNG	Nitrogen dioxide exposure	1.60E-08	–	29410382	Maternal overweight/obesity	3.20E-08	–	29016858

Gene	Trait1	minP	Direction	PMID	Trait2	minP	Direction	PMID
RNLS	Nitrogen dioxide exposure	1.60E-08	–	29410382	Maternal overweight/obesity, Triglycerides, Ratio of triglycerides to phosphoglycerides, Triglycerides in chylomicrons and extremely large VLDL	3.30E-08	–	29016858, 31910897
KIF26B	Nitrogen dioxide exposure	1.60E-08	–	29410382	Maternal overweight/obesity, Triglycerides to total lipids ratio in medium HDL, Triglycerides to total lipids ratio in small HDL	3.80E-08	–	29016858
CDH29	Nitrogen dioxide exposure	1.70E-08	–	29410382	Maternal overweight/obesity	3.50E-10	–	29016858
HGFAC	Nitrogen dioxide exposure	1.70E-08	–	29410382	Maternal overweight/obesity	2.10E-09	–	29016858
TNC	Nitrogen dioxide exposure	1.70E-08	–	29410382	Maternal overweight/obesity	5.40E-09	–	29016858
TLN2	Nitrogen dioxide exposure	1.70E-08	–	29410382	Maternal overweight/obesity	5.40E-09	–	29016858
TRIM50	Nitrogen dioxide exposure	1.70E-08	–	29410382	Maternal overweight/obesity	1.00E-08	–	29016858
ADAD1	Nitrogen dioxide exposure	1.70E-08	–	29410382	Maternal overweight/obesity	1.90E-08	–	29016858
ITGA7	Nitrogen dioxide exposure	1.70E-08	–	29410382	Maternal overweight/obesity	3.00E-08	–	29016858
TRPM2	Nitrogen dioxide exposure	1.70E-08	–	29410382	Maternal overweight/obesity, Glucose	4.00E-08	–	29016858

Gene	Trait1	minP	Direction	PMID	Trait2	minP	Direction	PMID
COBL	Nitrogen dioxide exposure	1.70E-08	–	29410382	Maternal overweight/obesity, 1-hour glucose	4.00E-08	–	29016858, 33151971
HTA	Nitrogen dioxide exposure	1.70E-08	–	29410382	Maternal overweight/obesity	4.10E-08	–	29016858
MYOF	Nitrogen dioxide exposure	1.70E-08	–	29410382	Maternal overweight/obesity, 1-hour glucose	4.20E-08	–	29016858, 33151971
AIM1L	Nitrogen dioxide exposure	1.70E-08	–	29410382	Maternal overweight/obesity	4.30E-08	–	29016858
BLVRA	Nitrogen dioxide exposure	1.70E-08	–	29410382	Maternal overweight/obesity	4.60E-08	–	29016858
NSD1	Nitrogen dioxide exposure	1.70E-08	–	29410382	Maternal overweight/obesity	4.60E-08	–	29016858
GPR78	Nitrogen dioxide exposure	1.70E-08	–	29410382	Maternal overweight/obesity, fasting glucose	4.90E-08	–	29016858, 31197173
LOC100188949	Nitrogen dioxide exposure	1.80E-08	+	29410382	Maternal overweight/obesity	1.40E-11	+	29016858
FBXO10	Nitrogen dioxide exposure	1.80E-08	–	29410382	Maternal overweight/obesity	1.10E-10	–	29016858
AGPAT1	Air pollution exposure, Nitrogen dioxide exposure	1.80E-08	–	26731791, 29410382	Maternal overweight/obesity	3.00E-10	–	29016858
SCGB1A1	Nitrogen dioxide exposure	1.80E-08	–	29410382	Maternal overweight/obesity	9.00E-10	–	29016858
ROBO4	Nitrogen dioxide exposure	1.80E-08	–	29410382	Maternal overweight/obesity	1.20E-09	–	29016858
UBE2U	Nitrogen dioxide exposure	1.80E-08	–	29410382	Maternal overweight/obesity	2.10E-09	–	29016858
H1FOO	Nitrogen dioxide exposure	1.80E-08	–	29410382	Maternal overweight/obesity	3.00E-09	–	29016858

Gene	Trait1	minP	Direction	PMID	Trait2	minP	Direction	PMID
SPTB	Nitrogen dioxide exposure	1.80E-08	–	29410382	fasting glucose	3.20E-09	–	31197173
C10orf18	Nitrogen dioxide exposure	1.80E-08	+	29410382	Maternal overweight/obesity	7.20E-09	+	29016858
ITGB7	Air pollution exposure, Nitrogen dioxide exposure	1.80E-08	+	26731791, 29410382	Maternal overweight/obesity	1.20E-08	–	29016858
DPYSL2	Nitrogen dioxide exposure	1.80E-08	–	29410382	Maternal overweight/obesity	1.30E-08	–	29016858
GJB4	Prenatal NO2 exposure, Nitrogen dioxide exposure	1.80E-08	–	27448387, 29410382	Maternal overweight/obesity	1.50E-08	–	29016858
PPP1R1A	Nitrogen dioxide exposure	1.80E-08	–	29410382	Maternal overweight/obesity	2.20E-08	–	29016858
LHPP	Air pollution exposure, Nitrogen dioxide exposure	1.80E-08	–	26731791, 29410382	Maternal overweight/obesity	3.00E-08	–	29016858
TSPYL6;ACYP2	Air pollution exposure, Nitrogen dioxide exposure	1.86E-08	+	26731791, 29410382	Maternal overweight/obesity	1.90E-11	–	29016858
WIPF1	Nitrogen dioxide exposure	1.90E-08	+	29410382	Maternal overweight/obesity	6.60E-10	+	29016858
CAMP	Nitrogen dioxide exposure	1.90E-08	–	29410382	Maternal overweight/obesity	3.90E-09	+	29016858
GABRD	Nitrogen dioxide exposure	1.90E-08	–	29410382	Maternal overweight/obesity	6.30E-09	–	29016858
SLC45A4	Air pollution exposure, Nitrogen dioxide exposure	1.90E-08	–	26731791, 29410382	Maternal overweight/obesity, 1-hour glucose	1.20E-08	–	29016858, 33151971
VPS52	Nitrogen dioxide exposure	1.90E-08	–	29410382	Maternal overweight/obesity	1.20E-08	–	29016858

Gene	Trait1	minP	Direction	PMID	Trait2	minP	Direction	PMID
C7orf41	Nitrogen dioxide exposure	1.90E-08	–	29410382	Maternal overweight/obesity	1.30E-08	–	29016858
RABGGTA	Nitrogen dioxide exposure	1.90E-08	–	29410382	Maternal overweight/obesity	1.70E-08	–	29016858
CCDC40	Nitrogen dioxide exposure	2.00E-08	–	29410382	Maternal overweight/obesity	2.10E-10	–	29016858
MVP	Air pollution exposure, Nitrogen dioxide exposure	2.00E-08	–	26731791, 29410382	Maternal overweight/obesity	9.40E-10	–	29016858
MEG3	Nitrogen dioxide exposure	2.00E-08	–	29410382	Maternal overweight/obesity	1.10E-09	–	29016858
HPX	Nitrogen dioxide exposure	2.00E-08	–	29410382	Maternal overweight/obesity	1.70E-09	–	29016858
CDH13	Nitrogen dioxide exposure	2.00E-08	–	29410382	Maternal overweight/obesity, fasting glucose	4.30E-09	–	29016858, 31197173
SERPINA6	Nitrogen dioxide exposure	2.00E-08	–	29410382	Maternal overweight/obesity	4.60E-09	–	29016858
KSR1	Nitrogen dioxide exposure	2.00E-08	–	29410382	Maternal overweight/obesity	3.90E-08	–	29016858
CUX1	Air pollution exposure, Nitrogen dioxide exposure	2.00E-08	–	26731791, 29410382	Maternal overweight/obesity	4.80E-08	–	29016858
EFNA1	Nitrogen dioxide exposure	2.10E-08	–	29410382	Maternal overweight/obesity	8.70E-10	–	29016858
SAMD5	Nitrogen dioxide exposure	2.10E-08	–	29410382	Maternal overweight/obesity	1.10E-09	–	29016858
C14orf180	Nitrogen dioxide exposure	2.10E-08	–	29410382	Maternal overweight/obesity	4.00E-09	–	29016858
PACSIN1	Air pollution exposure, Nitrogen dioxide exposure	2.10E-08	–	26731791, 29410382	Maternal overweight/obesity, 1-hour glucose	1.30E-08	–	29016858, 33151971

Gene	Trait1	minP	Direction	PMID	Trait2	minP	Direction	PMID
GPR35	Nitrogen dioxide exposure	2.10E-08	–	29410382	Maternal overweight/obesity	2.50E-08	–	29016858
SEMA6B	Nitrogen dioxide exposure	2.10E-08	–	29410382	Maternal overweight/obesity	3.00E-08	–	29016858
GPR113	Nitrogen dioxide exposure	2.10E-08	–	29410382	Maternal overweight/obesity	4.20E-08	–	29016858
FBXO2	Nitrogen dioxide exposure	2.10E-08	–	29410382	Maternal overweight/obesity	4.80E-08	–	29016858
TBL3	Air pollution exposure, Nitrogen dioxide exposure	2.20E-08	–	26731791, 29410382	Change in maternal diastolic blood pressure across pregnancy, Change in maternal diastolic blood pressure between second and third trimester, Change in maternal systolic blood pressure across pregnancy, Change in maternal systolic blood pressure between second and third trimester, Maternal diastolic blood pressure, Maternal systolic blood pressure	1.70E-15	NA	32078381
CSTT	Nitrogen dioxide exposure	2.20E-08	–	29410382	Maternal overweight/obesity	3.60E-11	–	29016858
TTC13	Nitrogen dioxide exposure	2.20E-08	–	29410382	Maternal overweight/obesity, 1-hour glucose	1.20E-10	–	29016858, 33151971

Gene	Trait1	minP	Direction	PMID	Trait2	minP	Direction	PMID
CDYL2	Nitrogen dioxide exposure	2.20E-08	–	29410382	Maternal overweight/obesity	2.40E-10	–	29016858
MMP14	Nitrogen dioxide exposure	2.20E-08	–	29410382	Maternal overweight/obesity	2.50E-10	–	29016858
H1F0	Nitrogen dioxide exposure	2.20E-08	–	29410382	Maternal overweight/obesity, Diastolic Blood Pressure, Systolic Blood Pressure	6.00E-10	–	29016858, 29198723
A2ML1	Nitrogen dioxide exposure	2.20E-08	–	29410382	Maternal overweight/obesity	7.60E-10	–	29016858
LYN	Nitrogen dioxide exposure	2.20E-08	+	29410382	Maternal overweight/obesity	1.50E-09	+	29016858
C1orf159	Air pollution exposure, Nitrogen dioxide exposure	2.20E-08	–	26731791, 29410382	Maternal overweight/obesity	3.80E-09	–	29016858
HOXB1	Nitrogen dioxide exposure	2.20E-08	–	29410382	Maternal overweight/obesity	2.10E-08	–	29016858
CACNA1B	Nitrogen dioxide exposure	2.20E-08	–	29410382	Maternal overweight/obesity	2.90E-08	–	29016858
ADCY5	Nitrogen dioxide exposure	2.20E-08	–	29410382	Maternal overweight/obesity, 1-hour glucose	3.70E-08	–	29016858, 33151971
KIF2B	Nitrogen dioxide exposure	2.20E-08	–	29410382	Maternal overweight/obesity	4.30E-08	–	29016858
NTN5;SEC1	Nitrogen dioxide exposure	2.30E-08	–	29410382	Maternal overweight/obesity	2.10E-11	–	29016858
CHRNB2	Nitrogen dioxide exposure	2.30E-08	–	29410382	Maternal overweight/obesity	1.80E-09	–	29016858
SYN2	Nitrogen dioxide exposure	2.30E-08	–	29410382	Maternal overweight/obesity	4.10E-09	–	29016858
DLC1	Nitrogen dioxide exposure	2.30E-08	–	29410382	Maternal overweight/obesity	6.10E-09	–	29016858

Gene	Trait1	minP	Direction	PMID	Trait2	minP	Direction	PMID
DHCR7	Nitrogen dioxide exposure	2.30E-08	–	29410382	Maternal overweight/obesity	7.90E-09	–	29016858
GJA5	Nitrogen dioxide exposure	2.30E-08	–	29410382	Maternal overweight/obesity	8.00E-09	–	29016858
AJAP1	Nitrogen dioxide exposure	2.30E-08	–	29410382	Maternal overweight/obesity, Triglycerides	9.70E-09	–	29016858, 31910897, 32916427
DHDPSL	Nitrogen dioxide exposure	2.30E-08	–	29410382	Maternal overweight/obesity	2.40E-08	–	29016858
C14orf73	Air pollution exposure, Nitrogen dioxide exposure	2.30E-08	–	26731791, 29410382	Maternal overweight/obesity	2.90E-08	–	29016858
EXPH5	Nitrogen dioxide exposure	2.40E-08	–	29410382	Maternal overweight/obesity	4.30E-15	–	29016858
MFAP2	Nitrogen dioxide exposure	2.40E-08	–	29410382	Maternal overweight/obesity	2.80E-10	–	29016858
HLA-L	Nitrogen dioxide exposure	2.40E-08	–	29410382	Maternal overweight/obesity	7.40E-10	–	29016858
WISP3	Nitrogen dioxide exposure	2.40E-08	–	29410382	Maternal overweight/obesity	3.00E-09	–	29016858
LRP1	Air pollution exposure, Nitrogen dioxide exposure	2.40E-08	–	26731791, 29410382	Maternal overweight/obesity	3.40E-09	–	29016858
TTC38	Nitrogen dioxide exposure	2.40E-08	–	29410382	Maternal overweight/obesity, fasting glucose	4.30E-09	–	29016858, 31197173
AMZ1	Air pollution exposure, Nitrogen dioxide exposure	2.40E-08	–	26731791, 29410382	Maternal overweight/obesity	7.70E-09	–	29016858

Gene	Trait1	minP	Direction	PMID	Trait2	minP	Direction	PMID
FAM129B	Nitrogen dioxide exposure	2.40E-08	–	29410382	Maternal obesity	1.39E-08	–	25855720
TGM6	Nitrogen dioxide exposure	2.40E-08	–	29410382	Maternal overweight/obesity	1.40E-08	–	29016858
KRT35	Nitrogen dioxide exposure	2.40E-08	–	29410382	Maternal overweight/obesity	2.70E-08	–	29016858
BPHL	Air pollution exposure, Nitrogen dioxide exposure	2.40E-08	–	26731791, 29410382	Maternal overweight/obesity	3.00E-08	–	29016858
BMP6	Nitrogen dioxide exposure	2.50E-08	–	29410382	Maternal overweight/obesity	3.30E-12	–	29016858
SPATA13	Nitrogen dioxide exposure	2.50E-08	–	29410382	Maternal overweight/obesity	2.80E-10	–	29016858
LIN7A;MIR6 17	Nitrogen dioxide exposure	2.50E-08	–	29410382	Maternal overweight/obesity	7.90E-10	–	29016858
THBS2	Nitrogen dioxide exposure	2.50E-08	–	29410382	Maternal overweight/obesity	1.70E-09	–	29016858
NRP2	Nitrogen dioxide exposure	2.50E-08	–	29410382	Maternal overweight/obesity	2.60E-09	–	29016858
BMP1	Air pollution exposure, Nitrogen dioxide exposure	2.50E-08	–	26731791, 29410382	Maternal overweight/obesity	1.50E-08	–	29016858
ANK1	Nitrogen dioxide exposure	2.50E-08	–	29410382	Maternal overweight/obesity	2.80E-08	+	29016858
GPT2	Nitrogen dioxide exposure	2.60E-08	–	29410382	Maternal overweight/obesity	1.90E-09	–	29016858
MIR377	Nitrogen dioxide exposure	2.60E-08	–	29410382	Maternal overweight/obesity	4.10E-09	–	29016858
MYLK4	Nitrogen dioxide exposure	2.60E-08	–	29410382	Maternal overweight/obesity	2.40E-08	–	29016858
APOL6	Nitrogen dioxide exposure	2.60E-08	+	29410382	Maternal overweight/obesity	4.10E-08	+	29016858

Gene	Trait1	minP	Direction	PMID	Trait2	minP	Direction	PMID
LOC100132354	Air pollution exposure, Nitrogen dioxide exposure	2.70E-08	–	26731791, 29410382	Maternal overweight/obesity, Diastolic Blood Pressure, Systolic Blood Pressure	2.20E-21	–	29016858, 29198723
ACSL6	Nitrogen dioxide exposure	2.70E-08	–	29410382	Maternal overweight/obesity	9.20E-11	–	29016858
WNT8A	Air pollution exposure, Nitrogen dioxide exposure	2.70E-08	–	26731791, 29410382	Maternal overweight/obesity	1.20E-08	–	29016858
CORO2A	Nitrogen dioxide exposure	2.70E-08	–	29410382	Maternal overweight/obesity, fasting glucose	1.40E-08	–	29016858, 31197173
COL23A1	Nitrogen dioxide exposure	2.70E-08	–	29410382	Maternal overweight/obesity	1.50E-08	–	29016858
FAM170B	Nitrogen dioxide exposure	2.70E-08	–	29410382	Maternal overweight/obesity	2.50E-08	–	29016858
ERI3	Nitrogen dioxide exposure	2.70E-08	–	29410382	Maternal overweight/obesity	4.30E-08	–	29016858
PRAGMIN	Air pollution exposure, Nitrogen dioxide exposure	2.80E-08	+	26731791, 29410382	Maternal overweight/obesity	1.70E-09	–	29016858
BRUNOL4	Nitrogen dioxide exposure	2.80E-08	–	29410382	Maternal overweight/obesity	5.10E-09	–	29016858
IL12A	Nitrogen dioxide exposure	2.80E-08	–	29410382	Maternal overweight/obesity	6.80E-09	–	29016858
BRSK1	Nitrogen dioxide exposure	2.80E-08	–	29410382	Maternal overweight/obesity, Triglycerides, Triglycerides in small HDL	1.20E-08	–	29016858
MRAP	Nitrogen dioxide exposure	2.80E-08	–	29410382	Maternal overweight/obesity	1.80E-08	–	29016858

Gene	Trait1	minP	Direction	PMID	Trait2	minP	Direction	PMID
CNTNAP1	Nitrogen dioxide exposure	2.80E-08	–	29410382	Maternal overweight/obesity	2.30E-08	–	29016858
ACSF2	Nitrogen dioxide exposure	2.80E-08	–	29410382	Maternal overweight/obesity	4.40E-08	–	29016858
UNC5B	Nitrogen dioxide exposure	2.90E-08	–	29410382	Maternal overweight/obesity	8.70E-11	–	29016858
TRPM5	Nitrogen dioxide exposure	2.90E-08	–	29410382	Maternal overweight/obesity, Triglycerides in HDL, Triglycerides to total lipids ratio in large HDL	1.50E-10	–	29016858
LRRN1	Air pollution exposure, Nitrogen dioxide exposure	2.90E-08	–	26731791, 29410382	Maternal overweight/obesity	4.10E-10	–	29016858
SLC6A3	Nitrogen dioxide exposure	2.90E-08	–	29410382	Maternal overweight/obesity	5.30E-10	–	29016858
TMEM176B	Nitrogen dioxide exposure	2.90E-08	–	29410382	Maternal overweight/obesity	7.50E-10	–	29016858
MIR200A;MIR429	Nitrogen dioxide exposure	2.90E-08	–	29410382	Maternal overweight/obesity	1.60E-09	–	29016858
CYFIP1	Nitrogen dioxide exposure	2.90E-08	–	29410382	Maternal overweight/obesity	7.80E-09	–	29016858
MIR642;GIPR	Nitrogen dioxide exposure	2.90E-08	–	29410382	Maternal overweight/obesity	2.60E-08	–	29016858
CST9L	Nitrogen dioxide exposure	2.90E-08	–	29410382	Maternal overweight/obesity	3.40E-08	–	29016858
IARS2;MIR194-1;MIR215	Nitrogen dioxide exposure	2.90E-08	–	29410382	Maternal overweight/obesity	4.40E-08	–	29016858
GPR61	Nitrogen dioxide exposure	3.00E-08	–	29410382	Maternal overweight/obesity	6.50E-13	–	29016858

Gene	Trait1	minP	Direction	PMID	Trait2	minP	Direction	PMID
VWF	Nitrogen dioxide exposure	3.00E-08	–	29410382	Maternal overweight/obesity, 1-hour glucose	6.20E-11	–	29016858, 33151971
VPS13D	Nitrogen dioxide exposure	3.00E-08	–	29410382	Maternal overweight/obesity, fasting glucose	1.60E-10	–	29016858, 31197173
CHCHD6	Nitrogen dioxide exposure	3.00E-08	–	29410382	Maternal overweight/obesity	1.10E-09	–	29016858
FGGY	Nitrogen dioxide exposure	3.00E-08	–	29410382	Maternal overweight/obesity	1.60E-09	–	29016858
PEX14	Nitrogen dioxide exposure	3.00E-08	–	29410382	Maternal overweight/obesity	6.50E-09	–	29016858
ESYT3	Nitrogen dioxide exposure	3.00E-08	–	29410382	Maternal overweight/obesity	1.30E-08	–	29016858
GYLTL1B	Nitrogen dioxide exposure	3.00E-08	–	29410382	Maternal overweight/obesity	3.00E-08	–	29016858
BAIAP2	Air pollution exposure, Nitrogen dioxide exposure	3.10E-08	–	26731791, 29410382	Maternal overweight/obesity, Triglycerides to total lipids ratio in small HDL	1.30E-10	–	29016858
ERGIC1	Nitrogen dioxide exposure	3.10E-08	–	29410382	Maternal overweight/obesity	9.80E-10	–	29016858
ZDHHC14	Air pollution exposure, Nitrogen dioxide exposure	3.10E-08	–	26731791, 29410382	Diastolic Blood Pressure, Systolic Blood Pressure, Triglycerides	2.60E-08	–	29198723, 31910897
PRR5L	Air pollution exposure, Nitrogen dioxide exposure	3.20E-08	–	26731791, 29410382	Maternal overweight/obesity, fasting glucose, 1-hour glucose	2.60E-12	–	29016858, 31197173, 33151971

Gene	Trait1	minP	Direction	PMID	Trait2	minP	Direction	PMID
BCAM	Nitrogen dioxide exposure	3.20E-08	–	29410382	Maternal overweight/obesity, 1-hour glucose	2.20E-11	–	29016858, 33151971
ASB14	Nitrogen dioxide exposure	3.20E-08	–	29410382	Maternal overweight/obesity	8.20E-11	–	29016858
CYFIP2	Nitrogen dioxide exposure	3.20E-08	+	29410382	Maternal overweight/obesity	7.60E-09	+	29016858
LOXL1	Nitrogen dioxide exposure	3.20E-08	–	29410382	Maternal overweight/obesity	2.30E-08	–	29016858
KLHL26	Nitrogen dioxide exposure	3.30E-08	–	29410382	Maternal overweight/obesity	3.80E-10	–	29016858
CAMK2B	Nitrogen dioxide exposure	3.30E-08	–	29410382	Maternal diastolic blood pressure, Maternal systolic blood pressure	2.20E-08	NA	32078381
CDC42EP3	Nitrogen dioxide exposure	3.30E-08	–	29410382	Maternal overweight/obesity	3.10E-08	–	29016858
GRAMD1B	Nitrogen dioxide exposure	3.40E-08	–	29410382	Maternal overweight/obesity	7.80E-12	–	29016858
MPZ	Nitrogen dioxide exposure	3.40E-08	–	29410382	Maternal overweight/obesity	3.20E-11	–	29016858
AVPR1B	Nitrogen dioxide exposure	3.40E-08	–	29410382	Maternal overweight/obesity	1.50E-09	–	29016858
SLC28A1	Nitrogen dioxide exposure	3.40E-08	–	29410382	Maternal overweight/obesity	8.90E-09	–	29016858
SLC2A2	Nitrogen dioxide exposure	3.40E-08	–	29410382	Maternal overweight/obesity	1.80E-08	–	29016858
PAX5	Nitrogen dioxide exposure	3.40E-08	–	29410382	Maternal overweight/obesity	2.00E-08	–	29016858
SOX6	Nitrogen dioxide exposure	3.40E-08	+	29410382	Maternal overweight/obesity	3.90E-08	+	29016858

Gene	Trait1	minP	Direction	PMID	Trait2	minP	Direction	PMID
NR4A1	Nitrogen dioxide exposure	3.50E-08	–	29410382	Maternal overweight/obesity	5.20E-09	–	29016858
GNG2	Nitrogen dioxide exposure	3.50E-08	+	29410382	Maternal overweight/obesity	1.20E-08	–	29016858
MAP3K6	Nitrogen dioxide exposure	3.60E-08	–	29410382	Maternal overweight/obesity	2.10E-10	–	29016858
FMO3	Nitrogen dioxide exposure	3.60E-08	–	29410382	Maternal overweight/obesity	6.60E-10	–	29016858
SFRP1	Nitrogen dioxide exposure	3.60E-08	–	29410382	Maternal overweight/obesity	2.50E-09	–	29016858
ZBTB7B	Nitrogen dioxide exposure	3.60E-08	+	29410382	Maternal overweight/obesity, Diastolic Blood Pressure, Systolic Blood Pressure, fasting glucose	8.40E-09	+	29016858, 29198723, 31197173
RSAD1	Nitrogen dioxide exposure	3.70E-08	–	29410382	Maternal overweight/obesity	3.10E-09	–	29016858
VSIG2	Nitrogen dioxide exposure	3.70E-08	–	29410382	Maternal overweight/obesity	1.40E-08	–	29016858
TRPV4	Nitrogen dioxide exposure	3.70E-08	–	29410382	Maternal overweight/obesity	3.50E-08	–	29016858
PCSK6	Nitrogen dioxide exposure	3.80E-08	–	29410382	Serum triglycerides, Maternal overweight/obesity	4.90E-13	–	28173150, 29016858
AP3B2	Nitrogen dioxide exposure	3.80E-08	–	29410382	Maternal overweight/obesity	1.50E-12	–	29016858
LRRFIP1	Nitrogen dioxide exposure	3.80E-08	–	29410382	Maternal overweight/obesity, 1-hour glucose, Triglycerides to total lipids ratio in medium VLDL	1.30E-10	–	29016858, 33151971

Gene	Trait1	minP	Direction	PMID	Trait2	minP	Direction	PMID
CTCFL	Nitrogen dioxide exposure	3.80E-08	–	29410382	Maternal overweight/obesity	4.00E-09	–	29016858
RNU5E;RNU5D;CKMT2	Nitrogen dioxide exposure	3.80E-08	–	29410382	Maternal overweight/obesity	2.70E-08	–	29016858
C17orf101	Nitrogen dioxide exposure	3.80E-08	–	29410382	Maternal overweight/obesity	4.70E-08	–	29016858
JDP2	Nitrogen dioxide exposure	3.90E-08	–	29410382	Diastolic Blood Pressure, Systolic Blood Pressure	4.80E-10	–	29198723
MS4A15	Nitrogen dioxide exposure	3.90E-08	–	29410382	Maternal overweight/obesity	1.20E-09	–	29016858
KCNK3	Nitrogen dioxide exposure	3.90E-08	–	29410382	Maternal overweight/obesity	1.30E-08	–	29016858
OR1G1	Nitrogen dioxide exposure	3.90E-08	–	29410382	Maternal overweight/obesity	2.30E-08	–	29016858
UPK2	Nitrogen dioxide exposure	4.00E-08	–	29410382	Maternal overweight/obesity	7.90E-11	–	29016858
DLGAP1	Nitrogen dioxide exposure	4.00E-08	–	29410382	Maternal overweight/obesity	1.80E-09	–	29016858
CTNND2	Nitrogen dioxide exposure	4.00E-08	–	29410382	Maternal overweight/obesity	3.60E-09	–	29016858
ARHGEF16	Nitrogen dioxide exposure	4.00E-08	–	29410382	Maternal overweight/obesity	1.10E-08	–	29016858
GIF	Nitrogen dioxide exposure	4.00E-08	–	29410382	Maternal overweight/obesity	1.40E-08	–	29016858
FYN	Nitrogen dioxide exposure	4.00E-08	+	29410382	Maternal overweight/obesity	4.50E-08	+	29016858
CMTM2	Nitrogen dioxide exposure	4.10E-08	–	29410382	Maternal overweight/obesity	6.90E-12	–	29016858
TRAF1	Nitrogen dioxide exposure	4.10E-08	+	29410382	Maternal overweight/obesity	7.80E-10	+	29016858

Gene	Trait1	minP	Direction	PMID	Trait2	minP	Direction	PMID
PRSS21	Nitrogen dioxide exposure	4.10E-08	–	29410382	Maternal overweight/obesity	6.00E-09	–	29016858
MICA	Prenatal NO2 exposure, Nitrogen dioxide exposure	4.10E-08	–	27448387, 29410382	Maternal overweight/obesity, triglycerides	9.00E-09	–	29016858, 32677467
SEC14L1	Nitrogen dioxide exposure	4.20E-08	–	29410382	Maternal overweight/obesity	3.30E-10	–	29016858
BOC	Nitrogen dioxide exposure	4.20E-08	–	29410382	Maternal overweight/obesity	5.70E-09	–	29016858
LOC646498	Nitrogen dioxide exposure	4.20E-08	–	29410382	Maternal overweight/obesity	2.10E-08	–	29016858
BIN2	Nitrogen dioxide exposure	4.20E-08	+	29410382	Maternal overweight/obesity	3.20E-08	+	29016858
CMYA5	Nitrogen dioxide exposure	4.20E-08	–	29410382	Maternal overweight/obesity	3.50E-08	–	29016858
FMNL2	Nitrogen dioxide exposure	4.30E-08	–	29410382	Maternal overweight/obesity	5.10E-11	–	29016858
NCKAP5	Nitrogen dioxide exposure	4.30E-08	–	29410382	Maternal overweight/obesity, Triglycerides in very large HDL	9.80E-10	–	29016858
ZNF488	Nitrogen dioxide exposure	4.30E-08	–	29410382	Maternal overweight/obesity, 1-hour glucose	3.60E-08	–	29016858, 33151971
TRIO	Nitrogen dioxide exposure	4.30E-08	–	29410382	Maternal overweight/obesity	4.90E-08	–	29016858
ADAM30	Nitrogen dioxide exposure	4.40E-08	–	29410382	Maternal overweight/obesity, Triglycerides	1.80E-10	–	29016858
ITIH5	Nitrogen dioxide exposure	4.40E-08	–	29410382	Maternal overweight/obesity	8.00E-09	–	29016858

Gene	Trait1	minP	Direction	PMID	Trait2	minP	Direction	PMID
PAPSS1	Nitrogen dioxide exposure	4.40E-08	–	29410382	Maternal overweight/obesity	1.20E-08	–	29016858
LOC727677	Nitrogen dioxide exposure	4.50E-08	+	29410382	Maternal overweight/obesity	1.40E-10	+	29016858
BMPER	Nitrogen dioxide exposure	4.50E-08	–	29410382	Maternal overweight/obesity	1.50E-10	–	29016858
TNKS1BP1	Nitrogen dioxide exposure	4.50E-08	–	29410382	Maternal overweight/obesity	9.30E-10	–	29016858, 32916427
RHO	Nitrogen dioxide exposure	4.50E-08	–	29410382	Maternal overweight/obesity	1.10E-09	–	29016858
XIRP1	Nitrogen dioxide exposure	4.50E-08	–	29410382	Maternal overweight/obesity, fasting glucose	1.90E-09	–	29016858, 31197173
HLA-DRA	Nitrogen dioxide exposure	4.50E-08	–	29410382	Maternal overweight/obesity	1.10E-08	–	29016858
BFSP2	Nitrogen dioxide exposure	4.50E-08	–	29410382	Maternal overweight/obesity	1.40E-08	–	29016858
DENND2D	Nitrogen dioxide exposure	4.50E-08	+	29410382	Maternal overweight/obesity	2.30E-08	+	29016858
RETNLB	Nitrogen dioxide exposure	4.60E-08	–	29410382	Maternal overweight/obesity	5.20E-10	–	29016858
SSPO	Nitrogen dioxide exposure	4.60E-08	–	29410382	Maternal overweight/obesity	2.10E-08	–	29016858
NTRK1	Nitrogen dioxide exposure	4.70E-08	–	29410382	Maternal overweight/obesity	1.70E-10	–	29016858
AK5	Nitrogen dioxide exposure	4.70E-08	–	29410382	Maternal overweight/obesity, Triglycerides in large HDL	3.90E-09	–	29016858

Gene	Trait1	minP	Direction	PMID	Trait2	minP	Direction	PMID
TRPV1	Nitrogen dioxide exposure	4.70E-08	–	29410382	Maternal overweight/obesity, fasting glucose, Triglycerides	9.10E-09	–	29016858, 31197173, 31910897
CDH22	Nitrogen dioxide exposure, Particulate matter air pollution	4.70E-08	–	29410382, 32484729	Maternal overweight/obesity, fasting glucose	1.20E-08	–	29016858, 31197173
LOC149134	Nitrogen dioxide exposure	4.70E-08	–	29410382	Maternal overweight/obesity	2.70E-08	–	29016858
SH3TC1	Nitrogen dioxide exposure, Particulate matter ≤10 um (PM10)	4.80E-08	–	29410382, 31208937	Maternal overweight/obesity	1.70E-08	–	29016858
KCNIP3	Nitrogen dioxide exposure	4.80E-08	–	29410382	Maternal overweight/obesity	3.60E-08	–	29016858
RNF213;LOC100294362	Air pollution exposure, Nitrogen dioxide exposure, Nitrogen dioxide air pollution	4.80E-08	–	26731791, 29410382, 32484729	Maternal overweight/obesity	3.70E-08	–	29016858
GALM	Nitrogen dioxide exposure	4.90E-08	–	29410382	Maternal overweight/obesity	4.60E-09	–	29016858
SH2D6	Nitrogen dioxide exposure	4.90E-08	–	29410382	Maternal overweight/obesity	3.20E-08	–	29016858

Minimum P value of methylation sites on each gene is given (minP), alongside with its corresponding effect direction. All results are from whole/cord blood samples. AP: Air Pollution; PM: Particulate Matter.
